# Telehealth Interventions to Support Self-Management of Long-Term Conditions: A Systematic Metareview of Diabetes, Heart Failure, Asthma, Chronic Obstructive Pulmonary Disease, and Cancer

**DOI:** 10.2196/jmir.6688

**Published:** 2017-05-17

**Authors:** Peter Hanlon, Luke Daines, Christine Campbell, Brian McKinstry, David Weller, Hilary Pinnock

**Affiliations:** ^1^ Usher Institute of Population Health Sciences and Informatics University of Edinburgh Edinburgh United Kingdom; ^2^ Allergy and Respiratory Research Group Usher Institute of Population Health Sciences and Informatics University of Edinburgh Edinburgh United Kingdom; ^3^ E-Health Group Usher Institute of Population Health Sciences and Informatics University of Edinburgh Edinburgh United Kingdom

**Keywords:** telehealth, telemonitoring, self-management, chronic disease, diabetes, heart failure, asthma, COPD, pulmonary disease, chronic obstructive, cancer

## Abstract

**Background:**

Self-management support is one mechanism by which telehealth interventions have been proposed to facilitate management of long-term conditions.

**Objective:**

The objectives of this metareview were to (1) assess the impact of telehealth interventions to support self-management on disease control and health care utilization, and (2) identify components of telehealth support and their impact on disease control and the process of self-management. Our goal was to synthesise evidence for telehealth-supported self-management of diabetes (types 1 and 2), heart failure, asthma, chronic obstructive pulmonary disease (COPD) and cancer to identify components of effective self-management support.

**Methods:**

We performed a metareview (a systematic review of systematic reviews) of randomized controlled trials (RCTs) of telehealth interventions to support self-management in 6 exemplar long-term conditions. We searched 7 databases for reviews published from January 2000 to May 2016 and screened identified studies against eligibility criteria. We weighted reviews by quality (revised A Measurement Tool to Assess Systematic Reviews), size, and relevance. We then combined our results in a narrative synthesis and using harvest plots.

**Results:**

We included 53 systematic reviews, comprising 232 unique RCTs. Reviews concerned diabetes (type 1: n=6; type 2, n=11; mixed, n=19), heart failure (n=9), asthma (n=8), COPD (n=8), and cancer (n=3). Findings varied between and within disease areas. The highest-weighted reviews showed that blood glucose telemonitoring with feedback and some educational and lifestyle interventions improved glycemic control in type 2, but not type 1, diabetes, and that telemonitoring and telephone interventions reduced mortality and hospital admissions in heart failure, but these findings were not consistent in all reviews. Results for the other conditions were mixed, although no reviews showed evidence of harm. Analysis of the mediating role of self-management, and of components of successful interventions, was limited and inconclusive. More intensive and multifaceted interventions were associated with greater improvements in diabetes, heart failure, and asthma.

**Conclusions:**

While telehealth-mediated self-management was not consistently superior to usual care, none of the reviews reported any negative effects, suggesting that telehealth is a safe option for delivery of self-management support, particularly in conditions such as heart failure and type 2 diabetes, where the evidence base is more developed. Larger-scale trials of telehealth-supported self-management, based on explicit self-management theory, are needed before the extent to which telehealth technologies may be harnessed to support self-management can be established.

## Introduction

The rising prevalence of long-term conditions is a major clinical and public health challenge [1]. Telehealth has attracted considerable interest as a means of delivering care to those with long-term conditions [2]. Definitions of telehealth are many and varied [3], and the technologies involved are novel and evolving [4]: in this paper we use the term to refer to *any use of information and communication technology to facilitate communication or transfer of information between patient and health care provider over a distance* [5]. Self-management—whereby individuals take on tasks to deal with medical management, role management, or emotional aspects of their condition [6]—is also increasingly recognized as important for effective management of long-term conditions [7-9]. Telehealth has been proposed as one mechanism by which self-management may be promoted and enabled [10], although, in contrast, it has also been suggested that telehealth may sometimes increase dependence on health professionals rather than promoting self-management [11]. The extent to which telehealth effectively promotes self-management, and the components of telehealth interventions that contribute to this goal, remain unclear.

Self-management and its support comprise a wide range of potential activities and interventions [12]. While self-management support is an important aspect of the wider management of a range of long-term conditions, its nature and the approach to supporting successful self-management varies depending on the condition, as well as the individual patient. A systematic overview of self-management interventions (Practical Reviews in Self-Management Support [PRISMS]) demonstrated that self-management support interventions across a range of 14 long-term conditions are complex and multifaceted, involve both the patient and health care professional, and need to be tailored to the individual and their specific condition and context [12]. The review found that no single component of self-management interventions could be identified as being more important than others, but also that the detail and quality of reporting of these complex interventions was a barrier to their wider implementation and the understanding of their effective components [12,13]. This observation, along with the lack of a suitable tool to analyze the important components of self-management interventions, led to the development of the PRISMS taxonomy of self-management support [14]. This taxonomy identified 14 separate components that might be adapted and used to support self-management across a range of long-term conditions. Several of these components could be potentially delivered via telehealth, and may be grouped and considered under the following headings:

- Patient education and information provision
- Remote monitoring with feedback and action plans (eg, peak expiratory flow or blood glucose monitoring with action plans)
- Telehealth-facilitated clinical review
- Adherence support (eg, medication or lifestyle intervention adherence)
- Psychological support
- Lifestyle interventions (eg, smoking cessation, exercise, weight loss)

Given the wide variety of technologies and interventions that telehealth encompasses [2], and the varied nature of self-management interventions [12], we aimed to gain a broad overview of the evidence for telehealth-mediated self-management support using metareview methodology [15]. We focused on 6 specific conditions in which telehealth has been widely used and evaluated as a method of delivering care and in which the principles of self-management are considered an important component of disease management [12,16]. With respect to telehealth-supported self-management, we aimed to (1) assess the impact on disease control and health care utilization, and (2) identify components of self-management support delivered by the telehealth interventions and assess the impact of these components on disease control and the process of self-management.

## Methods

This metareview aimed to synthesize systematic review evidence on telehealth interventions to support self-management in diabetes mellitus (types 1 and 2), heart failure, asthma, chronic obstructive pulmonary disease (COPD), and cancer. We carried it out according to a prespecified protocol. We were unable to register our protocol as PROSPERO (an international prospective register of systematic reviews) does not accept protocols for metareviews.

**Table 1:** Inclusion criteria and database search for systematic reviews of randomized controlled trials (RCTs) of telehealth interventions incorporating components of supported self-management.

Criteria	Description
Population	Adults or children with 1 or more specified long-term conditions (diabetes mellitus type 1 or 2, heart failure, asthma, chronic obstructive pulmonary disease, and cancer).
	Reviews of multiple conditions included if disease-specific findings reported separately.
Intervention	Telehealth^a^ interventions to support self-management^b^.
Comparator	“Usual care” or alternative means of delivering the intervention (eg, face-to-face, paper-based).
Outcomes	Markers of disease control (see Table 2).
	Unscheduled use of health care services (see Table 2).
	Self-management process outcomes (see Table 2).
Settings	Any health care setting.
Study design	Systematic reviews of RCTs.
	Systematic reviews of multiple study designs included if RCT data reported separately.
Databases	MEDLINE, Embase, CINAHL, PsycINFO, AMED, Web of Science, and Cochrane Database of Systematic Reviews.
Manual searching	Reference lists of all eligible reviews searched.
Forward citations	Performed for all included systematic reviews (using Web of Science).
In-progress studies	Abstract used to identify recently published reviews.
Restrictions	No language restriction applied.
Dates	Initial search: January 2000 to November 2014 (limited to studies later than 2000 due to the relatively recent introduction of the technological solutions and the rapid rate of development of the field. Few studies prior to 2000 were identified in scoping search).
	Update search: May 2016.
	Forward citation search: completed June 2016.
Other exclusions	Less detailed versions of Cochrane reviews published (data taken from the Cochrane review).
	Previous versions of reviews that had been subsequently updated.
	Reviews lacking analyses of quantitative RCT data (narrative or meta-analysis).
	Interventions in which there was no transfer of clinical information between patient and health care provider (eg, peer-to-peer online forums), or where evidence of this was not clear (eg, computer- or Internet-based interventions that gave or recorded information without transfer).

^a^Telehealth was defined as any intervention in which clinical information is transferred remotely between patient and health care provider, regardless of the technology used to record or transmit the information.

^b^Self-management was defined as any intervention that aimed to empower patients to be active decision makers who deal with emotional, social, or medical management of their illness with the aim of improving their independence and quality of life.

### Search Strategy

Following an initial scoping, we searched for systematic reviews of randomized controlled trials (RCTs) of telehealth interventions incorporating components of supported self-management. The basic search strategy combined “telehealth terms” AND “self-management terms” AND “long term conditions terms” AND “systematic review terms.” The search used a combination of keyword searches and Medical Subject Headings (MeSH). We searched 7 databases for reviews published from January 2000 to May 2016. The search was limited to studies published later than 2000 because telehealth is a relatively recent innovation and rapid advances in technology mean that any earlier work is unlikely to be relevant to contemporary health care. Table 1 summarizes the search strategy and sources, and Table 2 details the outcomes. Multimedia Appendix 1 shows full search terms for the MEDLINE database; we adjusted these for the other databases.

**Table 2:** Outcomes and definitions.

Outcome		Definition
**Markers of disease control**		
	Nondisease specific	Mortality
		Symptoms and exacerbations: reported symptoms or symptom scores
		Measured rate or frequency of exacerbations
		Other (not disease-specific) biological markers such as blood pressure, lipids
	Disease specific	Diabetes: hemoglobin A_1c_
		Heart failure: body weight, exercise tolerance
		Asthma: PEF^a^, FEV_1_^b^, etc
		Chronic obstructive pulmonary disease: FEV_1_
		Cancer: recurrence
**Health care utilization**		
	Health service utilization	Use of health care services (eg, admissions, length of stay, use of unscheduled services or emergency department)
**Self-management process outcomes**		
	Self-efficacy	The confidence that an individual has in their own ability to perform a specific task or behavior
	Self-management behaviors	Measures of self-management adoption behavior (eg, use of or adherence to action plan, medication adherence, frequency of monitoring [PEF, blood glucose, etc], avoidance of triggers, use of environmental resources)
**Quality of Life**		
	Quality of life	As assessed by validated tool
	Disease-specific quality of life	As assessed by validated quality-of-life assessment tool (eg, asthma quality-of-life score, St George’s respiratory questionnaire)

^a^PEF: peak expiratory flow rate.

^b^FEV_1_: forced expiratory volume in the first second of expiration.

Inclusion and exclusion criteria are summarized using the population, intervention, comparison, outcomes, setting, and study design headings in Table 1 and Table 2. Initial scoping revealed that few reviews of telehealth interventions identified self-management support explicitly as an aim of the telehealth intervention under consideration (eg, self-management was a specified inclusion criterion). We therefore also included reviews in which self-management support was an implied component or mechanism of the telehealth intervention under consideration and in which outcomes relevant to self-management were assessed as part of the review. For the purpose of inclusion, we defined self-management as “any intervention which aimed to empower patients to be active decision makers who deal with emotional, social or medical management of their illness with the aim of improving their independence and quality of life” [6]. We excluded reviews when the telehealth aspect simply involved remote physiological monitoring (eg, of oxygen saturation or blood sugar levels) without an explicit decision-making role on the part of the patient. Reviews in which patients were educated and supported to interpret and act on the clinical information they were recording we considered self-management and therefore included. We included reviews in which self-management was hypothesized as the mechanism by which the telehealth intervention had an impact, but we considered these to be implied self-management unless self-management support was also a specified aim of the intervention.

### Screening of Titles, Abstracts, and Full Texts

Inclusion criteria were piloted by 2 authors (PH and HP) and disagreements were resolved by discussion with all authors. PH then assessed all titles and abstracts against the inclusion criteria. Where no abstract was available, articles were retained for full-text assessment. A random sample of 250 abstracts was screened by 2 reviewers (PH and LD). A kappa statistic of agreement was calculated using IBM SPSS version 22 (IBM Corporation) and was high (0.96). Full texts of all potentially eligible articles were assessed independently by 2 reviewers (PH and LD).

### Weighting and Quality Assessment

We assessed the quality of the included reviews using the revised A Measurement Tool to Assess Systematic Reviews (R-AMSTAR) quality assessment tool. Each included review was assessed independently by 2 reviewers (PH and LD) and disagreements were resolved by discussion. We combined the R-AMSTAR score with the size of the review and an assessment of self-management focus to assign a star-based weighting to the evidence from each review. We awarded 1 star for each of the following:

- R-AMSTAR score >30
- >1000 participants (or >10 RCTs if information on participants was not available)
- Explicit self-management focus (ie, self-management support was specified in the inclusion criteria of the systematic review)

We then used the weighting of each review to inform the synthesis. Any disagreements in the full-text screening, quality assessment, or data extraction were resolved by discussion, involving a third author when agreement could not be reached.

### Outcomes

We grouped outcomes of interest into disease control outcomes (clinical and physiological markers of disease control, unscheduled health care utilization, and validated measures of symptoms and quality of life) and self-management process outcomes (eg, self-efficacy, medication adherence). These are defined in Table 2.

### Data Extraction and Interpretation

Data were extracted using a piloted data extraction template for included studies (PH), and each study was checked for accuracy (LD). We extracted the description of intervention(s) and component(s) of self-management support; inclusion and exclusion criteria; population of interest; duration and intensity of intervention; outcomes measured; and results as presented in the review. The synthesis and conclusions of each review were collated: we did not analyze results from individual RCTs.

The overlap in included RCTs (ie, reviews with similar inclusion criteria may include the same RCTs) precluded meta-analysis of the review findings; we therefore undertook a narrative synthesis. We used harvest plots to illustrate the disease control outcomes related to telehealth-supported self-management components [17,18]. Harvest plots use bars representing individual reviews placed on a plot matrix to indicate whether the review intervention showed an overall positive, negative, or no consistent effect for the outcome in question. To construct the harvest plots, we needed to judge each review as to whether it showed an overall positive effect for each outcome or group of outcomes. Given the heterogeneity in outcomes and methods of data synthesis among the included reviews, and to ensure objective and consistent assessment of each review, we devised a set of rules to underpin decisions about whether a review showed a positive, negative, or no effect. These were devised and refined by discussion between the authors and are described in Table 3. Interpretation of the findings was aided by regular discussion within the research team.

## Results

### Search Results

**Figure 1:**
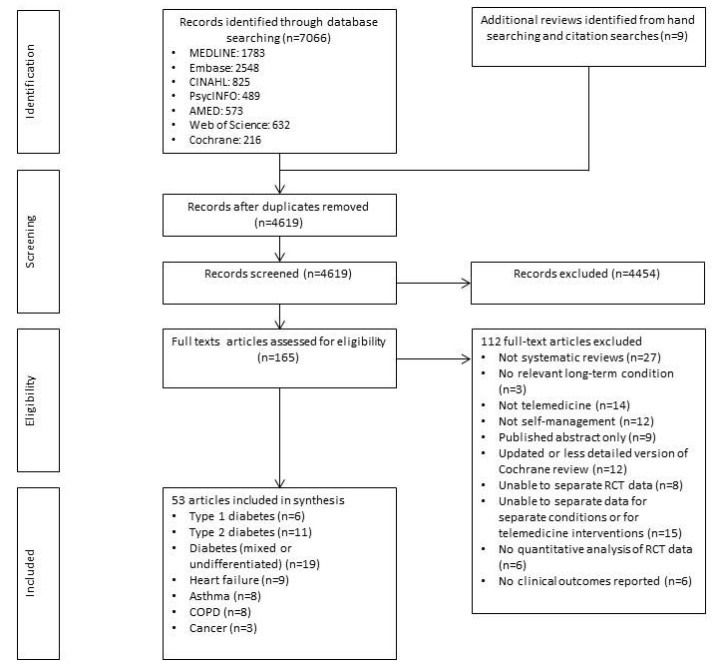
Preferred Reporting Items for Systematic Reviews and Meta-analyses (PRISMA) diagram of literature search. COPD: chronic obstructive pulmonary disease; RCT: randomized controlled trial.

The Preferred Reporting Items for Systematic Reviews and Meta-analyses (PRISMA) flowchart shown in Figure 1 illustrates the search results and review selection.

A total of 53 systematic reviews met the inclusion criteria [16,19-70]. These presented data from 231 unique RCTs (119 diabetes, 58 heart failure, 28 asthma, 23 COPD, and 3 cancer). The year of publication ranged from 2000 to 2016. Information on the geographical spread of the RCTs included within the SRs was incomplete, but included studies from North and South America, Europe, Asia, and Oceania. Multimedia Appendix 2 lists the RCTs included in each systematic review.

### Study Characteristics

Multimedia Appendix 3 shows details of participant demographics, interventions and comparison, setting, and content and intensity of interventions for each of the included systematic reviews.

**Table 3:** Rules for assessment of systematic reviews (SRs) for analysis in harvest plots.

Rule no.	Rules as applied
1	If a review contains a meta-analysis, this result will be used in the harvest plot prioritized over the results of a narrative synthesis.
2	Where a review reports multiple meta-analyses of related outcomes (eg, mean HbA_1c_^a^ concentration; proportion of participants with a normal HbA_1c_; HbA_1c_ at different time points), and when these outcomes show conflicting results, the result of the SR’s primary outcome takes priority as the overall result of the review. Where reviews have no, or more than 1, primary outcome, then the review will be considered as having an overall positive effect if >50% of reported outcomes (or of primary outcomes if multiple) show a positive effect.
3	Where no meta-analysis is available and the review contains a narrative synthesis, overall effect will be judged by the proportion of studies reporting statistically significant positive effects in relevant outcomes. Between 0% and 50% of studies showing positive results will be shown as no consistent effect. Those with >50% of studies showing a positive effect will be shown as positive overall, with those between 50% and 75% hatched to indicate inconsistency. As for meta-analysis in the event of multiple analyses of related outcomes, the result of the SR’s defined primary outcome takes priority.
4	If a review reports positive results for an outcome, but it is not clear from the review how many studies in total measured that outcome (ie, no denominator is available), then this outcome will not be included on the harvest plot on grounds of incomplete data. These results will be displayed as reported in the table of review results.

^a^HbA_1c_: hemoglobin A_1c_.

### Quality and Weighting of Included Reviews

R-AMSTAR scores for the included systematic reviews ranged from 19 to 43 out of a possible 44. Multimedia Appendix 4 gives scores for the individual components of the R-AMSTAR score. Taking into account quality assessment, explicit self-management focus, and total population size, 8 reviews received an evidence weighting of 3 stars [16,21,25,26,38,45,53,56], 23 received 2 stars [22,24,27-31, 33,37,40,42,44,46,49,50,52,54,55,58,62,63,67,68], 21 received 1 star [19,20,23,32,34-36,39,43,47,48,51,57, 59-61,64-66,69,70], and 1 received no stars [41]. The first column of the table in Multimedia Appendix 5 displays these criteria.

### Overview of Presentation of Results

Multimedia Appendix 5 provides an overview of the focus, quality, findings, and conclusions of each of the included reviews. It also displays how the interventions described map to Pearce et al’s taxonomy of self-management support [14]. Additional detail is shown in Multimedia Appendix 6. The text that follows synthesizes the findings against the 2 aims of the metareview.

### Impact of Telehealth Interventions on Disease Control and Health Care Utilization

#### Diabetes

A total of 5 reviews focused on type 1 diabetes [23,24,41,48,49] and 1 analyzed type 1 diabetes within a mixed review [19]. The reviews of de Jongh et al [24] and Viana et al [49] were both weighted 2 stars. The former focused on mobile messaging interventions, including medication reminders, and found no improvement in hemoglobin A_1c_ (HbA_1c_) compared with usual care in a meta-analysis of 2 RCTs [24]. Viana et al meta-analyzed adherence support interventions using various technologies and also found no improvement in HbA_1c_ [49]. Three 1-star narrative syntheses showed improvement in HbA_1c_ limited to only a few RCTs, and all concluded that further evaluation was required [19,23,48]. A meta-analysis scoring no stars in the weighting system also showed no improvement in glycemic control [41].

A total of 10 reviews [21,29,30,33,42,43,46,52-54], and 1 analysis in a mixed review [19], analyzed HbA_1c_ in type 2 diabetes. Of these, 2 were awarded a 3-star weighting [21,53]. Wu et al meta-analyzed 7 RCTs of telephone follow-up and found no significant improvement in HbA_1c_ compared with usual care in the overall pooled analysis [53]. However, a prespecified subgroup analysis of more intensive targeted interventions (n=3 RCTs) showed a statistically significant benefit on glycemic control. Beatty et al reviewed Web-based lifestyle interventions and found no impact on glycemic control [21]. Of seven 2-star reviews [29,30,33,42,46,52,54], 4 were meta-analyses, all of which showed significant reductions in HbA_1c_ compared with controls, although the effect sizes were small. Interventions included telemonitoring of blood glucose [33,42,54] and mobile messaging [46]. Narrative syntheses found that telehealth-delivered educational interventions [52] and telephone interventions [29] did not improve glycemic control. Greenwood et al analyzed the components of remotely monitored blood glucose and found that multifaceted interventions carried greater benefit (discussed under self-management components) [30]. Two 1-star reviews (1 of mobile telemonitoring and 1 of Web-based telemonitoring) showed improved HbA_1c_ compared with control [19,43].

In total, 19 reviews included both type 1 and 2 diabetes in their analyses, or included RCTs that did not differentiate [16,22,25,27,28,31,32,34-40,44,45,47,50,51]. Of these, 4 were awarded a 3-star weighting [16,25,38,45], of which Farmer et al [25] *,* Small et al [45], and Liang et al [38] analyzed control or health care utilization outcomes, or both. Farmer et al assessed telemonitoring of self-monitored blood glucose and found no significant improvement in HbA_1c_ in a meta-analysis of 9 RCTs, and either no difference or an increase in health care utilization in 6 RCTs [25]. Liang et al evaluated mobile phone interventions targeting glycemic control and found a significant improvement over usual care in a meta-analysis of 11 RCTs. This effect was more marked for type 2 than for type 1 diabetes [38]. Small et al reviewed telephone-only interventions incorporating “lay health workers” and showed a small but significant improvement in HbA_1c_ over usual care [45]. A total of 8 reviews were weighted 2 stars [22,27,28,31,37,40,44,50]; 4 of these included meta-analyses of impact on HbA_1c_, with 3 showing significant improvements over controls. Interventions included telemonitoring of blood sugar [40,44] and interactive telehealth excluding telephone support [27]. A meta-analysis of teleconsultations showed no benefit in terms of HbA_1c_ [50]. Narrative syntheses showed mixed results, with modest benefit in telehealth interventions both in place of and supplementing usual care [37], little evidence of benefit from telehealth interventions that excluded telephone support [28], and positive results for telehealth-delivered behavioral interventions (n=13 RCTs) [22]. Three 1-star narrative syntheses suggested improved outcomes with mobile phone interventions [32,35,36], but others showed no overall benefit from teleconsultations [47,51] or telemonitoring [34,39].

Overall, the evidence for diabetes suggests that telehealth-supported self-management interventions for type 2 diabetes may be effective, with evidence for type 1 suggesting no overall benefit in glycemic control. Active self-monitoring of blood glucose data appeared to be most consistently associated with improved outcomes, although this was not consistent across reviews. Evidence for telephone support was more limited, although it may be effective as part of intensive interventions.

#### Heart Failure

A total of 9 reviews analyzed telehealth interventions for heart failure [16,20,28,55-60]. The highest weighted of these (Ciere et al, 3 stars) did not include any disease control outcomes [56], nor did 3 others [16,59,60]. The highest-weighted meta-analysis was Inglis et al [58]. This review separately analyzed structured telephone support and telemonitoring of physiological parameters. Self-management support was explicitly hypothesized as a mechanism by which these interventions might exert their effect, and both interventions reduced all-cause mortality. A sensitivity analysis of the telephone-based interventions showed no difference between symptom monitoring and education-focused telephone calls. Heart failure hospitalizations, but not all-cause hospital admissions, were also significantly lower with either telemonitoring or telephone support.

In contrast, a 2-star narrative review showed no mortality benefit from telephone-only interventions and a variable effect on hospital admissions [55]. The use of telehealth, without telephone support, showed no impact on health care utilization in a 2-star weighted narrative synthesis of 6 RCTs [28]. A 1-star weighted meta-analysis of telemonitoring for heart failure (excluding telephone interventions) showed a significant reduction in mortality and heart failure admissions, but not all-cause admissions or emergency department visits [57].

#### Asthma

A total of 8 reviews assessed the impact on asthma control through symptom scores, quality of life, or unscheduled health care [20,24,27,28,36,61-63]. The highest weighted of these each scored 2 stars [24,27,62,63], including 3 narrative syntheses and 1 meta-analysis. McLean et al analyzed a wide range of telehealth interventions, including structured telephone support, education support, telemonitoring, and action plan components [63]. Meta-analyses showed no significant improvement in emergency department use or hospitalization at 3 months, but a significant reduction in hospitalizations compared with usual care at 12 months. Some studies reported improvements in symptom scores; however, most showed no benefit. The authors concluded that benefits were unlikely in mild asthma but that those at higher risk of hospitalization may benefit. de Jongh et al [24] and Marcano Belisario et al [62] reviewed mobile interventions and smartphone apps, respectively. The number of included RCTs was small for both (1 and 2, respectively). Both highlighted the potential benefit from some positive findings in the studies but acknowledged that these findings were not consistent and that the evidence base required development. A 2-star weighted review of multiple conditions included a narrative synthesis of 5 RCTs of interactive telehealth interventions, excluding telephone-only support, and concluded no overall evidence of benefit in asthma [27]. Three 1-star reviews, all with narrative syntheses, concluded that further evaluation was needed before conclusions could be reached [28,36,61], while 1 other, focused on mobile interventions in developing countries, found evidence for improved symptom scores in a single RCT [20].

#### Chronic Obstructive Pulmonary Disease

Of the 8 COPD reviews, 7 analyzed the impact on disease control [27,64-69]. These included 2 meta-analyses, both weighted 2 stars [67,68]. A total of 4 reviews (3 meta-analyses and 1 narrative syntheses) analyzed the impact of telehealth on all-cause mortality, and none showed a significant difference versus usual care [65,66,68,69,71,72]. Both 2-star meta-analyses concerned home-based telehealth using a variety of technologies, incorporating information transmission with personalized feedback. Lundell et al showed a significant improvement in physical activity with telehealth, but no impact on dyspnea [67]. McLean et al found significantly fewer emergency department visits and hospital admissions than with usual care. Findings for quality of life assessed by St George’s Respiratory Questionnaire were inconsistent [68]. The authors emphasized that, despite some positive findings, the telehealth interventions were components in complex interventions and further evaluation would be required to clarify their role in COPD [68]. Another meta-analysis, weighted 1 star, showed no impact on mortality but evidence of fewer hospitalizations in a narrative synthesis [69]. Four 1-star weighted narrative syntheses emphasized either a lack [27,64,65] or a low quality [66] of evidence for improved health outcomes.

#### Cancer

Of the 3 reviews that included cancer RCTs, 2 contained analyses of physical outcomes [21,70]. Both reviews were 2-star weighted. One showed no evidence of improved quality of life or emotional or physical wellbeing in an RCT of moderated Internet-based self-help for breast cancer patients [21]. The other review analyzed Internet-based education programs linking patients with clinicians and found no improvement in quality of life in 2 RCTs and an improvement in symptom scores in 1 of the RCTs [70].

**Figure 2:**
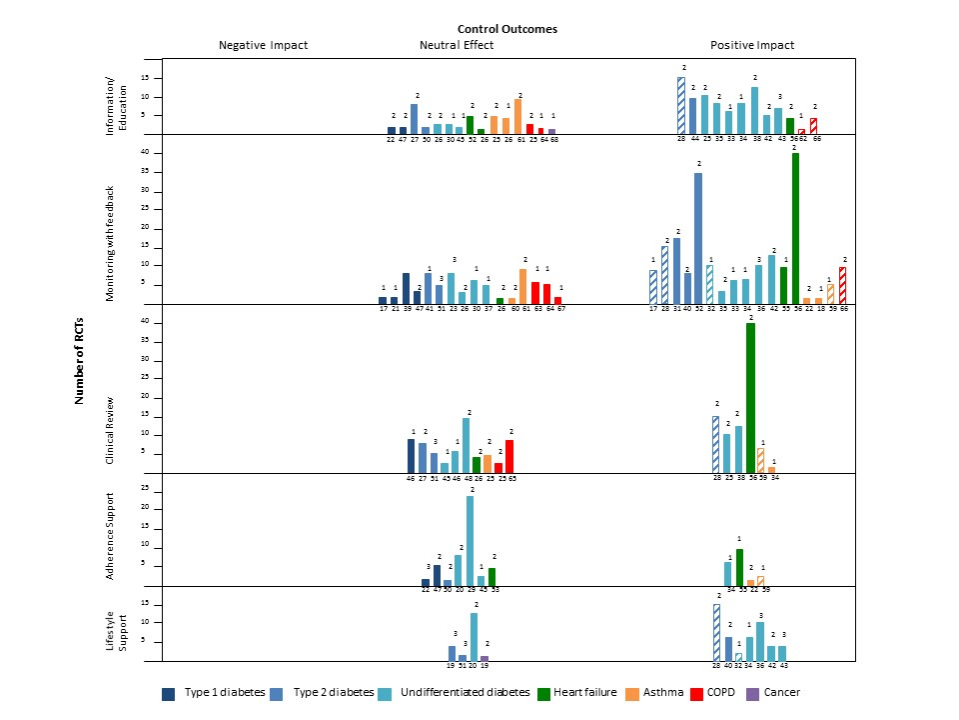
Harvest plot of overall findings of reviews. Number below bar: review reference number. Number above bar: star weighting of review (based on size, revised A Measurement Tool to Assess Systematic Reviews score, and explicit self-management focus). Height of bar: number of randomized controlled trials (RCTs) concerning that self-management component. Block color: consistent effect. Hatched: inconsistent effect (see Table 3). Outcomes assessed were diabetes (hemoglobin A_1c_), heart failure (mortality, hospital admission), asthma and chronic obstructive pulmonary disease (COPD) (validated symptom or quality of life, scores, physiological measurements), and cancer (validated symptom or quality of life).

### Telehealth-Supported Components of Self-Management and Their Impact on Disease Control and the Process of Self-Management

The overall findings of the reviews for impact on disease control outcomes of telehealth-supported components of self-management are illustrated in the harvest plot shown in Figure 2.

All of the interventions in the included systematic reviews were complex interventions with multiple components. Reporting of the details of the components of interventions was highly variable, and we therefore limited analyses of which specific components were associated with improvements in disease outcomes to a subset of the included systematic reviews. No single self-management component was found to be consistently effective, or consistently ineffective.

#### Education and Information

Supported education and information interventions were particularly effective in the context of diabetes, with 9 larger systematic reviews showing evidence of improvement in HbA_1c_ [27,30,35-37,40,44-46]. Similar interventions in other conditions showed either no evidence of benefit or inconsistent positive effects. The highest weighted of the heart failure reviews (Ciere et al) specifically analyzed whether the beneficial effects of telehealth interventions on clinical outcomes were mediated by increases in knowledge, self-care, or self-efficacy [56]. The authors tested the 2 hypotheses that, first, increased monitoring by health care providers and, second, improved knowledge or self-efficacy leading to improved self-management by patients were mechanisms by which telehealth interventions may be effective. They found that evidence linking telehealth interventions with increased knowledge or self-care behaviors, or linking self-efficacy with self-care was “ambiguous,” and concluded that, on the basis of their findings and the poor methodological quality of the included studies, the evidence neither supports nor refutes their models [56].

#### Monitoring and Feedback

This was the most commonly described telehealth component in the included systematic reviews and was associated with improved clinical outcomes in diabetes and heart failure [33,35,36,38,42,44,54,58]; however, findings for asthma and COPD were mostly neutral or inconsistent [61-63,65,66,68,69].

#### Facilitation of Remote Clinical Review

Teleconsultations, videoconferencing, and telephone follow-up designed to review symptoms or clinical course were important aspects of telehealth interventions described by Inglis et al for heart failure, whose meta-analysis showed reduced mortality and heart failure hospital admission with telemonitoring and telephone review [58]. Several diabetes reviews included elements of remote clinical review, with some reporting positive outcomes [27,30,40]. In asthma and COPD, the findings were typically neutral [27,67].

#### Adherence Support and Lifestyle Interventions

Fewer reviews focused on adherence support or lifestyle interventions, and, of those that did, several did not report disease control outcomes. Findings were more mixed than for other components. Achieving improved clinical outcomes with such interventions may be challenging, as they involve significant behavior change. A 3-star weighted review by Farmer et al analyzed the impact on medication adherence of interventions using remote monitoring, messaging, or a combination [26]. Meta-analysis of 8 interventions showed a nonsignificant effect size; and only 6 of the 15 interventions reported some improvement in medication adherence. The impact of this on glycemic control or other outcomes was not reported. Cassimatis et al (2 stars) analyzed behavioral interventions (excluding telemonitoring) and found some improvements in self-care, dietary and medication adherence, and physical activity, but the effect was inconsistent [22].

#### Multicomponent and Intensive Interventions

While it was clear that most telehealth interventions were complex multicomponent interventions, most of the included reviews either provided limited description of the interventions or did not specifically analyze the impact of individual components on the efficacy of the intervention as a whole. The only review to address explicitly the question of which components were associated with improved disease outcomes was Greenwood et al, weighted 2 stars [30]. They defined 7 separate components of telehealth (patient education, health care provider education, self-monitoring profile, blood glucose goals, use of blood glucose data to modify behavior, feedback to patients, and 2-way interaction). No interventions were found incorporating all 7 components; however, those including 5 or more were associated with significant improvement in HbA_1c_. The authors concluded that a range of these components need to be incorporated into telehealth interventions for clinically significant improvements in diabetes self-management to be seen.

While the intensity of the interventions included in many of the reviews varied widely between the included RCTs (Multimedia Appendix 3), few reviews specifically analyzed the relationship between the intensity (in terms of either contact with health care professionals or the complexity or number of components in the intervention) and outcomes. Cassimatis et al highlighted in their analysis that more intensive lifestyle interventions appeared to have a greater impact on glycemic control [22]. Wu et al, who analyzed telephone interventions for type 2 diabetes, found no overall improvement in HbA_1c_ in a meta-analysis, but they did identify a significant improvement in a prespecified subgroup analysis of interventions providing intensive professional support [53]. By contrast, Inglis et al showed no significant impact on mortality of an exploratory subgroup analysis based on intensity of telemonitoring in heart failure [58].

## Discussion

### Statement of Main Findings

The individual long-term conditions considered in this metareview differed both in the quantity of evidence for telehealth interventions supporting self-management and in the findings and conclusions of the included systematic reviews. Diabetes and heart failure constituted the greatest evidence base, with available data on cancer being very limited.

The impact of telehealth-supported self-management on disease control and health care utilization was inconsistent, with positive outcomes more frequently identified in type 2 diabetes and heart failure and often no effect demonstrated in other conditions. The highest-quality evidence for heart failure showed an overall improvement in mortality in meta-analyses of telemonitoring and telephone support [58]. In contrast, none of the reviews assessing mortality in COPD showed any significant improvement with telehealth [65,66,68,69]. None of the reviews, however, reported a negative impact of interventions employing telehealth for any condition. This should be treated with some caution, however, as few reviews specifically considered or assessed for publication bias and, of those that did, some found evidence to suggest bias [38,46]. Findings varied by disease group and by components of telehealth delivery. The highest-weighted evidence showed improvement in HbA_1c_ in type 2 diabetes with interventions remotely monitoring blood glucose and in some more intensive telephone interventions [31,38,53]. Physiological telemonitoring and telephone support for heart failure were associated with reduced mortality and heart failure-specific hospital admissions [58]. For both of these conditions, however, findings were inconsistent between reviews, and analyses of similar interventions reached different conclusions [25,55,57]. Interventions for type 1 diabetes did not improve glycemic control [49]. There was some evidence for reduced hospitalization with telehealth interventions in more severe asthma and COPD [63,68], but analyses of more specific, self-management-focused interventions showed insufficient or inconsistent evidence of benefit [27,62]. The interventions described incorporated a range of self-management components. No single component was consistently effective in any disease area, although none were associated with harm. Interventions with multiple components, or more intensive interventions, may be associated with greater benefits [30,53,63,68]. In most reviews, however, the description of self-management components and analysis of their relation to clinical outcomes was not sufficiently detailed to draw firm conclusions about which components or combinations were most beneficial, or in what conditions.

### Strengths and Limitations

A strength of metareview methodology is that it allows a relatively rapid synthesis of a large body of primary literature and enables a broad overview of a subject area [15]. There are, however, limitations inherent in metareview methodology. Any systematic review is limited by the time delay from completion of a primary study to publication, and subsequently conducting the review itself. By reviewing systematic reviews, this lag time is further extended and, as such, the results risk being out of date, although we updated the review before completing the paper. The observation in 1 review that some more recent studies showed greater benefits in clinical outcomes [28] highlights the importance of up-to-date evidence in a fast-moving field such as telehealth. That we were unable to differentiate the RCT findings by year of publication is therefore a limitation of our methodology.

By relying on systematic review findings, the evidence is 1 step removed from the empirical evidence, and thus reliant on the interpretation of the review authors. This was mitigated to some extent by assessing the methodological quality of the reviews and using this to weight the evidence. This limitation is most evident, however, when addressing questions about strategies for developing and implementing telehealth interventions. For example, no reviews specifically addressed how potential participants were identified, recruited, and retained, a key issue if telehealth interventions are to be successfully implemented [73]. Few explicitly considered the importance of patient decision making in the interventions. Our consideration of the impact of the intensity of the interventions is also limited to a few systematic reviews, as the majority did not analyze the impact of this on disease control outcomes. By relying on the analysis of the review authors, we were also unable to adjust for important factors such as geographic location, age of participants, and socioeconomic variables such as educational status. A metareview such as this is thus well suited to forming an overview of the topic, but loses granularity and detail of the evidence, a limitation particularly evident when attempting to analyze components of self-management support.

There is also overlap in the included RCTs, which risks overrepresenting the results of a few RCTs included in several systematic reviews and giving a false impression of consensus. Overlap precludes meta-analysis, but the use of harvest plots provides a visual synthesis of the findings of each individual review. We designed the rules used to determine how each review was displayed in the harvest plots to ensure that the assessment of reviews was consistent and transparent. Specifying that more than 50% of RCTs in a narrative synthesis must show positive results for a review to be considered as having consistently positive findings avoids overly optimistic interpretation, but risks overlooking individual RCTs that may be particularly relevant to the review question.

There are also limitations specific to this metareview. This metareview considered only 6 long-term conditions and may have reached different conclusions had we selected a different set of conditions. However, we selected common conditions, 5 of which had a good evidence base for telehealth or self-management, or both, and included 1 area (cancer) in which these concepts are still at an early stage. The relative lack of data for cancer may reflect a lack of research in this field or simply that the available evidence has yet to be synthesized in a systematic review.

Not all the included systematic reviews explicitly focused on self-management. It could be argued that the inclusion of systematic reviews with an implied self-management focus was based on our subjective assessment. However, we developed clear rules for the inclusion criteria and undertook duplicate full-text screening, but this might have resulted in some inappropriate inclusions or exclusions due to limited description of interventions. In addition, we identified reviews that focused specifically on self-management and incorporated this into the weighting system, so as to minimize the impact of this limitation on the interpretation of the findings.

### Comparison With Other Literature

Our finding of inconsistent evidence of benefit for disease control and health care utilization with telehealth-supported self-management is similar to the findings reported by other overviews of telehealth interventions that were not specifically focused on self-management. A recent metareview of heart failure telemonitoring interventions showed a reduction in all-cause mortality (relative risk 0.60-0.85) and heart failure hospitalizations (relative risk 0.64-0.86) in an analysis of 15 meta-analyses [74]. An overview of telehealth across a number of conditions noted modest improvements in HbA_1c_ in some reviews with others noting no overall effect [2]. A consistent conclusion, reflected in our findings, is that telehealth is not associated with worse outcomes. Taken together, it appears that, while not consistently superior to usual care, telehealth is a safe alternative mode of delivery for self-management support, particularly in conditions such as heart failure and type 2 diabetes, where the evidence base is more developed. The research agenda may therefore shift to demonstrating equivalence, or understanding the impact of offering choice, or evaluating other potential benefits of telehealth (such as improved access).

While components of the reported interventions mapped to Pearce et al’s taxonomy of self-management support [14], few reviews analyzed how these related to the self-management process or clinical outcomes. In common with other overarching analyses of self-management support [12], we found no evidence to support 1 component as being consistently effective or essential for improved outcomes. The greatest volume of evidence was for educational interventions and monitoring of clinical data incorporating feedback, and several reviews, notably those with higher weighting, demonstrated improved outcomes in some (notably type 2 diabetes) but not all conditions (Figure 2). Clinical review, adherence support, and lifestyle interventions also showed some positive results, but with substantial inconsistency. There was some evidence for telehealth-delivered adherence support in asthma and diabetes, but this was variable and inconsistent. In heart failure, evidence linking knowledge, self-efficacy, or self-care behaviors to improvements in disease outcomes was either lacking or inconsistent [56]. Greenwood et al concluded that a range of components (including educational, self-monitoring, goal-driven, and interactive elements) were important in improving glycemic control [30] and emphasized the multifaceted nature of self-management support.

Analysis of the reasons for effectiveness (or lack thereof) of the interventions is limited in the included reviews. Such questions have been addressed using qualitative methodologies [75], which have highlighted that users valued convenience [76], a sense of being “watched over” [11,77], and finding the telemonitoring data reassuring [78]. Some felt empowered by these interventions to use the health care system more effectively [76,77]. Such findings correlate with the quantitative findings of this review showing that interventions with active monitoring of blood glucose data with feedback in type 2 diabetes were often associated with improved outcomes. However, qualitative explorations of some asthma and COPD interventions suggested that daily professional monitoring of telemonitored parameters (such as pulse oximetry or symptom scores) could engender reliance on professionals rather than supporting self-management [11,76,78,79]. In contrast, self-monitoring (including oximetry) without daily professional oversight gave patients greater confidence in following their self-management plans [80].

### Conclusions

While telehealth interventions were not consistently found to be superior to usual care, none of the reviews reported any negative effects, suggesting that telehealth is a safe alternative mode of delivery for self-management support, particularly in conditions such as heart failure and type 2 diabetes, where the evidence base is more developed. Improvements may be more readily seen in those with more severe disease. The decision to adopt telehealth strategies will be determined not only by clinical or demographic circumstances but also by patient or clinician preference. Findings vary within and between different conditions, and further investigation is required to establish the role in conditions (such as cancer) where current evidence is limited.

We found little explicit evidence of a mediating role for self-management in telehealth interventions and the specific components that may encourage self-care. While some evidence suggests that, in the context of type 2 diabetes, more intensive interventions may be associated with greater improvements in glycemic control, such observations are limited to specific analyses from a small number of included reviews [22,30,53], and it is not clear whether this applies to other disease areas [58]. Larger-scale trials of self-management interventions delivered by telehealth, based on explicit self-management theory [81,82], linked with process evaluations that explore intermediary outcomes such as self-efficacy, and providing detailed description of the interventions, are needed before the extent to which telehealth technologies may be harnessed to support self-management at scale can be established.

## Appendix

Multimedia Appendix 1 Search terms used in MEDLINE.

Multimedia Appendix 2 List of randomized controlled trials in included systematic reviews.

Multimedia Appendix 3 Study characteristics.

Multimedia Appendix 4 A Measurement Tool to Assess Systematic Reviews (revised) quality assessment.

Multimedia Appendix 5 Details of included studies.

Multimedia Appendix 6 Results of included systematic reviews.

## Abbreviations

COPD: chronic obstructive pulmonary disease
HbA1c: hemoglobin A1c
MeSH: Medical Subject Headings
PRISMA: Preferred Reporting Items for Systematic Reviews and Meta-analyses
PRISMS: Practical Reviews in Self-Management Support
R-AMSTAR: revised A Measurement Tool to Assess Systematic Reviews
RCT: randomized controlled trial
