# Supplementary material for: Telehealth Interventions to Support Self-Management of Long-Term Conditions: A Systematic Metareview of Diabetes, Heart Failure, Asthma, Chronic Obstructive Pulmonary Disease, and Cancer
Source: J Med Internet Res. 2017 May 17;19(5):e172. doi: 10.2196/jmir.6688 (PMC5451641; doi:10.2196/jmir.6688)
Supplement: Multimedia Appendix 1 [file jmir_v19i5e172_app1.pdf]

Additional File 1. Search terms used for Medline Search

Outline

Self management terms (grouped OR)
Long term condition terms (grouped OR)
Telemedicine terms (grouped OR)
Systematic review terms (grouped OR)
All above (grouped AND)

1. Exp Self care/
2. Exp. Communication/
3. Exp Professional Family Relations/
4. Exp Professional Patient Relations/
5. Exp Health Education/
6. Exp Attitude of health personnel/
7. Exp Patient education as topic/
8. Exp Self efficacy/
9. Exp Activities of daily living/
10. Exp Self help devices
11. Exp Community health services
12. Exp Rehabilitation
13. (Self ADJ2 (car* or manag* or help or administ* or monitor* or medicat*)) or self-car* or self-manag* or self-help or self-administ* or self-monitor* or self-medicat* or selfcar* or selfmanagement or selfhelp or selfadminist* or selfmonitor* or selfmedicat*
14. Responsab* or Automon*
15. Manag* or copes or coping
16. “Disease management”
17. “expert patient”
18. (professional or clinican) ADJ2 development
19. Educat* or train* or skill* or knowledge
20. Confidence or self-efficacy
21. (Access* or provi*) ADJ3 (information or records or results)
22. Monitor* or self-monitor* or selfmonitor*
23. ((patient or individual* or person* or client*) ADJ3 (remind* or feedback))
24. “Care plan*”
25. “Action plan*”
26. (Peer or patient or emotional or social or psychosocial) ADJ1 (support or group)
27. Adherence or compliance
28. Exercise or training or rehabilitat*
29. (Lifestyle or occupational) ADJ1 (intervention* or modification* or therapy)
30. Action plan*
31. (Reduce or remove) ADJ2 (dust or mite)
32. (Smok* or nicotine or tobacco) ADJ3 (cessation or quit*)
33. Exp. Blood Glucose Self Monitoring/
34. Foot care
35. Diet*
36. 1 or 2 or 3 or 4 or 5 or 6 or 7 or 8 or 9 or 10 or 11 or 12 or 13 or 14 or 15 or 16 or 17 or 18 or 19 or 20 or 21 or 22 or 23 or 24 or 25 or 26 or 27 or 28 or 29 or 30 or 31 or 32 or 33 or 34 or 35
37. Asthma/
38. COPD/
39. ((bronchial* or respiratory or airway* or lung*) ADJ3 (hypersensitiv* or hyperreactiv* or allerg* or insufficiency))
40. Bronch* ADJ3 (constrict* or spas*)
41. Asthma*
42. Wheez*

43. Bronchoconstrict*
44. Antiasthma*
45. Anti-asthma*
46. “Respiratory sounds”
47. “Bronchial hyperreactivity”
48. Bronchospas*
49. (Obstruct* ADJ3 (pulmonary or lung* or airway* or airflow* or bronch* or respirat*))
50. Chronic* ADJ3 bronchiti*
51. “Chronic obstructive pulmonary disease
52. “Lung disease*”
53. Emphysema*
54. COPD or CAL or COAD or COLD or COBD or AECB
55. 37 or 38 or 39 or 40 or 41 or 42 or 43 or 44 or 45 or 46 or 47 or 48 or 49 or 50 or 51 or 52 or 53 or 54
56. Diabetes mellitus, type1/
57. Diabetes mellitus, type2/
58. Insulin resistance/
59. Diabetic ketoacidosis/
60. (diabet* or dm) ADJ5 (typ* ADJ3 (one or 1 or I))
61. (diabet* or dm) ADJ5 (typ* ADJ3 (two or 2 or II))
62. (Insulin or noninsulin or non-insulin) ADJ2 (resistan* or depend*)
63. Diabet*
64. DM or DM1 or DM2 or T1D or T1DM or T2D or T2DM or NIDDM or IDDM or MODY
65. Glucose ADJ (tolerance or intolerance)
66. 56 or 57 or 58 or 59 or 60 or 61 or 62 or 63 or 64 or 65
67. Exp. Heart failure
68. Exp. Cardiovascular diseases
69. “Heart failure”
70. “Congestive heart failure”
71. “Cardiac failure”
72. “Congestive cardiac failure”
73. CHF
74. CCF
75. “Chronic heart failure”
76. “Chronic cardiac failure”
77. “Cardiovascular disease”
78. Ventricular dysfunction
79. 67 or 68 or 69 or 70 or 71 or 72 or 73 or 74 or 75 or 76 or 77 or 78
80. Exp. Cancer/
81. Exp. Neoplasms/
82. (cancer* or neoplasm* or tumo?r* or carcinoma* or sarcoma* or adenocardinoma* or adeno?carcinoma* or adenoma*)
83. 80 or 81 or 82
84. Chronic disease.tw
85. Long-term condition*.tw
86. Long term condition*.tw
87. 84 or 85 or 86
88. Exp. Telemedicine/
89. Exp. Cellular phone/
90. Exp. Text messaging/
91. (eHealth or e-Health).mp
92. (mHealth or m-health or mobile health).mp
93. (telehealth or tele-health).mp
94. (telemedicine or tele-medicine).mp
95. (telecare or telehealthcare or telehomecare or mobile telehealthcare or mobile telemedicine or mCare or m-care).mp

96. telecommunication.mp
97. (telepharmacy or tele-pharmacy).mp
98. telemonitor*.mp
99. telemanagement*.mp
100. e-compliance*.mp
101. (app* or mobile applications) adj2 (phone* or mobile* or device*).mp
102. (mobile communication or mobile technolog* or mobile devic*).mp
103. Computers/ or Microcomputers/ or Internet/ or (mobile technolog* or web*).mp
104. Cellular phone/ or Telephone/ or (cellular phone* or cell phone or mobile phone or iphone).mp
105. Text Messaging/ or (texting or text messag* or messag* or text* or short message or SMS).mp
106. (smartphone or smart-phone).mp
107. GPRS.mp
108. wireless.mp
109. bluetooth.mp
110. (remote consult*).mp
111. (Monitoring system).mp

112. 88 or 89 or 90 or 91 or 92 or 93 or 94 or 95 or 96 or 97 or 98 or 99 or 100 or 101 or 102 or 103 or 104 or 105 or 106 or 107 or 108 or 109 or 110 or 111
113. meta-analysis/
114. meta analysis as topic/
115. review literature as topic/
116. MEDLINE
117. (systematic review* or meta-analy* or metaanaly* or "research synthesis" or "literature review")
118. systematic ADJ3 literature
119. data ADJ2 extract*
120. ((information or data) ADJ3 synthesis)
121. Cochrane
122. 113 or 114 or 115 or 116 or 117 or 118 or 119 or 120 or 121
123. 55 or 66 or 79 or 83 or 87
124. 36 and 112 and 122 and 123
