# Supplementary material for: Telehealth Interventions to Support Self-Management of Long-Term Conditions: A Systematic Metareview of Diabetes, Heart Failure, Asthma, Chronic Obstructive Pulmonary Disease, and Cancer
Source: J Med Internet Res. 2017 May 17;19(5):e172. doi: 10.2196/jmir.6688 (PMC5451641; doi:10.2196/jmir.6688)
Supplement: Multimedia Appendix 2 [file jmir_v19i5e172_app2.pdf]

Diabetes RCTs

Baron 2012

Rossi, M.C., Nicolucci, A., Di Bartolo, P., Bruttomesso, D., Girelli, A., Ampudia, F.J., Kerr, D., Ceriello, A., Mayor, C.D.L.Q., Pellegrini, F. and Horwitz, D., 2010. Diabetes Interactive Diary: A New Telemedicine System Enabling Flexible Diet and Insulin Therapy While Improving Quality of Life An open-label, international, multicenter, randomized study. *Diabetes Care*, *33*(1), pp.109-115.

Farmer, A.J., Gibson, O.J., Dudley, C., Bryden, K., Hayton, P.M., Tarassenko, L. and Neil, A., 2005. A randomized controlled trial of the effect of real-time telemedicine support on glycemic control in young adults with type 1 diabetes (ISRCTN 46889446). *Diabetes care*, *28*(11), pp.2697-2702.

Cho, J.H., Lee, H.C., Lim, D.J., Kwon, H.S. and Yoon, K.H., 2009. Mobile communication using a mobile phone with a glucometer for glucose control in Type 2 patients with diabetes: as effective as an Internet-based glucose monitoring system. *Journal of Telemedicine and Telecare*, *15*(2), pp.77-82.

Faridi, Z., Liberti, L., Shuval, K., Northrup, V., Ali, A. and Katz, D.L., 2008. Evaluating the impact of mobile telephone technology on type 2 diabetic patients' self‐management: the NICHE pilot study. *Journal of evaluation in clinical practice*, *14*(3), pp.465-469.

Kim, H.S., 2007. A randomized controlled trial of a nurse short-message service by cellular phone for people with diabetes. *International journal of nursing studies*, *44*(5), pp.687-692.

Kim, H.S. and Jeong, H.S., 2007. A nurse short message service by cellular phone in type‐2 diabetic patients for six months. *Journal of clinical nursing*, *16*(6), pp.1082-1087.

Hee‐Sung, K., 2007. Impact of web‐based nurse's education on glycosylated haemoglobin in type 2 diabetic patients. *Journal of clinical nursing*, *16*(7), pp.1361-1366.

Yoon, K.H. and Kim, H.S., 2008. A short message service by cellular phone in type 2 diabetic patients for 12 months. *Diabetes research and clinical practice*, *79*(2), pp.256-261.

Istepanian, R.S., Zitouni, K., Harry, D., Moutosammy, N., Sungoor, A., Tang, B. and Earle, K.A., 2009. Evaluation of a mobile phone telemonitoring system for glycaemic control in patients with diabetes. *Journal of Telemedicine and Telecare*, *15*(3), pp.125-128.

Kim HS, Song MS. Technological intervention for obese patients with type 2 diabetes. Appl Nurs Res. 2008;21(2):84–9.

Kim, S.I. and Kim, H.S., 2008. Effectiveness of mobile and internet intervention in patients with obese type 2 diabetes. *International journal of medical informatics*, *77*(6), pp.399-404.

Quinn, C.C., Clough, S.S., Minor, J.M., Lender, D., Okafor, M.C. and Gruber-Baldini, A., 2008. WellDoc™ mobile diabetes management randomized controlled trial: change in clinical and behavioral outcomes and patient and physician satisfaction. *Diabetes technology & therapeutics*, *10*(3), pp.160-168.

Rodríguez-Idígoras, M.I., Sepúlveda-Muñoz, J., Sánchez-Garrido-Escudero, R., Martínez-González, J.L., Escolar-Castelló, J.L., Paniagua-Gómez, I.M., Bernal-López, R., Fuentes-Simón, M.V. and Garófano-Serrano, D., 2009. Telemedicine influence on the follow-up of type 2 diabetes patients. *Diabetes technology & therapeutics*, *11*(7), pp.431-437.

Kim, C.S., Park, S.Y., Kang, J.G., Lee, S.J., Ihm, S.H., Choi, M.G. and Yoo, H.J., 2010. Insulin dose titration system in diabetes patients using a short messaging service automatically produced by a knowledge matrix. *Diabetes technology & therapeutics*, *12*(8), pp.663-669.

Yoo, H.J., Park, M.S., Kim, T.N., Yang, S.J., Cho, G.J., Hwang, T.G., Baik, S.H., Choi, D.S., Park, G.H. and Choi, K.M., 2009. A ubiquitous chronic disease care system using cellular phones and the internet. *Diabetic Medicine*, *26*(6), pp.628-635.

Quinn, C.C., Shardell, M.D., Terrin, M.L., Barr, E.A., Ballew, S.H. and Gruber-Baldini, A.L., 2011. Cluster-randomized trial of a mobile phone personalized behavioral intervention for blood glucose control. *Diabetes care*, *34*(9), pp.1934-1942.

Beatty 2013

Glasgow, R.E., Boles, S.M., Mckay, H.G., Feil, E.G. and Barrera, M., 2003. The D-Net diabetes self-management program: long-term implementation, outcomes, and generalization results. *Preventive medicine*, *36*(4), pp.410-419.

Glasgow, R.E., Nutting, P.A., Toobert, D.J., King, D.K., Strycker, L.A., Jex, M., O'neill, C., Whitesides, H. and Merenich, J., 2006. Effects of a brief computer-assisted diabetes self-management intervention on dietary, biological and quality-of-life outcomes. *Chronic Illness*, *2*(1), pp.27-38.

Lorig, K., Ritter, P.L., Laurent, D.D., Plant, K., Green, M., Jernigan, V.B.B. and Case, S., 2010. Online diabetes self-management program A randomized study. *Diabetes care*, *33*(6), pp.1275-1281.

McKay, H.G., Glasgow, R.E., Feil, E.G., Boles, S.M. and Barrera Jr, M., 2002. Internet-based diabetes self-management and support: Initial outcomes from the Diabetes Network project. *Rehabilitation Psychology*, *47*(1), p.31.

McKay, H.G., King, D., Eakin, E.G., Seeley, J.R. and Glasgow, R.E., 2001. The diabetes network internet-based physical activity intervention a randomized pilot study. *Diabetes care*, *24*(8), pp.1328-1334.

Beraterechea 2014

Wojcicki, J.M., Ladyzynski, P., Krzymien, J., Jozwicka, E., Blachowicz, J., Janczewska, E., Czajkowski, K. and Karnafel, W., 2001. What we can really expect from telemedicine in intensive diabetes treatment: results from 3-year study on type 1 pregnant diabetic women. *Diabetes technology & therapeutics*, *3*(4), pp.581-589.

Shetty, A.S., Chamukuttan, S., Nanditha, A., Raj, R.K. and Ramachandran, A., 2011. Reinforcement of adherence to prescription recommendations in Asian Indian diabetes patients using short message service (SMS)–a pilot study. *J Assoc Physicians India*, *59*(11), pp.711-4.

Balsa, A.I. and Gandelman, N., 2010. The impact of ICT on health promotion: A randomized experiment with diabetic patients.

Cassimatis 2012

Anderson DR, Christison-Lagay J, Villagra V, Liu H, Dziura J. Managing the space between visits: a randomized trial of disease management for diabetes in a community health center. J Gen Intern Med 2010; 25 :1116 – 22

Frosch DL, Uy V, Ochoa S, Mangione CM. Evaluation of a behavior support intervention for patients with poorly controlled diabetes. Arch Intern Med 2011; 171 :2011 – 17

Maljanian R, Grey N, Staff I, Conroy L. Intensive telephone follow-up to a hospital-based disease management model for patients with diabetes mellitus. Dis Manag 2005; 8 :15 – 25

Piette JD, Weinberger M, Kraemer FB, McPhee SJ. Impact of automated calls with nurse follow-up on diabetes treatment outcomes in a Department of Veterans Affairs health care system: a randomized controlled trial. Diabetes Care 2001; 24 :202 – 08

Piette JD, Weinberger M, McPhee SJ. The effect of automated calls with telephone nurse follow-up on patient-centered outcomes of diabetes care: a randomized, controlled trial. Med Care 2000; 38 : 218 – 30

Sigurdardottir AK, Benediktsson R, Jonsdottir H. Instruments to tailor care of people with type 2 diabetes. J Adv Nurs 2009; 65 :2118 – 30

Wolever RQ, Dreusicke M, Fikkan J, et al. Integrative health coaching for patients with type 2 diabetes: a randomized clinical trial. Diabetes Educ 2010; 36 :629 – 39

Kim H, Oh J. Adherence to diabetes control recommendations: impact of nurse telephone calls. J Adv Nurs 2003; 44 :256 – 61

Nesari M, Zakerimoghadam M, Rajab A, Bassampour S, Faghihzadeh S. Effect of telephone follow-up on adherence to a diabetes therapeutic regime. Jpn J Nurs Sci 2010; 7 :121 – 28

Bell AM, Fonda SJ, Walker MS, Schmidt V, Vigersky RA. Mobile phone-based video messages for diabetes self-care support. J Diabetes Sci Technol. 2012; 6 :310 – 19

Piette JD. Satisfaction with automated telephone disease management calls and its relationship to

their use. Diabetes Educ 2000; 26 :1003 – 10

Walker EA, Shmukler C, Ullman R, Blanco E, Scollan-Koliopoulus M, Cohen HW. Results of a successful telephonic intervention to improve diabetes control in urban adults: a randomized trial. Diabetes Care 2011; 34 :2 – 7

Trief P, Sandberg JG, Ploutz-Snyder R, et al. Promoting couples collaboration in type 2 diabetes: the diabetes support project pilot data. Fam Syst Health 2011; 29 :253 – 61

Sacco WP, Malone JI, Morrison AD, Friedman A, Wells K. Effect of a brief, regular telephone intervention by paraprofessionals for type 2 diabetes. J Behav Med 2009; 32 :349 – 359

Currell 2000

Ahring K, Joyce C, Ahring J, Farid N. Telephone modem access improves diabetes control in those with insulin- requiring diabetes. Diabetes Care 1992; 15 (8):971–975.

Marrero D, Vandagriff J, Kronz K, Fineberg N, Golden M, Gray D, et al. Using telecommunication technology to manage children with diabetes: the computer-linked outpatient clinic (CLOC) study. The Diabetes Educator 1995; 21 (4):313–319

De Jongh 2012

Franklin V, Waller A, Pagliari C, Greene S. “Sweet Talk”: text messaging support for intensive insulin therapy for young people with diabetes. Diabetes Technology and Therapeutics 2003; 5 (6):991–6.

Franklin VL, Greene A, Waller A, Greene SA, Pagliari C. Patients’ engagement with “Sweet Talk” - a text messaging support system for young people with diabetes. Journal of Medical Internet Research 2008; 10 (2):e20.

Franklin VL, Waller A, Pagliari C, Greene SA. A randomized controlled trial of Sweet Talk, a text-messaging system to support young people with diabetes. Diabetic Medicine 2006; 23 (12):1332–8

Waller A, Franklin V, Pagliari C, Greene S. Participatory design of a text message scheduling system to support young people with diabetes. Health Informatics Journal 2006; 12 (4):304–18.

Hanauer DA, Wentzell K, Laffel N, Laffel LM. Computerized Automated Reminder Diabetes System (CARDS): E-Mail and SMS cell phone text messaging reminders to support diabetes management. Diabetes Technology and Therapeutics2009;11(2):99–106

Farmer 2005

Marrero D, Vandagriff J, Kronz K, Fineberg N, Golden M, Gray D, et al. Using telecommunication technology to manage children with diabetes: the computer-linked outpatient clinic (CLOC) study. The Diabetes Educator 1995; 21 (4):313–319

Chase HP, Pearson JA, Wightman C, Roberts MD, Oderberg AD, Garg SK. Modem transmission of glucose values reduces the costs and need for clinic visits. Diabetes Care 2003; 26 : 1475.

Welch G, Sokolove M, Mullin C, Master P, Horton P. Use of a modem- equipped blood glucose meter augmented with biweekly educator support lowers HbA1c in type 1 diabetes. Diabetes 2003; 52 : A100.

Ahring KK, Joyce C, Ahring JPK, Farid NR. Telephone modem access improves diabetes control in those with insulin-requiring diabetes. Diabetes Care 1992; 15: 971–975

Montori VM, Helgemoe PK, Guyatt GH, Dean DS, Leung TW, Smith SA et al. Telecare for patients with type 1 diabetes and inadequate glycemic control: a randomized controlled trial and meta-analysis. Diabetes Care 2004; 27: 1088–1094

Horan PP, Yarborough MC, Besigel G, Carlson DR. Computer-assistedself-control of diabetes by adolescents. Diabetes Educ1990; 16: 205 – 211

Biermann E, Dietrich W, Standl E. Telecare of diabetic patients withintensified insulin therapy. A randomized clinical trial. Studies Health Technol Informatics 2000; 77: 327–332.

Biermann E, Dietrich W, Rihl J, Standl E. Are there time and cost savings by using telemanagement for

patients on intensified insulin therapy? A randomised controlled trial. Comput Meth Programs Biomed 2002; 69: 137–146

Piette JD, Weinberger M, McPhee SJ, Mah CA, Kraemer FB, Crapo LM. Do automated calls with nurse follow-up improve self-care and glycemic control among vulnerable patients with diabetes? Am J Med 2000; 108: 20–27.

Piette JD, Weinberger M, Kraemer FB, McPhee SJ. Impact of automated calls with nurse follow-up on diabetes treatment outcomes in a department of veterans affairs health care system: a randomized controlled trial. Diabetes Care 2001; 24: 202.

Kruger DF, White K, Galpern A, Mann K, Massiro A, McLellan M et al. Effect of modem transmission of blood glucose data on telephone consultation time, clinic work flow and patient satisfaction for patients with gestational diabetes mellitus. J Am Acad Nurs Practitioners 2003; 15: 375.

Ladyzynski P, Wojcicki JM, Krzymien J, Blachowicz J, Jozwicka E, Czajkowski K et al.

Teletransmission system supporting intensive insulin treatment of out-clinic type 1 diabetic pregnant women. Technical assessment during 3 years' application. Int J Artif Organs 2001; 24: 157–163.

Wojcicki JM, Ladyzynski P, Krzymien J, Jozwicka E, Blachowicz J, Janczewska E et al.

What we can really expect from telemedicine in intensive diabetes treatment: results from 3-year study on type 1 pregnant diabetic women. Diabetes Technol Ther 2001; 3: 581–589.

Di Biase N, Napoli A, Sabbatini A, Borrello E, Buongiorno AM, Fallucca F. Telemedicine in the treatment of diabetic pregnancy. AnnIst Super Sanita 1997; 33: 347–351

Fallucca F, Di Biase N, Sabbatini A, Borrello E, Sciullo E, Napoli A. Telemedicine in the treatment of diabetic pregnancy. Pract Diabetes Int 1996; 13: 115–118.

Farmer 2016

Vervloet M, Linn AJ, van Weert JCM, de Bakker DH, Bouvy ML, van Dijk L. The effectiveness of interventions using electronic reminders to improve adherence to chronic medication: a systematic review of the literature. J Am Med Inform Assoc 2012; 19: 696–704.

Arora S, Peters AL, Burner E, Lam CN, Menchine M. Trial to examine text message-based mHealth in emergency department patients with diabetes (TExT-MED): a randomized controlled trial. Ann Emerg Med 2014; 63: 745–754.

Brath H, Morak J, Kastenbauer T, Modre-Osprian R, Strohner- Kastenbauer H, Schwarz M et al. Mobile health (mHealth) basedmedication adherence measurement: a pilot trial using electronic blisters in diabetes patients. Br J Clin Pharmacol 2013; 76: 47–55.

Shetty AS, Chamukuttan S, Nanditha A, Raj RK, Ramachandran A. Reinforcement of adherence to prescription recommendations in Asian Indian diabetes patients using short message service (SMS): a pilot study. J Assoc Physicians India 2011; 59: 711–714.

Bogner HR, Morales KH, de Vries HF, Cappola AR. Integrated management of type 2 diabetes mellitus and depression treatment to improve medication adherence: a randomized controlled trial. Ann Fam Med 2012; 10: 15–22.

Guldberg TL, Vedsted P, Kristensen JK, Lauritzen T. Improved quality of Type 2 diabetes care following electronic feedback of treatment status to general practitioners: a cluster randomized controlled trial. Diabet Med 2011; 28: 325–332.

Nesari M, Zakerimoghadam M, Rajab A, Bassampour S, Faghihzadeh S. Effect of telephone follow-up on adherence to a diabetes therapeutic regimen. Jpn J Nurs Sci 2010; 7: 121–128.

Odegard PS, Goo A, Hummel J, Williams KL, Gray SL. Caring for poorly controlled diabetes mellitus: a randomized pharmacist intervention. Ann Pharmacother 2005; 39: 433–440.

Vervloet M, Dijk L, Santen-Reestman J, Vlijmen B, Wingerden P, Bouvy ML et al. SMS reminders improve adherence to oral medication in type 2 diabetes patients who are real time electronically monitored. Int J Med Inform 2012; 81: 594–604.

Vervloet M, van Dijk L, de Bakker DH, Souverein PC, Santen-Reestman J, van Vlijmen B et al. Short- and long-term effects of real-time medication monitoring with short message service (SMS) reminders for missed doses on the refill adherence of people with Type 2 diabetes: evidence from a randomized controlled trial. Diabet Med 2014; 31: 821–828.

Vervloet M, van Dijk L, Santen-Reestman J, van Vlijmen B, Bouvy ML, de Bakker DH. Improving medication adherence in diabetes type 2 patients through Real Time Medication Monitoring: a Randomised Controlled Trial to evaluate the effect of monitoring patients' medication use combined with short message service (SMS) reminders. BMC Health Serv Res 2011; 11: 5.

Wakefield BJ, Holman JE, Ray A, Scherubel M, Adams MR, Hillis SL et al. Effectiveness of home telehealth in comorbid diabetes and hypertension: a randomized, controlled trial. Telemed J E Health 2011; 17: 254–261.

Fisher L, Hessler D, Glasgow RE, Arean PA, Masharani U, Naranjo D et al. REDEEM: a pragmatic trial to reduce diabetes distress. Diabetes Care 2013; 36: 2551–2558.

Glasgow R, Christiansen SM, Kurz D, King DK, Woolley T, Faber AJ et al. Engagement in a diabetes self-management website: usage patterns and generalizability of program use. J Med Internet Res 2011; 13: e9.

Glasgow RE, Kurz D, King D, Dickman JM, Faber AJ, Halterman E et al. Outcomes of minimal and moderate support versions of an internet-based diabetes self-management support program. J Gen Intern Med 2010; 25: 1315–1322.

Glasgow RE, Kurz D, King D, Dickman JM, Faber AJ, Halterman E et al. Twelve-month outcomes of an Internet-based diabetes selfmanagement support program. Patient Educ Couns 2012; 87: 81–92.

Flodgren 2015

Ahring K, Joyce C, Ahring J, Farid N. Telephone modem access improves diabetes control in those with insulin- requiring diabetes. Diabetes Care 1992; 15 (8):971–5

Biermann E, Dietrich W, Rihl J, Standl E. Are there time and cost savings by using telemanagement for patients on intensified insulin therapy? A randomised, controlled trial. Computer Methods and Programs in Biomedicine 2002; 69 (2): 137–46.

Biermann E, Dietrich W, Standl E. Telecare of diabetic patients with intensified insulin therapy. A randomized clinical trial. Studies in Health Technology Information 2000; 77 :327–32

Boaz M, Hellman K, Wainstein J. An automated telemedicine system improves patient-reported well-being. Diabetes Technology and Therapy 2009; 11 (3):181–6.

Bond GE, Burr R, Wolf FM, Price M, McCurry SM, Teri L. The effects of a web-based Intervention on the physical outcomes associated with diabetes among adults age 60 and older: a randomized trial. Diabetes Technology and Therapy 2007; 9 (1):52–9. [DOI: 10.1089/dia.2006.0057]

Bond GE, Burr RL, Wolf FM, Feldt K. The effects of a web-based intervention on psychosocial well-being among adults aged 60 and older with diabetes: a randomized trial. Diabetes Educator 2010; 36 (3):446–56

Charpentier, G., Benhamou, P.Y., Dardari, D., Clergeot, A., Franc, S., Schaepelynck-Belicar, P., Catargi, B., Melki, V., Chaillous, L., Farret, A. and Bosson, J.L., 2011. The diabeo software enabling individualized insulin dose adjustments combined with telemedicine support improves hba1c in poorly controlled type 1 diabetic patients. *Diabetes care*, *34*(3), pp.533-539.;

Chase, H.P., Pearson, J.A., Wightman, C., Roberts, M.D., Oderberg, A.D. and Garg, S.K., 2003. Modem transmission of glucose values reduces the costs and need for clinic visits. *Diabetes Care*, *26*(5), pp.1475-1479.

Davis R, Hitch A, Salaam M, Nichols M, Moran R, Mayer-Davis EJ. The diabetes TeleCare (DTC) Study: 24- month follow-up data on patients living in rural medically underserved areas. Conference Abstract. Conference: 69th Annual Meeting of the American Diabetes Association New Orleans, LA United States. 2009, issue var.pagings.

Davis R, Mayer-Davis EJ. Cost effectiveness of a telehealth- based diabetes self-management (DSME) intervention in a rural community. Diabetes 2011; Vol. Conference: 71st Scientific Sessions of the American Diabetes Association San Diego, CA United States. Conference Start: 20110624 Conference End: 20110628. Conference Publication:, issue var.pagings:60–A326.

Davis RM, Fowler S, Bellis K, Pockl J, Al Pakalnis V, Woldorf A. Telemedicine improves eye examination rates in individuals with diabetes: a model for eye-care delivery in underserved communities. Investigative Ophtalmology and Visual Science 2003; 26 (8):2476.

Davis RM, Hitch AD, Salaam MM, Herman WH, Zimmer-Galler IE, Mayer-Davis EJ. TeleHealth improves diabetes self-management in an underserved community. Diabetes Care 2010; 33 :1712-7.

Davis RM, Pockl J, Bellis K. Improved diabetic eye care utilizing telemedicine: A randomized controlled trial. Investigative Ophthalmology and Visual Science 2003; 44 :E- Abstract 166

Izquierdo, R.E., Knudson, P.E., Meyer, S., Kearns, J., Ploutz-Snyder, R. and Weinstock, R.S., 2003. A comparison of diabetes education administered through telemedicine versus in person. *Diabetes care*, *26*(4), pp.1002-1007.

Izquierdo, R., Morin, P.C., Bratt, K., Moreau, Z., Meyer, S., Ploutz-Snyder, R., Wade, M. and Weinstock, R.S., 2009. School-centered telemedicine for children with type 1 diabetes mellitus. *The Journal of pediatrics*, *155*(3), pp.374-379.

Jansà, M., Vidal, M., Viaplana, J., Levy, I., Conget, I., Gomis, R. and Esmatjes, E., 2006. Telecare in a structured therapeutic education programme addressed to patients with type 1 diabetes and poor metabolic control. *Diabetes research and clinical practice*, *74*(1), pp.26-32.

Kim, H.S., 2007. A randomized controlled trial of a nurse short-message service by cellular phone for people with diabetes. *International journal of nursing studies*, *44*(5), pp.687-692.

Kim, H.S. and Jeong, H.S., 2007. A nurse short message service by cellular phone in type‐2 diabetic patients for six months. *Journal of clinical nursing*, *16*(6), pp.1082-1087.

Kwon, H.S., Cho, J.H., Kim, H.S., Song, B.R., Ko, S.H., Lee, J.M., Kim, S.R., Chang, S.A., Kim, H.S., Cha, B.Y. and Lee, K.W., 2004. Establishment of blood glucose monitoring system using the internet. *Diabetes care*, *27*(2), pp.478-483.

Marrero, D.G., Vandagriff, J.L., Kronz, K., Fineberg, N.S., Golden, M.P., Msn, D.G., Orr, D.P., Wright, J.C. and Johnson, N.B., 1995. Using telecommunication technology to manage children with diabetes: the Computer-Linked Outpatient Clinic (CLOC) Study. *The Diabetes Educator*, *21*(4), pp.313-319.

McCarrier, K.P., Ralston, J.D., Hirsch, I.B., Lewis, G., Martin, D.P., Zimmerman, F.J. and Goldberg, H.I., 2009. Web-based collaborative care for type 1 diabetes: a pilot randomized trial. *Diabetes technology & therapeutics*, *11*(4), pp.211-217.

McMahon, G.T., Gomes, H.E., Hohne, S.H., Hu, T.M.J., Levine, B.A. and Conlin, P.R., 2005. Web-based care management in patients with poorly controlled diabetes. *Diabetes care*, *28*(7), pp.1624-1629.

Ralston, J.D., Hirsch, I.B., Hoath, J., Mullen, M., Cheadle, A. and Goldberg, H.I., 2009. Web-based collaborative care for type 2 diabetes a pilot randomized trial. *Diabetes care*, *32*(2), pp.234-239.

Rodríguez-Idígoras, M.I., Sepúlveda-Muñoz, J., Sánchez-Garrido-Escudero, R., Martínez-González, J.L., Escolar-Castelló, J.L., Paniagua-Gómez, I.M., Bernal-López, R., Fuentes-Simón, M.V. and Garófano-Serrano, D., 2009. Telemedicine influence on the follow-up of type 2 diabetes patients. *Diabetes technology & therapeutics*, *11*(7), pp.431-437.

Demiris G, Speedie S, Finkelstein S, Harris I. Communication patterns and technical quality of virtual visits in home care. Journal of Telemedicine and Telecare 2003; 9 (4):210–5.

Izquierdo R, Lagua CT, Meyer S, Ploutz-Snyder RJ, Palmas W, Eimicke JP, et al. Telemedicine intervention effects on waist circumference and body mass index in the IDEATel project. Diabetes Technology and Therapy 2010; 12 (3): 213–20.

Moreno L, Dale SB, Chen AY, Magee CA. Costs to Medicare of the Informatics for Diabetes Education andTelemedicine (IDEATel) home telemedicine demonstration findings from an independent evaluation. Diabetes Care 2009; 32 (7):1202–4.

Palmas W, Shea S, Starren J, Teresi JA, Ganz ML, Burton TM, et al. Medicare payments, healthcare service use, and telemedicine implementation costs in a randomized trial comparing telemedicine case management with usual care in medically underserved participants with diabetes mellitus (IDEATel). Journal of American Medical Informatics Association 2010; 17 (2):196–202.

Shea S, Consortium IDEATel. The Informatics for Diabetes and Education Telemedicine (IDEATel) project. Transaction of the American Clinical and Climatological Association 2007; 118 :289–304.

Shea S, Kothari D, Teresi J, Jian K, Eimicke J, Lantigua R, et al. Social impact analysis of the effects of a telemedicine intervention to improve diabetes outcomes in an ethnically diverse, medically underserved population: findings from the IDEATel Study. American Journal of Public Health 2013; 103 (5):e1–7.

Shea S, Weinstock RS, Starren J, Teresi J, Palmas W, Field L, et al. A randomized trial comparing telemedicine case management with usual care in older, ethnically diverse, medically underserved patients with diabetes mellitus. Journal of the American Medical Informatics Association 2006; 13 (1):40–51.

Shea S, Weinstock RS, Teresi JA, Palmas W, Starren J, Cimino JJ, et al. A randomized trial comparing telemedicine case management with usual care in older, ethnically diverse, medically underserved patients with diabetes mellitus: 5 year results of the IDEATel study. Journal of the American Medical Informativcs Association 2009; 16 (4):446–56.

Trief PM, Izquierdo R, Eimicke JP, Teresi JA, Goland R, Palmas W, et al. Adherence to diabetes self care for white,African-American and Hispanic American telemedicine participants: 5 year results from the IDEATel project. Ethnicity & Health 2013; 18 (1):83–96.

Trief PM, Morin PC, Izquierdo R, Teresi JA, Eimicke JP, Goland R, et al. Depression and glycemic control in elderly ethnically diverse patients with diabetes. Diabetes Care 2006; 29:830–5.

Trief PM, Teresi JA, Izquierdo R, Morin PC, Goland R, Field L, et al. Psychosocial outcomes of telemedicine case management for elderly patients with diabetes: the randomized IDEATel trial. Diabetes Care 2007; 30 (5): 1266–8.

Weinstock RS, Teresi JA, Goland R, Izquierdo R, Palmas W,Eimicke JP, et al. Glycemic control and health disparities in older ethnically diverse underserved adults with diabetes: five-year results from the Informatics for Diabetes Education and Telemedicine (IDEATel) study.Diabetes Care 2011; 34 (2):274–9

Stone, R.A., Rao, R.H., Sevick, M.A., Cheng, C., Hough, L.J., Macpherson, D.S., Franko, C.M., Anglin, R.A., Obrosky, D.S. and DeRubertis, F.R., 2010. Active care management supported by home telemonitoring in veterans with type 2 diabetes. *Diabetes care*, *33*(3), pp.478-484.

Whitlock WL, Brown A, Moore K, Pavliscsak H, Dingbaum A, Lacefield D, et al. Telemedicine improved diabetic management. Military Medicine 2000;165(8):579–84

Ładyżyński, P. and Wójcicki, J.M., 2007. Home telecare during intensive insulin treatment–metabolic control does not improve as much as expected. *Journal of telemedicine and telecare*, *13*(1), pp.44-47.

Wojcicki, J.M., Ladyzynski, P., Krzymien, J., Jozwicka, E., Blachowicz, J., Janczewska, E., Czajkowski, K. and Karnafel, W., 2001. What we can really expect from telemedicine in intensive diabetes treatment: results from 3-year study on type 1 pregnant diabetic women. *Diabetes technology & therapeutics*, *3*(4), pp.581-589.

Garzia-Lizana 2007

Meigs, J.B., Cagliero, E., Dubey, A., Murphy-Sheehy, P., Gildesgame, C., Chueh, H., Barry, M.J., Singer, D.E. and Nathan, D.M., 2003. A controlled trial of web-based diabetes disease management. *Diabetes Care*, *26*(3), pp.750-757.

Biermann, E., Dietrich, W., Rihl, J. and Standl, E., 2002. Are there time and cost savings by using telemanagement for patients on intensified insulin therapy?: A randomised, controlled trial. *Computer methods and programs in biomedicine*, *69*(2), pp.137-146.

Chase, H.P., Pearson, J.A., Wightman, C., Roberts, M.D., Oderberg, A.D. and Garg, S.K., 2003. Modem transmission of glucose values reduces the costs and need for clinic visits. *Diabetes Care*, *26*(5), pp.1475-1479.

Izquierdo, R.E., Knudson, P.E., Meyer, S., Kearns, J., Ploutz-Snyder, R. and Weinstock, R.S., 2003. A comparison of diabetes education administered through telemedicine versus in person. *Diabetes care*, *26*(4), pp.1002-1007.

Marrero, D.G., Vandagriff, J.L., Kronz, K., Fineberg, N.S., Golden, M.P., Msn, D.G., Orr, D.P., Wright, J.C. and Johnson, N.B., 1995. Using telecommunication technology to manage children with diabetes: the Computer-Linked Outpatient Clinic (CLOC) Study. *The Diabetes Educator*, *21*(4), pp.313-319.

Kwon, H.S., Cho, J.H., Kim, H.S., Song, B.R., Ko, S.H., Lee, J.M., Kim, S.R., Chang, S.A., Kim, H.S., Cha, B.Y. and Lee, K.W., 2004. Establishment of blood glucose monitoring system using the internet. *Diabetes care*, *27*(2), pp.478-483.

McKay, H.G., King, D., Eakin, E.G., Seeley, J.R. and Glasgow, R.E., 2001. The diabetes network internet-based physical activity intervention. *Diabetes care*, *24*(8), pp.1328-1334.

Greenwood 2014

Shea, S., Weinstock, R.S., Starren, J., Teresi, J., Palmas, W., Field, L., Morin, P., Goland, R., Izquierdo, R.E., Wolff, L.T. and Ashraf, M., 2006. A randomized trial comparing telemedicine case management with usual care in older, ethnically diverse, medically underserved patients with diabetes mellitus. *Journal of the American Medical Informatics Association*, *13*(1), pp.40-51.

Shea, S., Weinstock, R.S., Teresi, J.A., Palmas, W., Starren, J., Cimino, J.J., Lai, A.M., Field, L., Morin, P.C., Goland, R. and Izquierdo, R.E., 2009. A randomized trial comparing telemedicine case management with usual care in older, ethnically diverse, medically underserved patients with diabetes mellitus: 5 year results of the IDEATel study. *Journal of the American Medical Informatics Association*, *16*(4), pp.446-456.

Carter EL, Nunlee-Bland G, Callender C. A patient-centric, provider-assisted diabetes telehealth self-management intervention for urban minorities.

Perspect Health Inf Manag. 2011;8:1

Faridi, Z., Liberti, L., Shuval, K., Northrup, V., Ali, A. and Katz, D.L., 2008. Evaluating the impact of mobile telephone technology on type 2 diabetic patients' self-management: the NICHE pilot study. *Journal of evaluation in clinical practice*, *14*(3), pp.465-469

Cho, J.H., Lee, H.C., Lim, D.J., Kwon, H.S. and Yoon, K.H., 2009. Mobile communication using a mobile phone with a glucometer for glucose control in Type 2 patients with diabetes: as effective as an Internet-based glucose monitoring system. *Journal of Telemedicine and Telecare*, *15*(2), pp.77-82.

Wakefield, B.J., Holman, J.E., Ray, A., Scherubel, M., Adams, M.R., Hillis, S.L. and Rosenthal, G.E., 2011. Effectiveness of home telehealth in comorbid diabetes and hypertension: a randomized, controlled trial. *Telemedicine and e-Health*, *17*(4), pp.254-261.

Prato, S.D., Nicolucci, A., Lovagnini-Scher, A.C., Turco, S., Leotta, S. and Vespasiani, G., 2012. Telecare provides comparable efficacy to conventional self-monitored blood glucose in patients with type 2 diabetes titrating one injection of insulin glulisine—the ELEONOR study. *Diabetes technology & therapeutics*, *14*(2), pp.175-182.

Kim, H.S., Kim, N.C. and Ahn, S.H., 2006. Impact of a nurse short message service intervention for patients with diabetes. *Journal of nursing care quality*, *21*(3), pp.266-271.

Stone, R.A., Rao, R.H., Sevick, M.A., Cheng, C., Hough, L.J., Macpherson, D.S., Franko, C.M., Anglin, R.A., Obrosky, D.S. and DeRubertis, F.R., 2010. Active care management supported by home telemonitoring in veterans with type 2 diabetes. *Diabetes care*, *33*(3), pp.478-484.

Tang, P.C., Overhage, J.M., Chan, A.S., Brown, N.L., Aghighi, B., Entwistle, M.P., Hui, S.L., Hyde, S.M., Klieman, L.H., Mitchell, C.J. and Perkins, A.J., 2013. Online disease management of diabetes: engaging and motivating patients online with enhanced resources-diabetes (EMPOWER-D), a randomized controlled trial. *Journal of the American Medical Informatics Association*, *20*(3), pp.526-534.

Quinn, C.C., Clough, S.S., Minor, J.M., Lender, D., Okafor, M.C. and Gruber-Baldini, A., 2008. WellDoc™ mobile diabetes management randomized controlled trial: change in clinical and behavioral outcomes and patient and physician satisfaction. *Diabetes technology & therapeutics*, *10*(3), pp.160-168.

Quinn, C.C., Shardell, M.D., Terrin, M.L., Barr, E.A., Ballew, S.H. and Gruber-Baldini, A.L., 2011. Cluster-randomized trial of a mobile phone personalized behavioral intervention for blood glucose control. *Diabetes care*, *34*(9), pp.1934-1942.

Lim, S., Kang, S.M., Shin, H., Lee, H.J., Yoon, J.W., Yu, S.H., Kim, S.Y., Yoo, S.Y., Jung, H.S., Park, K.S. and Ryu, J.O., 2011. Improved glycemic control without hypoglycemia in elderly diabetic patients using the ubiquitous healthcare service, a new medical information system. *Diabetes care*, *34*(2), pp.308-313.

McMahon, G.T., Gomes, H.E., Hohne, S.H., Hu, T.M.J., Levine, B.A. and Conlin, P.R., 2005. Web-based care management in patients with poorly controlled diabetes. *Diabetes care*, *28*(7), pp.1624-1629.

Kim HS. A randomized controlled trial of a nurse short-message service by cellular phone for people with diabetes. Int J Nurs Stud. 2007;44(5):687-692

Bujnowska-Fedak, M.M., Puchała, E. and Steciwko, A., 2011. The impact of telehome care on health status and quality of life among patients with diabetes in a primary care setting in Poland. *Telemedicine and e-Health*, *17*(3), pp.153-163.

Rodríguez-Idígoras, M.I., Sepúlveda-Muñoz, J., Sánchez-Garrido-Escudero, R., Martínez-González, J.L., Escolar-Castelló, J.L., Paniagua-Gómez, I.M., Bernal-López, R., Fuentes-Simón, M.V. and Garófano-Serrano, D., 2009. Telemedicine influence on the follow-up of type 2 diabetes patients. *Diabetes technology & therapeutics*, *11*(7), pp.431-437.

Hamine 2015

Nglazi MD, Bekker LG, Wood R, Hussey GD, Wiysonge CS. Mobile phone text messaging for promotingadherence to anti-tuberculosis treatment: a systematic review protocol. Syst Rev. 2013;2(1):6. doi: 10.1186/2046-4053-2-6

Lim, S., Kang, S.M., Shin, H., Lee, H.J., Yoon, J.W., Yu, S.H., Kim, S.Y., Yoo, S.Y., Jung, H.S., Park, K.S. and Ryu, J.O., 2011. Improved glycemic control without hypoglycemia

in elderly diabetic patients using the ubiquitous healthcare service, a new medical information system. *Diabetes care*, *34*(2), pp.308-313.

Carroll, A.E., DiMeglio, L.A., Stein, S. and Marrero, D.G., 2011. Using a cell phone–based glucose monitoring system for adolescent diabetes management. *The Diabetes Educator*, *37*(1), pp.59-66.

Strandbygaard, U., Thomsen, S.F. and Backer, V., 2010. A daily SMS reminder increases adherence to asthma treatment: a three-month follow-up study. *Respiratory medicine*, *104*(2), pp.166-171.

Lv, Y., Zhao, H., Liang, Z., Dong, H., Liu, L., Zhang, D. and Cai, S., 2012. A mobile phone short message service improves perceived control of asthma: a randomized controlled trial. *Telemedicine and e-Health*, *18*(6), pp.420-426.

Vervloet, M., van Dijk, L., Santen-Reestman, J., Van Vlijmen, B., Van Wingerden, P., Bouvy, M.L. and de Bakker, D.H., 2012. SMS reminders improve adherence to oral medication in type 2 diabetes patients who are real time electronically monitored. *International journal of medical informatics*, *81*(9), pp.594-604.

Franklin, V.L., Waller, A., Pagliari, C. and Greene, S.A., 2006. A randomized controlled trial of Sweet Talk, a text-messaging system to support young people with diabetes. *Diabetic Medicine*, *23*(12), pp.1332-1338.

Hanauer, D.A., Wentzell, K., Laffel, N. and Laffel, L.M., 2009. Computerized Automated Reminder Diabetes System (CARDS): e-mail and SMS cell phone text messaging reminders to support diabetes management. *Diabetes technology & therapeutics*, *11*(2), pp.99-106.

Kumar, V.S., Wentzell, K.J., Mikkelsen, T., Pentland, A. and Laffel, L.M., 2004. The DAILY (Daily Automated Intensive Log for Youth) trial: a wireless, portable system to improve adherence and glycemic control in youth with diabetes. *Diabetes technology & therapeutics*, *6*(4), pp.445-453.

Khonsari, S., Subramanian, P., Chinna, K., Latif, L.A., Ling, L.W. and Gholami, O., 2015. Effect of a reminder system using an automated short message service on medication

adherence following acute coronary syndrome. *European Journal of Cardiovascular Nursing*, *14*(2), pp.170-179.

Ostojic, V., Cvoriscec, B., Ostojic, S.B., Reznikoff, D., Stipic-Markovic, A. and Tudjman, Z., 2005. Improving asthma control through telemedicine: a study of short-message service. *Telemedicine Journal & E-Health*, *11*(1), pp.28-35.

Liu, W.T., Wang, C.H., Lin, H.C., Lin, S.M., Lee, K.Y., Lo, Y.L., Hung, S.H., Chang, Y.M., Chung, K.F. and Kuo, H.P., 2008. Efficacy of a cell phone-based exercise programme for COPD. *European Respiratory Journal*, *32*(3), pp.651-659.

Benhamou, P.Y., Melki, V., Boizel, R., Perreal, F., Quesada, J.L., Bessieres-Lacombe, S., Bosson, J.L., Halimi, S. and Hanaire, H., 2007. One-year efficacy and safety of Web-based follow-up using cellular phone in type 1 diabetic patients under insulin pump therapy: the PumpNet study. *Diabetes & metabolism*, *33*(3), pp.220-226.

Cho, J.H., Lee, H.C., Lim, D.J., Kwon, H.S. and Yoon, K.H., 2009. Mobile communication using a mobile phone with a glucometer for glucose control in Type 2 patients with diabetes: as effective as an Internet-based glucose monitoring system. *Journal of Telemedicine and Telecare*, *15*(2), pp.77-82.

DeSalvo, D.J., Keith-Hynes, P., Peyser, T., Place, J., Caswell, K., Wilson, D.M., Harris, B., Clinton, P., Kovatchev, B. and Buckingham, B.A., 2014. Remote glucose monitoring in camp setting reduces the risk of prolonged nocturnal hypoglycemia. *Diabetes technology & therapeutics*, *16*(1), pp.1-7.

Kim, C., Kim, H., Nam, J., Cho, M., Park, J., Kang, E., Ahn, C., Cha, B., Lee, E., Lim, S. and Kim, K., 2007. Internet diabetic patient management using a short messaging service automatically produced by a knowledge matrix system. *Diabetes Care*, *30*(11), pp.2857-2858.

Kirwan, M., Vandelanotte, C., Fenning, A. and Duncan, M.J., 2013. Diabetes self-management smartphone application for adults with type 1 diabetes: randomized controlled trial. *Journal of medical Internet research*, *15*(11), p.e235.

Shetty, A.S., Chamukuttan, S., Nanditha, A., Raj, R.K. and Ramachandran, A., 2011.

Reinforcement of adherence to prescription recommendations in Asian Indian diabetes patients using short message service (SMS)–a pilot study. *J Assoc Physicians India*, *59*(11), pp.711-4.

Zolfaghari, M., Mousavifar, S.A., Pedram, S. and Haghani, H., 2012. The impact of nurse short message services and telephone follow-ups on diabetic adherence: which one is more effective?. *Journal of clinical nursing*, *21*(13-14), pp.1922-1931.

Quinn, C.C., Shardell, M.D., Terrin, M.L., Barr, E.A., Ballew, S.H. and Gruber-Baldini, A.L., 2011. Cluster-randomized trial of a mobile phone personalized behavioral intervention for blood glucose control. *Diabetes care*, *34*(9), pp.1934-1942.

Kim, H.S. and Jeong, H.S., 2007. A nurse short message service by cellular phone in type-2 diabetic patients for six months. *Journal of clinical nursing*, *16*(6), pp.1082-1087.

Kiselev, A.R., Gridnev, V.I., Shvartz, V.A., Posnenkova, O.M. and Dovgalevsky, P.Y., 2012. Active ambulatory care management supported by short message services and mobile phone technology in patients with arterial hypertension. *Journal of the American Society of Hypertension*, *6*(5), pp.346-355.

Bell, A.M., Fonda, S.J., Walker, M.S., Schmidt, V. and Vigersky, R.A., 2012. Mobile phone-based video messages for diabetes self-care support. *Journal of diabetes science and technology*, *6*(2), pp.310-319.

Blasco, A., Carmona, M., Fernández-Lozano, I., Salvador, C.H., Pascual, M., Sagredo, P.G., Somolinos, R., Muñoz, A., García-López, F., Escudier, J.M. and Mingo, S., 2012. Evaluation of a telemedicine service for the secondary prevention of coronary artery disease. *Journal of cardiopulmonary rehabilitation and prevention*, *32*(1), pp.25-31.

Leu, M.G., Norris, T.E., Hummel, J., Isaac, M. and Brogan, M.W., 2005. A randomized, controlled trial of an automated wireless messaging system for diabetes. *Diabetes technology & therapeutics*, *7*(5), pp.710-718.

Nagrebetsky, A., Larsen, M., Craven, A., Turner, J., McRobert, N., Murray, E., Gibson, O., Neil, A., Tarassenko, L. and Farmer, A., 2013. Stepwise self-titration of oral

glucose-lowering medication using a mobile telephone-based telehealth platform in type 2 diabetes: a feasibility trial in primary care. *Journal of diabetes science and technology*, *7*(1), pp.123-134.

Quinn, C.C., Clough, S.S., Minor, J.M., Lender, D., Okafor, M.C. and Gruber-Baldini, A., 2008. WellDoc™ mobile diabetes management randomized controlled trial: change in clinical and behavioral outcomes and patient and physician satisfaction. *Diabetes technology & therapeutics*, *10*(3), pp.160-168.

Rossi, M.C., Nicolucci, A., Di Bartolo, P., Bruttomesso, D., Girelli, A., Ampudia, F.J., Kerr, D., Ceriello, A., Mayor, C.D.L.Q., Pellegrini, F. and Horwitz, D., 2010. Diabetes interactive diary: a new telemedicine system enabling flexible diet and insulin therapy while improving quality of life. *Diabetes Care*, *33*(1), pp.109-115.

Hotz 2012

Franklin, V.L., Waller, A., Pagliari, C. and Greene, S.A., 2006. A randomized controlled trial of Sweet Talk, a text-messaging system to support young people with diabetes. *Diabetic Medicine*, *23*(12), pp.1332-1338.

Istepanian, R.S., Zitouni, K., Harry, D., Moutosammy, N., Sungoor, A., Tang, B. and Earle, K.A., 2009. Evaluation of a mobile phone telemonitoring system for glycaemic control in patients with diabetes. *Journal of Telemedicine and Telecare*, *15*(3), pp.125-128.

Quinn, C.C., Clough, S.S., Minor, J.M., Lender, D., Okafor, M.C. and Gruber-Baldini, A., 2008. WellDoc™ mobile diabetes management randomized controlled trial: change in clinical and behavioral outcomes and patient and physician satisfaction. *Diabetes technology & therapeutics*, *10*(3), pp.160-168.

Benhamou, P.Y., Melki, V., Boizel, R., Perreal, F., Quesada, J.L., Bessieres-Lacombe, S., Bosson, J.L., Halimi, S. and Hanaire, H., 2007. One-year efficacy and safety of Web-based follow-up using cellular phone in type 1 diabetic patients under insulin pump therapy: the PumpNet study. *Diabetes & metabolism*, *33*(3), pp.220-226.

Farmer, A., Gibson, O., Hayton, P., Bryden, K., Dudley, C., Neil, A. and Tarassenko, L., 2005. A real-time, mobile phone-based telemedicine system to support young adults with type 1 diabetes. *Journal of Innovation in Health Informatics*, *13*(3), pp.171-177.

Faridi, Z., Liberti, L., Shuval, K., Northrup, V., Ali, A. and Katz, D.L., 2008. Evaluating the impact of mobile telephone technology on type 2 diabetic patients' self-management: the NICHE pilot study. *Journal of evaluation in clinical practice*, *14*(3), pp.465-469.

Wangberg, S.C., Årsand, E. and Andersson, N., 2006. Diabetes education via mobile text messaging. *Journal of telemedicine and telecare*, *12*(1_suppl), pp.55-56.

Hanauer, D.A., Wentzell, K., Laffel, N. and Laffel, L.M., 2009. Computerized Automated Reminder Diabetes System (CARDS): e-mail and SMS cell phone text messaging reminders to support diabetes management. *Diabetes technology & therapeutics*, *11*(2), pp.99-106.

Rami, B., Popow, C., Horn, W., Waldhoer, T. and Schober, E., 2006. Telemedical support to improve glycemic control in adolescents with type 1 diabetes mellitus. *European journal of pediatrics*, *165*(10), pp.701-705.

Tasker, A.P., Gibson, L., Franklin, V., Gregor, P. and Greene, S., 2007. What is the frequency of symptomatic mild hypoglycemia in type 1 diabetes in the young?: assessment by novel mobile phone technology and computer-based interviewing. *Pediatric diabetes*, *8*(1), pp.15-20.

Huang 2015

Cho JH, Kwon HS, Kim HS, Oh JA & Yoon KH. Effects on diabetes management of a health-care provider mediated, remote coaching system via a PDA-type glucometer and the internet. Journal of Telemedicine and Telecare 2011 17 365–370.

Graziano JA & Gross CR. A randomized controlled trial of an automated telephone intervention to improve glycemic control in type 2 diabetes. ANS. Advances in Nursing Science 2009 32 E42–E57.

Kim CS, Park SY, Kang JG, Lee SJ, Ihm SH, Choi MG & Yoo HJ. Insulin dose titration systemindiabetespatientsusinga shortmessaging service automatically produced by a knowledge matrix. Diabetes Technology & Therapeutics 2010 12 663–669.

Lim S, Kang SM, Shin H, Lee HJ, Won YJ, Yu SH, Kim SY, Yoo SY, Jung HS, Park KS et al . Improved glycemic control without hypo- glycemia in elderly diabetic patients using the ubiquitous healthcare service, a new medical information system. Diabetes Care 2011 34 308–313.

Nesari M, Zakerimoghadam M, Rajab A, Bassampour S & Faghihzadeh S. Effect of telephone follow-up on adherence to a diabetes therapeutic regimen. Japan Journal of Nursing Science 2010 7 121–128.

Oh JA, Kim HS, Yoon KH & Choi ES. A telephone-delivered intervention to improve glycemic control in type 2 diabetic patients. Yonsei Medical Journal 2003 44 1–8.

Quinn CC, Shardell MD, Terrin ML, Barr EA, Ballew SH & Gruber- Baldini AL. Cluster-randomized trial of a mobile phone personalized behavioral intervention for blood glucose control. Diabetes Care 2011 34 1934–1942.

Stone RA, Rao RH, Sevick MA, Cheng C, Hough LJ, Macpherson DS, Franko CM, Anglin RA, Obrosky DS & Derubertis FR. Active care management supported by home telemonitoring in veterans with type 2 diabetes: the DiaTel randomized controlled trial. Diabetes Care 2010 33 478–484.

Yoo HJ, Park MS, Kim TN, Yang SJ, Cho GJ, Hwang TG, Baik SH, Choi DS, Park GH & Choi KM. A ubiquitous chronic disease care system using cellular phones and the internet. Diabetic Medicine 2009 26 628–635.

Tang PC, Overhage JM, Chan AS, Brown NL, Aghighi B, Entwistle MP, Hui SL, Hyde SM, Klieman LH, Mitchell CJ et al . Online disease management of diabetes: engaging and motivating patients online with enhanced resources-diabetes (EMPOWER-D), a randomized controlled trial. Journal of the American Medical Informatics Association 2013 20 526–534.

Piette JD, Weinberger M, McPhee SJ, Mah CA, Kraemer FB & Crapo LM. Do automated calls with nurse follow-up improve self-care and glycemic control among vulnerable patients with diabetes? American Journal of Medicine 2000 108 20–27.

Rodriguez-Idigoras MI, Sepulveda-Munoz J, Sanchez-Garrido-Escudero R, Martinez-Gonzalez JL, Escolar-Castello JL, Paniagua-Gomez IM, Bernal- Lopez R, Fuentes-Simon MV & Garofano-Serrano D. Telemedicine influence on the follow-up of type 2 diabetes patients. Diabetes Technology & Therapeutics 2009 11 431–437.

Shea S, Weinstock RS, Teresi JA, Palmas W, Starren J, Cimino JJ, Lai AM, Field L, Morin PC, Goland R et al . A randomized trial comparing telemedicine case management with usual care in older, ethnically diverse, medically underserved patients with diabetes mellitus: 5 year results of the IDEATel study. Journal of the American Medical Informatics Association 2009 16 446–456.

Song MS & Kim HS. Intensive management program to improve glycosylated hemoglobin levels and adherence to diet in patients with type 2 diabetes. Applied Nursing Research 2009 22 42–47.

Yoon KH & Kim HS. A short message service by cellular phone in type 2 diabetic patients for 12 months. Diabetes Research and Clinical Practice 2008 79 256–261.

Bujnowska-Fedak MM, Puchała E & Steciwko A. The impact of telehome care on health status and quality of life among patients with diabetes in a primary care setting in Poland. Telemedicine Journal and E-Health : The Official Journal of the American Telemedicine Association201117153–163.

Cho JH, Chang SA, Kwon HS, Choi YH, Ko SH, Moon SD, Yoo SJ, Song KH, Son HS, Kim HS et al. Long-term effect of the internet-based glucose monitoring system on HbA1c reduction and glucose stability: a 30-month follow-up study for diabetes management with a ubiquitous medical care system.Diabetes Care2006292625–2631

Kim CJ & Kang DH. Utility of a web-based intervention for individuals with type 2 diabetes: the impact on physical activity levels and glycemic control. Computers, Informatics, Nursing200624337–345.

Jaana 2007

Biermann, E., Dietrich, W., Rihl, J. and Standl, E., 2002. Are there time and cost savings by using telemanagement for patients on intensified insulin therapy?: A randomised, controlled trial. *Computer methods and programs in biomedicine*, *69*(2), pp.137-146.

Gómez, E.J., Hernando, M.E., Garcıa, A., Del Pozo, F., Cermeño, J., Corcoy, R., Brugués, E. and De Leiva, A., 2002. Telemedicine as a tool for intensive management of diabetes: the DIABTel experience. *Computer methods and programs in biomedicine*, *69*(2), pp.163-177.

Montori, V.M., Helgemoe, P.K., Guyatt, G.H., Dean, D.S., Leung, T.W., Smith, S.A. and Kudva, Y.C., 2004. Telecare for patients with type 1 diabetes and inadequate glycemic control. *Diabetes care*, *27*(5), pp.1088-1094.

Ahring, K.K., Ahring, J.P.K., Joyce, C. and Farid, N.R., 1992. Telephone modem access improves diabetes control in those with insulin-requiring diabetes. *Diabetes care*, *15*(8), pp.971-975.

Marrero, D.G., Vandagriff, J.L., Kronz, K., Fineberg, N.S., Golden, M.P., Msn, D.G., Orr, D.P., Wright, J.C. and Johnson, N.B., 1995. Using telecommunication technology to manage children with diabetes: the Computer-Linked Outpatient Clinic (CLOC) Study. *The Diabetes Educator*, *21*(4), pp.313-319.

Edmonds, M., Bauer, M., Osborn, S., Lutfiyya, H., Mahon, J., Doig, G., Grundy, P., Gittens, C., Molenkamp, G. and Fenlon, D., 1998. Using the Vista 350 telephone to communicate the results of home monitoring of diabetes mellitus to a central database and to provide feedback. *International journal of medical informatics*, *51*(2), pp.117-125.

Piette, J.D., Weinberger, M., McPhee, S.J., Mah, C.A., Kraemer, F.B. and Crapo, L.M., 2000. Do automated calls with nurse follow-up improve self-care and glycemic control among vulnerable patients with diabetes?. *The American journal of medicine*, *108*(1), pp.20-27.

Chase, H.P., Pearson, J.A., Wightman, C., Roberts, M.D., Oderberg, A.D. and Garg, S.K., 2003. Modem transmission of glucose values reduces the costs and need for clinic visits. *Diabetes Care*, *26*(5), pp.1475-1479.

Lavery, L.A., Higgins, K.R., Lanctot, D.R., Constantinides, G.P., Zamorano, R.G., Armstrong, D.G., Athanasiou, K.A. and Agrawal, C.M., 2004. Home monitoring of foot skin temperatures to prevent ulceration. *Diabetes care*, *27*(11), pp.2642-2647.

Billiard, A., Rohmer, V., Roques, M.A., Joseph, M.G., Suraniti, S., Giraud, P., Limal, J.M., Fressinaud, P. and Marre, M., 1991. Telematic transmission of computerized blood glucose profiles for IDDM patients. *Diabetes care*, *14*(2), pp.130-134.

Shultz, E.K., Bauman, A., Hayward, M. and Holzman, R., 1992. Improved Care of Patients with Diabetes through Telecommunications. *Annals of the New York Academy of Sciences*, *670*(1), pp.141-145.

Tsang, M.W., Mok, M., Kam, G., Jung, M., Tang, A. and Chan, U., 2001. Improvement in diabetes control with a monitoring system based on a hand-held, touch-screen electronic diary. *Journal of telemedicine and telecare*, *7*(1), pp.47-50.

Vähätalo, M.A., Virtamo, H.E., Viikari, J.S. and Rönnemaa, T., 2004. Cellular phone transferred self blood glucose monitoring: prerequisites for positive outcome. *Practical Diabetes*, *21*(5), pp.192-194.

Kok 2011

Bond, G.E., Burr, R., Wolf, F.M., Price, M., McCurry, S.M. and Teri, L., 2007. The effects of a web-based intervention on the physical outcomes associated with diabetes among adults age 60 and older: a randomized trial. *Diabetes technology & therapeutics*, *9*(1), pp.52-59.

Cho, J.H., Chang, S.A., Kwon, H.S., Choi, Y.H., Ko, S.H., Moon, S.D., Yoo, S.J., Song, K.H., Son, H.S., Kim, H.S. and Lee, W.C., 2006. Long-term effect of the Internet-based glucose monitoring system on HbA1c reduction and glucose stability. *Diabetes Care*, *29*(12), pp.2625-2631.

Homko, C.J., Santamore, W.P., Whiteman, V., Bower, M., Berger, P., Geifman-Holtzman, O. and Bove, A.A., 2007. Use of an internet-based telemedicine system to manage underserved women with gestational diabetes mellitus. *Diabetes technology & therapeutics*, *9*(3), pp.297-306.

Kim, S.I. and Kim, H.S., 2008. Effectiveness of mobile and internet intervention in patients with obese type 2 diabetes. *International journal of medical informatics*, *77*(6), pp.399-404.

Ralston, J.D., Hirsch, I.B., Hoath, J., Mullen, M., Cheadle, A. and Goldberg, H.I., 2009. Web-based collaborative care for type 2 diabetes a pilot randomized trial. *Diabetes care*, *32*(2), pp.234-239.

Hee-Sung, K., 2007. Impact of web-based nurse's education on glycosylated haemoglobin in type 2 diabetic patients. *Journal of clinical nursing*, *16*(7), pp.1361-1366.

Kwon, H.S., Cho, J.H., Kim, H.S., Song, B.R., Ko, S.H., Lee, J.M., Kim, S.R., Chang, S.A., Kim, H.S., Cha, B.Y. and Lee, K.W., 2004. Establishment of blood glucose monitoring system using the internet. *Diabetes care*, *27*(2), pp.478-483.

Shea, S., Weinstock, R.S., Starren, J., Teresi, J., Palmas, W., Field, L., Morin, P., Goland, R., Izquierdo, R.E., Wolff, L.T. and Ashraf, M., 2006. A randomized trial comparing telemedicine case management with usual care in older, ethnically diverse, medically underserved patients with diabetes mellitus. *Journal of the American Medical Informatics Association*, *13*(1), pp.40-51.

Yoon, K.H. and Kim, H.S., 2008. A short message service by cellular phone in type 2 diabetic patients for 12 months. *Diabetes research and clinical practice*, *79*(2), pp.256-261.

Krishna 2008

Benhamou, P.Y., Melki, V., Boizel, R., Perreal, F., Quesada, J.L., Bessieres-Lacombe, S., Bosson, J.L., Halimi, S. and Hanaire, H., 2007. One-year efficacy and safety of Web-based follow-up using cellular phone in type 1 diabetic patients under insulin pump therapy: the PumpNet study. *Diabetes & metabolism*, *33*(3), pp.220-226.

Franklin, V.L., Waller, A., Pagliari, C. and Greene, S.A., 2006. A randomized controlled trial of Sweet Talk, a text-messaging system to support young people with diabetes. *Diabetic Medicine*, *23*(12), pp.1332-1338.

Kim, H.S. and Jeong, H.S., 2007. A nurse short message service by cellular phone in type‐2 diabetic patients for six months. *Journal of clinical nursing*, *16*(6), pp.1082-1087.

Kim, H.S., 2007. A randomized controlled trial of a nurse short-message service by cellular phone for people with diabetes. *International journal of nursing studies*, *44*(5), pp.687-692.

Kim, S.I. and Kim, H.S., 2008. Effectiveness of mobile and internet intervention in patients with obese type 2 diabetes. *International journal of medical informatics*, *77*(6), pp.399-404.

Kim, H.S. and Song, M.S., 2008. Technological intervention for obese patients with type 2 diabetes. *Applied Nursing Research*, *21*(2), pp.84-89.

Rami, B., Popow, C., Horn, W., Waldhoer, T. and Schober, E., 2006. Telemedical support to improve glycemic control in adolescents with type 1 diabetes mellitus. *European journal of pediatrics*, *165*(10), pp.701-705.

Tasker, A.P., Gibson, L., Franklin, V., Gregor, P. and Greene, S., 2007. What is the frequency of symptomatic mild hypoglycemia in type 1 diabetes in the young?: assessment by novel mobile phone technology and computer-based interviewing. *Pediatric diabetes*, *8*(1), pp.15-20.

Yoon, K.H. and Kim, H.S., 2008. A short message service by cellular phone in type 2 diabetic patients for 12 months. *Diabetes research and clinical practice*, *79*(2), pp.256-261.

Krishna 2009

Benhamou, P.Y., Melki, V., Boizel, R., Perreal, F., Quesada, J.L., Bessieres-Lacombe, S., Bosson, J.L., Halimi, S. and Hanaire, H., 2007. One-year efficacy and safety of Web-based follow-up using cellular phone in type 1 diabetic patients under insulin pump therapy: the PumpNet study. *Diabetes & metabolism*, *33*(3), pp.220-226.

Franklin, V.L., Waller, A., Pagliari, C. and Greene, S.A., 2006. A randomized controlled trial of Sweet Talk, a text-messaging system to support young people with diabetes. *Diabetic Medicine*, *23*(12), pp.1332-1338.

Kim, H.S. and Jeong, H.S., 2007. A nurse short message service by cellular phone in type‐2 diabetic patients for six months. *Journal of clinical nursing*, *16*(6), pp.1082-1087.

Kim, H.S., 2007. A randomized controlled trial of a nurse short-message service by cellular phone for people with diabetes. *International journal of nursing studies*, *44*(5), pp.687-692.

Kim, S.I. and Kim, H.S., 2008. Effectiveness of mobile and internet intervention in patients with obese type 2 diabetes. *International journal of medical informatics*, *77*(6), pp.399-404.

Kim, H.S. and Song, M.S., 2008. Technological intervention for obese patients with type 2 diabetes. *Applied Nursing Research*, *21*(2), pp.84-89.

Rami, B., Popow, C., Horn, W., Waldhoer, T. and Schober, E., 2006. Telemedical support to improve glycemic control in adolescents with type 1 diabetes mellitus. *European journal of pediatrics*, *165*(10), pp.701-705.

Tasker, A.P., Gibson, L., Franklin, V., Gregor, P. and Greene, S., 2007. What is the frequency of symptomatic mild hypoglycemia in type 1 diabetes in the young?: assessment by novel mobile phone technology and computer-based interviewing. *Pediatric diabetes*, *8*(1), pp.15-20.

Yoon, K.H. and Kim, H.S., 2008. A short message service by cellular phone in type 2 diabetic patients for 12 months. *Diabetes research and clinical practice*, *79*(2), pp.256-261.

Kuijpers 2012

Bond, G.E., Burr, R.L., Wolf, F.M. and Feldt, K., 2010. The effects of a web-based intervention on psychosocial well-being among adults aged 60 and older with diabetes a randomized trial. *The diabetes educator*, *36*(3), pp.446-456.

Glasgow, R.E., Boles, S.M., Mckay, H.G., Feil, E.G. and Barrera, M., 2003. The D-Net diabetes self-management program: long-term implementation, outcomes, and generalization results. *Preventive medicine*, *36*(4), pp.410-419.

Glasgow, R.E., Kurz, D., King, D., Dickman, J.M., Faber, A.J., Halterman, E., Wooley, T., Toobert, D.J., Strycker, L.A., Estabrooks, P.A. and Osuna, D., 2010. Outcomes of minimal and moderate support versions of an internet-based diabetes self-management support program. *Journal of general internal medicine*, *25*(12), pp.1315-1322.

Glasgow, R.E., Kurz, D., King, D., Dickman, J.M., Faber, A.J., Halterman, E., Woolley, T., Toobert, D.J., Strycker, L.A., Estabrooks, P.A. and Osuna, D., 2012. Twelve-month outcomes of an Internet-based diabetes self-management support program. *Patient education and counseling*, *87*(1), pp.81-92.

Kim, C.J. and Kang, D.H., 2006. Utility of a Web-based intervention for individuals with type 2 diabetes: the impact on physical activity levels and glycemic control. *CIN: Computers, Informatics, Nursing*, *24*(6), pp.337-345.

Liebreich, T., Plotnikoff, R.C., Courneya, K.S. and Boulé, N., 2009. Diabetes NetPLAY: A physical activity website and linked email counselling randomized intervention for individuals with type 2 diabetes. *International Journal of Behavioral Nutrition and Physical Activity*, *6*(1), p.18.

Lorig, K., Ritter, P.L., Laurent, D.D., Plant, K., Green, M., Jernigan, V.B.B. and Case, S., 2010. Online diabetes self-management program. *Diabetes care*, *33*(6), pp.1275-1281.

Lorig, K.R., Ritter, P.L., Laurent, D.D. and Plant, K., 2006. Internet-based chronic disease self-management: a randomized trial. *Medical care*, *44*(11), pp.964-971.

McKay, H.G., King, D., Eakin, E.G., Seeley, J.R. and Glasgow, R.E., 2001. The diabetes network internet-based physical activity intervention. *Diabetes care*, *24*(8), pp.1328-1334.

Richardson CR, Mehari KS, McIntyre LG, Janney AW, Fortlage LA, Sen A, Strecher VJ, Piette JD. A randomized trial comparing structured and lifestyle goals in an internet-mediated walking program forpeople with type 2 diabetes. Int J Behav Nutr Phys Act. 2007;4:59

Trief, P.M., Teresi, J.A., Izquierdo, R., Morin, P.C., Goland, R., Field, L., Eimicke, J.P., Brittain, R., Starren, J., Shea, S. and Weinstock, R.S., 2007. Psychosocial outcomes of telemedicine case management for elderly patients with diabetes the randomized IDEATel trial. *Diabetes Care*, *30*(5), pp.1266-1268.

Wangberg, S.C., 2008. An Internet-based diabetes self-care intervention tailored to self-efficacy. *Health Education Research*, *23*(1), pp.170-179.

Liang 2011

Yoon, K.H. and Kim, H.S., 2008. A short message service by cellular phone in type 2 diabetic patients for 12 months. *Diabetes research and clinical practice*, *79*(2), pp.256-261.

Yoo, H.J., Park, M.S., Kim, T.N., Yang, S.J., Cho, G.J., Hwang, T.G., Baik, S.H., Choi, D.S., Park, G.H. and Choi, K.M., 2009. A ubiquitous chronic disease care system using cellular phones and the internet. *Diabetic Medicine*, *26*(6), pp.628-635.

Hanauer, D.A., Wentzell, K., Laffel, N. and Laffel, L.M., 2009. Computerized Automated Reminder Diabetes System (CARDS): e-mail and SMS cell phone text messaging reminders to support diabetes management. *Diabetes technology & therapeutics*, *11*(2), pp.99-106.

Faridi, Z., Liberti, L., Shuval, K., Northrup, V., Ali, A. and Katz, D.L., 2008. Evaluating the impact of mobile telephone technology on type 2 diabetic patients' self-management: the NICHE pilot study. *Journal of evaluation in clinical practice*, *14*(3), pp.465-469.

Istepanian, R.S., Zitouni, K., Harry, D., Moutosammy, N., Sungoor, A., Tang, B. and Earle, K.A., 2009. Evaluation of a mobile phone telemonitoring system for glycaemic control in patients with diabetes. *Journal of Telemedicine and Telecare*, *15*(3), pp.125-128.

Cho, J.H., Lee, H.C., Lim, D.J., Kwon, H.S. and Yoon, K.H., 2009. Mobile communication using a mobile phone with a glucometer for glucose control in Type 2 patients with diabetes: as effective as an Internet-based glucose monitoring system. *Journal of Telemedicine and Telecare*, *15*(2), pp.77-82.

Liu, C.T., Yeh, Y.T., Lee, T.I. and Li, Y.C., 2005. Observations on online services for diabetes management. *Diabetes Care*, *28*(11), pp.2807a-2808.

Quinn, C.C., Clough, S.S., Minor, J.M., Lender, D., Okafor, M.C. and Gruber-Baldini, A., 2008. WellDoc™ mobile diabetes management randomized controlled trial: change in clinical and behavioral outcomes and patient and physician satisfaction. *Diabetes technology & therapeutics*, *10*(3), pp.160-168.

Franklin, V.L., Waller, A., Pagliari, C. and Greene, S.A., 2006. A randomized controlled trial of Sweet Talk, a text-messaging system to support young people with diabetes. *Diabetic Medicine*, *23*(12), pp.1332-1338.

Kim C, Kang J, Lee S, Hong E, Ihm S, Kim D et al. Insulin dose titration system in diabetic patients using a short messaging service automatically produced by a knowledge matrix. Diabetologia 2009;52(Suppl1): S405

Rossi, M.C., Nicolucci, A., Di Bartolo, P., Bruttomesso, D., Girelli, A., Ampudia, F.J., Kerr, D., Ceriello, A., Mayor, C.D.L.Q., Pellegrini, F. and Horwitz, D., 2010. Diabetes interactive diary: a new telemedicine system enabling flexible diet and insulin therapy while improving quality of life. *Diabetes Care*, *33*(1), pp.109-115.

Lieber 2014

Polonsky WH, Fisher L, Schikman CH, Hinnen DA, Parkin CG, Jelsovsky Z, Petersen B, Schweitzer M, Wagner RS. Structured self-monitoring of blood glucose significantly reduces A1C levels in poorly controlled, noninsulin-treated type 2 diabetes: results from the Structured Testing Program study. Diabetes Care. 2011 Feb;34(2):262-7.

Bosi, E., Scavini, M., Ceriello, A., Cucinotta, D., Tiengo, A., Marino, R., Bonizzoni, E. and Giorgino, F., 2013. Intensive Structured Self-Monitoring of Blood Glucose and Glycemic Control in Noninsulin-Treated Type 2 Diabetes. *Diabetes care*, *36*(10), pp.2887-2894.

Lim S, Kang SM, Shin H, Lee HJ, Won Yoon J, Yu SH, Kim SY, Yoo SY, Jung HS, Park KS, Ryu JO, Jang HC. Improved glycemic control without hypoglycemia in

elderly diabetic patients using the ubiquitous healthcare service, a new medical information system. Diabetes Care. 2011 Feb;34(2):308-13

McKee MD, Fletcher J, Sigal I, Giftos J, Schechter C, Walker EA. A collaborative approach to control hypertension in diabetes: outcomes of a pilot intervention. J Prim Care Community Health. 2011 Jul 1;2(3):148-52

Chase HP, Beck R, Tamborlane W, Buckingham B, Mauras N, Tsalikian E, Wysocki T, Weinzimer S, Kollman C, Ruedy K, Xing D. A randomized multicenter trial comparing the GlucoWatch Biographer with standard glucose monitoring in children with type 1 diabetes. Diabetes Care. 2005 May;28(5):1101-6.

Marcolino 2013

Wakefield, B.J., Holman, J.E., Ray, A., Scherubel, M., Adams, M.R., Hillis, S.L. and Rosenthal, G.E., 2011. Effectiveness of home telehealth in comorbid diabetes and hypertension: a randomized, controlled trial. *Telemedicine and e-Health*, *17*(4), pp.254-261.

Bujnowska-Fedak, M.M., Puchała, E. and Steciwko, A., 2011. The impact of telehome care on health status and quality of life among patients with diabetes in a primary care setting in Poland. *Telemedicine and e-Health*, *17*(3), pp.153-163.

Shea, S., Weinstock, R.S., Starren, J., Teresi, J., Palmas, W., Field, L., Morin, P., Goland, R., Izquierdo, R.E., Wolff, L.T. and Ashraf, M., 2006. A randomized trial comparing telemedicine case management with usual care in older, ethnically diverse, medically underserved patients with diabetes mellitus. *Journal of the American Medical Informatics Association*, *13*(1), pp.40-51.

Rodríguez-Idígoras, M.I., Sepúlveda-Muñoz, J., Sánchez-Garrido-Escudero, R., Martínez-González, J.L., Escolar-Castelló, J.L., Paniagua-Gómez, I.M., Bernal-López, R., Fuentes-Simón, M.V. and Garófano-Serrano, D., 2009. Telemedicine influence on the follow-up of type 2 diabetes patients. *Diabetes technology & therapeutics*, *11*(7), pp.431-437.

Izquierdo, R., Lagua, C.T., Meyer, S., Ploutz-Snyder, R.J., Palmas, W., Eimicke, J.P., Kong, J., Teresi, J.A., Shea, S. and Weinstock, R.S., 2010. Telemedicine intervention effects on waist circumference and body mass index in the IDEATel project. *Diabetes technology & therapeutics*, *12*(3), pp.213-220.

Stone, R.A., Rao, R.H., Sevick, M.A., Cheng, C., Hough, L.J., Macpherson, D.S., Franko, C.M., Anglin, R.A., Obrosky, D.S. and DeRubertis, F.R., 2010. Active care management supported by home telemonitoring in veterans with type 2 diabetes. *Diabetes care*, *33*(3), pp.478-484.

Ralston, J.D., Hirsch, I.B., Hoath, J., Mullen, M., Cheadle, A. and Goldberg, H.I., 2009. Web-based collaborative care for type 2 diabetes a pilot randomized trial. *Diabetes care*, *32*(2), pp.234-239.

Bond, G.E., Burr, R., Wolf, F.M., Price, M., McCurry, S.M. and Teri, L., 2007. The effects of a web-based intervention on the physical outcomes associated with diabetes among adults age 60 and older: a randomized trial. *Diabetes technology & therapeutics*, *9*(1), pp.52-59.

Piette, J.D., Weinberger, M., McPhee, S.J., Mah, C.A., Kraemer, F.B. and Crapo, L.M., 2000. Do automated calls with nurse follow-up improve self-care and glycemic control among vulnerable patients with diabetes?. *The American journal of medicine*, *108*(1), pp.20-27.

Piette, J.D., Weinberger, M., Kraemer, F.B. and McPhee, S.J., 2001. Impact of automated calls with nurse follow-up on diabetes treatment outcomes in a department of veterans affairs health care system. *Diabetes care*, *24*(2), pp.202-208.

Montori, V.M., Helgemoe, P.K., Guyatt, G.H., Dean, D.S., Leung, T.W., Smith, S.A. and Kudva, Y.C., 2004. Telecare for patients with type 1 diabetes and inadequate glycemic control. *Diabetes care*, *27*(5), pp.1088-1094.

Montori 2004

Biermann E, Dietrich W, Rihl J, Standl E: Are there time and cost savings by using telemanagement for patients on intensified insulin therapy? A randomised, controlled trial. Comput Methods Programs Biomed 69:137-146, 2002

Ahring KK, Ahring JP, Joyce C, Farid NR: Telephone modem access improves diabetes control in those with insulin-requiring diabetes. Diabetes Care 15:971–975, 1992

Chase HP, Pearson JA, Wightman C, Roberts MD, Oderberg AD, Garg SK: Modem transmission of glucose values reduces the costs and need for clinic visits. Diabetes Care 26:1475–1479, 2003

Marrero DG, Vandagriff JL, Kronz K, Fineberg NS, Golden MP, Gray D, Orr DP, Wright JC, Johnson NB: Using telecommunication technology to manage children with diabetes: the Computer Linked Outpatient Clinic (CLOC) Study. Diabetes Educ 21:313–319, 1995

Wojcicki JM, Ladyzynski P, Krzymien J, Jozwicka E, Blachowicz J, Janczewska E, Czajkowski K, Karnafel W: What we canreally expect from telemedicine in intensive diabetes treatment: results from 3-year study on type 1 pregnant diabetic women. Diabetes Technol Ther 3:581–589,2001

Gomez EJ, Hernando ME, Garcia A, Del Pozo F, Cermeno J, Corcoy R, Brugues E, De Leiva A: Telemedicine as a tool for intensive management of diabetes: the DIABTel experience. Comput Methods Programs Biomed 69:163 –177, 2002

Welch G, Sokolove M, Mullin C, Master P, Horton E: Use of a modem-equipped blood glucose meter augmented with biweekly educator telephone support low-rs HbA1c in type 1 diabetes (Abstract). Diabetes 52 (Suppl. 1):A100, 2003

Medical Advisory Secritariat 2009

Ralston, J.D., Hirsch, I.B., Hoath, J., Mullen, M., Cheadle, A. and Goldberg, H.I., 2009. Web-based collaborative care for type 2 diabetes a pilot randomized trial. *Diabetes care*, *32*(2), pp.234-239.

Yoon, K.H. and Kim, H.S., 2008. A short message service by cellular phone in type 2 diabetic patients for 12 months. *Diabetes research and clinical practice*, *79*(2), pp.256-261.

Kim, S.I. and Kim, H.S., 2008. Effectiveness of mobile and internet intervention in patients with obese type 2 diabetes. *International journal of medical informatics*, *77*(6), pp.399-404.

Harno, K., Kauppinen-Mäkelin, R. and Syrjäläinen, J., 2006. Managing diabetes care using an integrated regional e-health approach. *Journal of telemedicine and telecare*, *12*(suppl 1), pp.13-15.

Shea, S., Weinstock, R.S., Teresi, J.A., Palmas, W., Starren, J., Cimino, J.J., Lai, A.M., Field, L., Morin, P.C., Goland, R. and Izquierdo, R.E., 2009. A randomized trial comparing telemedicine case management with usual care in older, ethnically diverse, medically underserved patients with diabetes mellitus: 5 year results of the IDEATel study. *Journal of the American Medical Informatics Association*, *16*(4), pp.446-456.

McMahon, G.T., Gomes, H.E., Hohne, S.H., Hu, T.M.J., Levine, B.A. and Conlin, P.R., 2005. Web-based care management in patients with poorly controlled diabetes. *Diabetes care*, *28*(7), pp.1624-1629.

Cho, J.H., Chang, S.A., Kwon, H.S., Choi, Y.H., Ko, S.H., Moon, S.D., Yoo, S.J., Song, K.H., Son, H.S., Kim, H.S. and Lee, W.C., 2006. Long-term effect of the Internet-based glucose monitoring system on HbA1c reduction and glucose stability. *Diabetes Care*, *29*(12), pp.2625-2631.

Bond, G.E., Burr, R., Wolf, F.M., Price, M., McCurry, S.M. and Teri, L., 2007. The effects of a web-based intervention on the physical outcomes associated with diabetes among adults age 60 and older: a randomized trial. *Diabetes technology & therapeutics*, *9*(1), pp.52-59.

Mushcab 2015

Stone, R.A., Rao, R.H., Sevick, M.A., Cheng, C., Hough, L.J., Macpherson, D.S., Franko, C.M., Anglin, R.A., Obrosky, D.S. and DeRubertis, F.R., 2010. Active care management supported by home telemonitoring in veterans with type 2 diabetes. *Diabetes care*, *33*(3), pp.478-484.

Cho, J.H., Chang, S.A., Kwon, H.S., Choi, Y.H., Ko, S.H., Moon, S.D., Yoo, S.J., Song, K.H., Son, H.S., Kim, H.S. and Lee, W.C., 2006. Long-term effect of the Internet-based glucose monitoring system on HbA1c reduction and glucose stability. *Diabetes Care*, *29*(12), pp.2625-2631.

Istepanian, R.S., Zitouni, K., Harry, D., Moutosammy, N., Sungoor, A., Tang, B. and Earle, K.A., 2009. Evaluation of a mobile phone telemonitoring system for glycaemic control in patients with diabetes. *Journal of Telemedicine and Telecare*, *15*(3), pp.125-128.

Rodríguez-Idígoras, M.I., Sepúlveda-Muñoz, J., Sánchez-Garrido-Escudero, R., Martínez-González, J.L., Escolar-Castelló, J.L., Paniagua-Gómez, I.M., Bernal-López, R., Fuentes-Simón, M.V. and Garófano-Serrano, D., 2009. Telemedicine influence on the follow-up of type 2 diabetes patients. *Diabetes technology & therapeutics*, *11*(7), pp.431-437.

Cho, J.H., Lee, H.C., Lim, D.J., Kwon, H.S. and Yoon, K.H., 2009. Mobile communication using a mobile phone with a glucometer for glucose control in Type 2 patients with diabetes: as effective as an Internet-based glucose monitoring system. *Journal of Telemedicine and Telecare*, *15*(2), pp.77-82.

Kim, C.S., Park, S.Y., Kang, J.G., Lee, S.J., Ihm, S.H., Choi, M.G. and Yoo, H.J., 2010. Insulin dose titration system in diabetes patients using a short messaging service automatically produced by a knowledge matrix. *Diabetes technology & therapeutics*, *12*(8), pp.663-669.

Bujnowska-Fedak, M.M., Puchała, E. and Steciwko, A., 2011. The impact of telehome care on health status and quality of life among patients with diabetes in a primary care setting in Poland. *Telemedicine and e-Health*, *17*(3), pp.153-163.

Chen, S.Y., Chang, Y.H., Hsu, H.C., Lee, Y.J., Hung, Y.J. and Hsieh, C.H., 2011. One-year efficacy and safety of the telehealth system in poorly controlled type 2 diabetic patients receiving insulin therapy. *Telemedicine and e-Health*, *17*(9), pp.683-687.

Tang, P.C., Overhage, J.M., Chan, A.S., Brown, N.L., Aghighi, B., Entwistle, M.P., Hui, S.L., Hyde, S.M., Klieman, L.H., Mitchell, C.J. and Perkins, A.J., 2013. Online disease management of diabetes: engaging and motivating patients online with enhanced resources-diabetes (EMPOWER-D), a randomized controlled trial. *Journal of the American Medical Informatics Association*, *20*(3), pp.526-534.

Polisena 2009

Biermann, E., Dietrich, W., Rihl, J. and Standl, E., 2002. Are there time and cost savings by using telemanagement for patients on intensified insulin therapy?: A randomised, controlled trial. *Computer methods and programs in biomedicine*, *69*(2), pp.137-146.

Chase, H.P., Pearson, J.A., Wightman, C., Roberts, M.D., Oderberg, A.D. and Garg, S.K., 2003. Modem transmission of glucose values reduces the costs and need for clinic visits. *Diabetes Care*, *26*(5), pp.1475-1479.

Harno, K., Kauppinen-Mäkelin, R. and Syrjäläinen, J., 2006. Managing diabetes care using an integrated regional e-health approach. *Journal of telemedicine and telecare*, *12*(suppl 1), pp.13-15.

Jansà, M., Vidal, M., Viaplana, J., Levy, I., Conget, I., Gomis, R. and Esmatjes, E., 2006. Telecare in a structured therapeutic education programme addressed to patients with type 1 diabetes and poor metabolic control. *Diabetes research and clinical practice*, *74*(1), pp.26-32.

Kim, H.S. and Oh, J.A., 2003. Adherence to diabetes control recommendations: impact of nurse telephone calls. *Journal of advanced nursing*, *44*(3), pp.256-261.

Kwon, H.S., Cho, J.H., Kim, H.S., Song, B.R., Ko, S.H., Lee, J.M., Kim, S.R., Chang, S.A., Kim, H.S., Cha, B.Y. and Lee, K.W., 2004. Establishment of blood glucose monitoring system using the internet. *Diabetes care*, *27*(2), pp.478-483.

Ładyżyński, P. and Wójcicki, J.M., 2007. Home telecare during intensive insulin treatment–metabolic control does not improve as much as expected. *Journal of telemedicine and telecare*, *13*(1), pp.44-47.

Maljanian, R., Grey, N., Staff, I. and Conroy, L., 2005. Intensive telephone follow-up to a hospital-based disease management model for patients with diabetes mellitus. *Disease Management*, *8*(1), pp.15-25.

McMahon, G.T., Gomes, H.E., Hohne, S.H., Hu, T.M.J., Levine, B.A. and Conlin, P.R., 2005. Web-based care management in patients with poorly controlled diabetes. *Diabetes care*, *28*(7), pp.1624-1629.

Piette, J.D., Weinberger, M. and McPhee, S.J., 2000. The effect of automated calls with telephone nurse follow-up on patient-centered outcomes of diabetes care: a randomized, controlled trial. *Medical care*, *38*(2), pp.218-230.

Piette, J.D., Weinberger, M., Kraemer, F.B. and McPhee, S.J., 2001. Impact of automated calls with nurse follow-up on diabetes treatment outcomes in a department of veterans affairs health care system. *Diabetes care*, *24*(2), pp.202-208.

Shea, S., Weinstock, R.S., Starren, J., Teresi, J., Palmas, W., Field, L., Morin, P., Goland, R., Izquierdo, R.E., Wolff, L.T. and Ashraf, M., 2006. A randomized trial comparing telemedicine case management with usual care in older, ethnically diverse, medically underserved patients with diabetes mellitus. *Journal of the American Medical Informatics Association*, *13*(1), pp.40-51.

Thompson, D.M., Kozak, S.E. and Sheps, S., 1999. Insulin adjustment by a diabetes nurse educator improves glucose control in insulin-requiring diabetic patients: a randomized trial. *Canadian Medical Association Journal*, *161*(8), pp.959-962.

Warren L, Whitlock R, Brown A et al. Telemedicine improved diabetic management. Mil Med 2000; 165: 579–584

Wojcicki, J.M., Ladyzynski, P., Krzymien, J., Jozwicka, E., Blachowicz, J., Janczewska, E., Czajkowski, K. and Karnafel, W., 2001. What we can really expect from telemedicine in intensive diabetes treatment: results from 3-year study on type 1 pregnant diabetic women. *Diabetes technology & therapeutics*, *3*(4), pp.581-589.

Wong, F.K.Y., Mok, M.P.H., Chan, T. and Tsang, M.W., 2005. Nurse follow-up of patients with diabetes: randomized controlled trial. *Journal of advanced nursing*, *50*(4), pp.391-402.

Saffari 2014

H.S. Kim, A randomized controlled trial of a nurse short-message service by cellular phone for people with diabetes, Int. J. Nurs.Stud. 44 (2007) 687–692.

Z. Faridi, L. Liberti, K. Shuval, V. Northrup, A. Ali, D.L. Katz, Evaluating the impact of mobile telephone technology on type 2 diabetic patients' self-management: the NICHE pilot study, J. Evaluation Clin. Pract. 14 (2008) 465–469.

K.H. Yoon, H.S. Kim, A short message service by cellular phone in type 2 diabetic patients for 12 months, Diab. Res. Clin. Pract. 79 (2008) 256–261.

H.J. Yoo, M.S. Park, T.N. Kim, S.J. Yang, G.J. Cho, T.G. Hwang, S.H. Baik, D.S. Choi, G.H. Park, K.M. Choi, A ubiquitous chronic disease care system using cellular phones and the Internet, Diab. Med. Br. Diab. Assoc. 26 (2009) 628–635.

C.S. Kim, S.Y. Park, J.G. Kang, S.J. Lee, S.H. Ihm, M.G. Choi, H.J.Yoo, Insulin dose titration system in diabetes patients using a short messaging service automatically produced by a knowledge matrix, Diab. Technol. Therapeut. 12 (2010) 663–669.

A.S. Shetty, S. Chamukuttan, A. Nanditha, R.K. Raj, A. Ramachandran, Reinforcement of adherence to prescription recommendations in Asian Indian diabetes patients using short message service (SMS) – a pilot study, J. Assoc. Phys. India 59 (2011) 711–714.

W.I. Hussein, K. Hasan, A.A. Jaradat, Effectiveness of mobile phone short message service on diabetes mellitus management; the SMS-DM study, Diab. Res. Clin. Pract. 94 (2011) e24–e26.

M. Goodarzi, I. Ebrahimzadeh, A. Rabi, B. Saedipoor, M.A. Jafarabadi, Impact of distance education via mobile phone text messaging on knowledge, attitude, practice and self efficacy of patients with type 2 diabetes mellitus in Iran, J. Diab. Metabolic Disorders 11 (2012) 10.

S. Arora, A.L. Peters, E. Burner, C.N. Lam, M. Menchine, Trial to Examine Text Message-based mHealth in emergency department patients with diabetes (TExT-MED): a randomized controlled trial, Ann. Emerg. Med. (2013)

Small 2013

Dale J, Caramlau I, Sturt J, Friede T, Walker R: Telephone peer-delivered intervention for diabetes motivation and support: the telecare explora tory RCT. Pat Ed Couns 2009, 75: 91 – 98

Turner B, Hollenbeak C, Liang Y, Pandit K, Joseph S, Weiner M: A randomised trial of peer coach and office staff support to reduce coronary heart disease risk in African-Americans with uncontrolled hypertension. JGenInternMed 2012, 27: 1258 – 1264.

Walker E, Blanco E, Shmukler C, Scollan-Koliopoulus M, Ullman R, Cohen H: Results of a successful telephonic intervention to improve diabetes control in urban adults. Diab Care 2011, 34: 2 – 7.

Heisler M, Vijan S, Makki F, Piette J: Diabetes control with reciprocal peer support versus nurse care management: a randomized trial. Annals Int Med 2010, 153: 507 – 515.

Samuel-Hodge C, Keyserling TC, Park S, Johnston L, Bangdiwala S: A randomized trial of a church-based diabetes self-management program for African Americans with type 2 diabetes. Diab Edu 2009, 35: 439 – 454.

Parry MW-WJ, Hodnett E, Tranmer J, Dennis C, Brooks D: Cardiac Home Education and Support Trial (CHEST): a pilot study. Canadian J Cardiol 2009, 25: e393 – e398.

Batik O, Phelan E, Walwick J, Wang G, LoGerfo J: Translating a community- based motivational support program to increase physical activity among older adults with diabetes at community clinics: a pilot study of Physical Activity for a Lifetime of Success (PALS). Prev Chron Disease 2008, 5: 1 – 7.

Carroll D, Rankin S, Cooper B: The effects of a collaborative peer advisor/ advanced practice nurse intervention: cardiac rehabilitation participation and rehospitalization in older adults after a cardiac event. J Cardio Nurs 2007, 22: 313 – 319.

Young RTJ, Friede T, Hollis S, Mason J, Lee P, Burns E, et al : Pro-active call center treatment support (PACCTS) to improve glucose control in type 2 diabetes: a randomized controlled trial. Diab Care 2005, 28: 278 – 282.

Keyserling T, Samuel-Hodge C, Ammerman A, Ainsworth B, Henriquez- Roldan C, Elasy T, et al: A randomized trial of an intervention to improve self-care behaviors of African-American women with type 2 diabetes: impact on physical activity. Diab Care 2002, 25: 1576 – 1583

Suksomboon 2014

Bogner HR, Morales KH, de Vries HF, Cappola AR (2012) Integrated management of type 2 diabetes mellitus and depression treatment to improve medication adherence: a randomized controlled trial. Ann Fam Med 10:15–22.

Dale J, Caramlau I, Sturt J, Friede T, Walker R (2009) Telephone peer-delivered intervention for diabetes motivation and support: the telecare exploratory RCT. Patient Educ Couns 75:91–98.

Howells L, Wilson AC, Skinner TC, Newton R, Morris AD, et al. (2002) A randomized control trial of the effect of negotiated telephone support on glycemic control in young people with Type 1 diabetes. Diabetic Med 19:643–648.

Walker EA, Shmukler C, Ullman R, Blanco E, Scollan-Koliopoulus M, et al. (2011) Results of a successful telephonic intervention to improve diabetes control in urban adults: a randomized trial. Diabetes Care 34:2–7.

Whittemore R, Melkus GD, Sullivan A, Grey M (2004) A nurse-coaching intervention for women with type 2 diabetes. Diabetes Educ 30:795–804

Sutcliffe 2011

Rami B, Popow C, Horn W, Waldhoer T, Schober E: Telemedical support to improve glycemic control in adolescents with type 1 diabetes mellitus. Euro J of Pediatrics 2006, 165 :701-705

Chase HP, Pearson JA, Wightman C, Roberts MD, Oderberg AD, Garg SK: Modem transmission of glucose values reduces the costs and need for clinic visits. Diabetes Care 2003, 26 :1475-1479.

Farmer AJ, Gibson OJ, Dudley C, Bryden K, Hayton PM, Tarassenko L, Neil A: A randomized controlled trial of the effect of real-time telemedicine support on glycemic control in young adults with type 1 diabetes. Diabetes Care 2005, 28 :2697-2702.

Franklin VL, Waller A1, Pagliari C, Greene SA: A randomized controlled trial of Sweet Talk, a text-messaging system to support young people with diabetes. Diabetic Medicine 2006, 23 :1332-1338.

Gay CL, Chapuis F, Bendelac N, Tixier F, Treppoz S, Nicolino M: Reinforced follow-up for children and adolescents with type 1 diabetes and inadequate glycaemic control: a randomized controlled trial intervention via the local pharmacist and telecare. Diabetes & Metabolism 2006, 32:159-165.

Howells L, Wilson AC, Skinner TC, Newton R, Morris AD, Greene SA: A randomized control trial of the effect of negotiated telephone support on glycaemic control in young people with Type 1 diabetes. Diabetic Medicine 2002, 19

:643-648.

Marrero DG, Vandagriff JL, Kronz K, Fineberg NS, Golden MP, Gray D, Orr DP, Wright JC, Johnson NB: Using telecommunication technology to manage children with diabetes: the Computer-Linked Outpatient Clinic (CLOC) Study. The Diabetes Educator 1995, 21 :313-319.

Nunn E, King B, Smart C, Anderson D: A randomized controlled trial of telephone calls to young patients with poorly controlled type 1 diabetes. Pediatric Diabetes 2006, 7 :254-259.

Rosenfalck AM, Bendtson I: The Diva System, a computerized diary, used in young type 1 diabetic patients. Diabetes Metab 1993, 19, :25-29

Viana 2016

Montori VM, Helgemoe PK, Guyatt GH, Dean DS, Leung TW, Smith SA, et al. Telecare for patients with type 1 diabetes and inadequate glycemic control: a randomized controlled trial and meta-analysis. Diabetes Care. 2004;27:1088–94.

Lawson ML, Cohen N, Richardson C, Orrbine E, Pham B. A randomized trial of regular standardized telephone contact by a diabetes nurse educator in adolescents with poor diabetes control. Pediatr Diabetes. 2005;6:32–40.

Farmer AJ, Gibson OJ, Dudley C, Bryden K, Hayton PM, Tarassenko L, et al. A randomized controlled trial of the effect of real-time telemedicine support on glycemic control in young adults with type 1 diabetes (ISRCTN46889446). Diabetes Care. 2005;28:2697–702.

Landau Z, Mazor-Aronovitch K, Boaz M, Blaychfeld-Magnazi M, Graph-Barel C, Levek-Motola N, et al. The effectiveness of Internet-based blood glucose monitoring system on improving diabetes control in adolescents with type 1 diabetes. Pediatr Diabetes. 2012;13:203–7.

Gay CL, Chapuis F, Bendelac N, Tixier F, Treppoz S, Nicolino M. Reinforced follow-up for children and adolescents with type 1 diabetes and inadequate glycaemic control: a randomized controlled trial intervention via the local pharmacist and telecare. Diabetes and Metabolism. 2006;32:159–65.

Esmatjes E, Jansa M, Roca D, Perez-Ferre N, del Valle L, Martinez-Hervas S,et al. The efficiency of telemedicine to optimize metabolic control inpatients with type 1 diabetes mellitus: Telemed study. Diabetes Technol Ther. 2014;16:435–41

Verhoeven 2007

Bellazzi R, Arcelloni M, Bensa G, Blankenfeld H, Brugués E, Carson E, Cobelli C, Cramp D, D'Annunzio G, De Cata P, De Leiva A, Deutsch T, Fratino P, Gazzaruso C, Garcìa A, Gergely T, Gómez E, Harvey F, Ferrari P, Hernando E, Boulos MK, Larizza C, Ludekke H, Maran A, Nucci G, Pennati C, Ramat S,

Roudsari A, Rigla M, Stefanelli M. Design, methods, and evaluation directions of a multi-access service for the management of diabetes mellitus patients. Diabetes Technol Ther. 2003;5(4):621-9.

Biermann E, Dietrich W, Rihl J, Standl E. Are there time and cost savings by using telemanagement for patients on intensified insulin therapy? A randomised, controlled trial. Comput Methods Programs Biomed. 2002 Aug;69(2):137-46.

Farmer AJ, Gibson OJ, Dudley C, Bryden K, Hayton PM, Tarassenko L, Neil A. A randomized controlled trial of the effect of real-time telemedicine support on glycemic control in young adults with type 1 diabetes (ISRCTN 46889446). Diabetes Care. 2005 Nov;28(11):2697-702.

Gay CL, Chapuis F, Bendelac N, Tixier F, Treppoz S, Nicolino M. Reinforced follow-up for children and adolescents with type 1 diabetes and inadequate glycaemic control: a randomized controlled trial intervention via the local pharmacist and telecare. Diabetes Metab. 2006 Apr;32(2):159-65.

Jansà M, Vidal M, Viaplana J, Levy I, Conget I, Gomis R, Esmatjes E. Telecare in a structured therapeutic education programme addressed to patients with type 1 diabetes and poor metabolic control. Diabetes Res Clin Pract. 2006 Oct;74(1):26-32.

Larizza C, Bellazzi R, Stefanelli M, Ferrari P, De Cata P, Gazzaruso C, Fratino P, D'Annunzio G, Hernando E, Gomez EJ. The M2DM Project--the experience of two Italian clinical sites with clinical evaluation of a multi-access service for the management of diabetes mellitus patients. Methods Inf Med. 2006;45(1):79-84.

McKay, H.G., Glasgow, R.E., Feil, E.G., Boles, S.M. and Barrera Jr, M., 2002. Internet-based diabetes self-management and support: Initial outcomes from the Diabetes Network project. *Rehabilitation Psychology*, *47*(1), p.31.

McMahon GT, Gomes HE, Hickson Hohne S, Hu TM, Levine BA, Conlin PR. Web-based care management in patients with poorly controlled diabetes. Diabetes Care. 2005 Jul;28(7):1624-9.

Dansky KH, Palmer L, Shea D, Bowles KH. Cost analysis of telehomecare. Telemed J E Health. 2001 Fall;7(3):225-32.

Izquierdo RE, Knudson PE, Meyer S, Kearns J, Ploutz-Snyder R, Weinstock RS. A comparison of diabetes education administered through telemedicine versus in person. Diabetes Care. 2003 Apr;26(4):1002-7.

Whitlock WL, Brown A, Moore K, Pavliscsak H, Dingbaum A, Lacefield D, Buker K, Xenakis S. Telemedicine improved diabetic management. Mil Med. 2000 Aug;165(8):579-84.

Verhoeven 2010

Biermann E, Dietrich W, Rihl J, Standl E. Are there time and cost savings by using telemanagement for patients on intensifed insulin therapy? A randomised, controlled trial. Comput Methods Programs Biomed. 2002;69 (2): 137–46.

Bellazzi R, Arcelloni M, Bensa G, Blankenfeld H, Brugués E, Carson E, Cobelli C, Cramp D, D'Annunzio G, De Cata P, De Leiva A, Deutsch T, Fratino P, Gazzaruso C, Garcìa A, Gergely T, Gómez E, Harvey F, Ferrari P, Hernando E, Boulos MK, Larizza C, Ludekke H, Maran A, Nucci G, Pennati C, Ramat S, Roudsari A, Rigla M, Stefanelli M. Design, methods, and evaluation directions of a multi-access service for the management of diabetes mellitus patients. Diabetes Technol Ther. 2003;5 (4): 621–9.

Boaz M, Hellman K, Wainstein J. An automated telemedicine system improves patient-reported well-being. Diabetes Technol Ther. 2009;11 (3): 181–6.

Bujnowska-Fedak MM, Puchała E, Steciwko A. Telemedicine for diabetes support in family doctors' practices: A pilot project. J Telemed Telecare. 2006;12 (Suppl 1): 8–10.

Cho JH, Lee HC, Lim DJ, Kwon HS, Yoon KH. Mobile communication using a mobile phone with a glucometer for glucose control in Type 2 patients with diabetes: As effective as an Internet-based glucose monitoring system. J Telemed Telecare. 2009;15 (2): 77–82.

Dale J, Caramlau I, Sturt J, Friede T, Walker R. Telephone peer-delivered intervention for diabetes motivation and support: The telecare exploratory RCT. Patient Educ Couns. 2009;75 (1): 91–8.

Farmer AJ, Gibson OJ, Dudley C, Bryden K, Hayton PM, Tarassenko L, Neil A. A randomized controlled trial of the effect of real-time telemedicine support on glycemic control in young adults with type 1 diabetes (ISRCTN 46889446). Diabetes Care. 2005;28 (11): 2697–702.

García-Sáez G, Hernando ME, Martinez-Sarriegui I, Rigla M, Torralba V, Brugues E, de Leiva A, Gomez EJ. Architecture of a wireless Personal Assistant for telemedical diabetes care. Int J Med Inform. 2009;78 (6): 391–403.

Gay CL, Chapuis F, Bendelac N, Tixier F, Treppoz S, Nicolino M. Reinforced follow-up for children and adolescents with type 1 diabetes and inadequate glycaemic control: A randomized controlled trial intervention via the local pharmacist and telecare. Diabetes Metab. 2006;32 (2): 159–65.

Hanauer DA, Wentzell K, Laffel N, Laffel LM. Computerized Automated Reminder Diabetes System (CARDS): E-mail and SMS cell phone text messaging reminders to support diabetes management. Diabetes Technol Ther. 2009;11 (2): 99–106.

Harno K, Kauppinen-Mäkelin R, Syrjäläinen J. Managing diabetes care using an integrated regional e-health approach. J Telemed Telecare. 2006;12 Suppl 1: 13–5.

Homko CJ, Santamore WP, Whiteman V, Bower M, Berger P, Geifman-Holtzman O, Bove AA. Use of an Internet-based telemedicine system to manage underserved women with gestational diabetes mellitus. Diabetes Technol Ther. 2007;9 (3): 297–306.

Jansà M, Vidal M, Viaplana J, Levy I, Conget I, Gomis R, Esmatjes E. Telecare in a structured therapeutic education programme addressed to patients with type 1 diabetes and poor metabolic control. Diabetes Res Clin Pract. 2006;74 (1): 26–32.

Ladyzynski P, Wojcicki JM. Home telecare during intensive insulin treatment–metabolic control does not improve as much as expected. J Telemed Telecare. 2007;13 (1): 44–7.

Laffel LM, Hsu WC, McGill JB, Meneghini L, Volkening LK. Continued use of an integrated meter with electronic logbook maintains improvements in glycemic control beyond a randomized, controlled trial. Diabetes Technol Ther. 2007;9 (3): 254–64.

Larizza C, Bellazzi R, Stefanelli M, Ferrari P, De Cata P, Gazzaruso C, Fratino P, D'Annunzio G, Hernando E, Gomez EJ. The M2DM Project–the experience of two Italian clinical sites with clinical evaluation of a multi-access service for the management of diabetes mellitus patients. Methods Inf Med. 2006;45 (1): 79–84.

Lee TI, Yeh YT, Liu CT, Chen PL. Development and evaluation of a patient-oriented education system for diabetes management. Int J Med Inform. 2007;76 (9): 655–63.

McKay HG, Glasgow R, Feil EG, Boles SM, Barrera M. Internet-based diabetes self-management and support: Initial outcomes from the Diabetes Network Project. Rehabil Psychol. 2002;47 (1): 31–48.

McMahon GT, Gomes HE, Hohne S Hickson, Hu TM, Levine BA, Conlin PR. Web-based care management in patients with poorly controlled diabetes. Diabetes Care. 2005;28 (7): 1624–9.

Nunn E, King B, Smart C, Anderson D. A randomized controlled trial of telephone calls to young patients with poorly controlled type 1 diabetes. Pediatr Diabetes. 2006;7 (5): 254–9.

Quinn CC, Clough SS, Minor JM, Lender D, Okafor MC, Gruber-Baldini A. WellDoc mobile diabetes management randomized controlled trial: Change in clinical and behavioral outcomes and patient and physician satisfaction. Diabetes Technol Ther. 2008;10 (3): 160–8.

Ralston JD, Hirsch IB, Hoath J, Mullen M, Cheadle A, Goldberg HI. Web-based collaborative care for type 2 diabetes: A pilot randomized trial. Diabetes Care. 2009;32 (2): 234–9.

Rami B, Popow C, Horn W, Waldhoer T, Schober E. Telemedical support to improve glycemic control in adolescents with type 1 diabetes mellitus. Eur J Pediatr. 2006;165 (10): 701–5.

Smith SA, Shah ND, Bryant SC, Christianson TJ, Bjornsen SS, Giesler PD, Krause K, Erwin PJ, Montori VM; Evidens Research Group. Chronic care model and shared care in diabetes: Randomized trial of an electronic decision support system. Mayo Clin Proc. 2008;83 (7): 747–57.

Timmerberg BD, Wurst J, Patterson J, Spaulding RJ, Belz NE. Feasibility of using videoconferencing to provide diabetes education: A pilot study. J Telemed Telecare. 2009;15 (2): 95–7.

Wens 2008

A. Mease, W.L. Whitlock, A. Brown, K. Moore, H. Pavliscsak, A. Dingbaum, et al., Telemedicine improved diabetic management, Mil. Med. 165 (8) (2000) 579–584

J.D. Piette, M. Weinberger, F.B. Kraemer, S.J. McPhee, Impact of automated calls with nurse follow-up on diabetes treatment outcomes in a department of veterans affairs Health Care System, Diab. Care 24 (2) (2001) 202–208

Wu 2010

Kirkman MS, Weinberger M, Landsman PB, Samsa GP, Shortliffe EA, Simel DL

et al. A telephone-delivered intervention for patients with NIDDM. Diabetes Care 1994; 17 : 840–846.

Glasgow RE, Toobert D. Brief, computer-assisted diabetes dietary self-management counseling: effects on behavior, physiologic outcomes, and quality of life. Med Care 2000; 38 : 1062–1073.

Piette JD, Weinberger M, McPhee SJ, Mah CA, Kraemer FB, Crapo LM. The effect of automated calls with telephone nurse follow-up on patient-centred outcomes of diabetes care: a randomized controlled trial. Med Care 2000; 38 : 218–230.

Piette JD, Weinberger M, Kraemer FB, McPhee SJ. Impact of automated calls with nurse follow-up on diabetes treatment outcomes in a department of veterans affairs health care system. Diabetes Care 2001; 24 : 202–208.

Krein SL, Klamerus ML, Vijan S, Lee JL, Fitzgerald JT, Pawlow A et al. Case management for patients with poorly controlled diabetes: a randomized trial. Am J Med 2004; 116 : 732–739.

Young RJ, Taylor J, Friede T, Hollis S, Mason JM, Lee P et al. Pro-active call centre treatment support (PACCTS) to improve glucose control in Type 2 diabetes. Diabetes Care 2005; 28 : 278–282.

Sacco WP, Malone JI, Morrison AD, Friedman A, Wells K. Effect of a brief, regular telephone intervention by paraprofessionals for type 2 diabetes. J Behav Med 2009; 32 : 349–359.

Zhai 2014

Pressman AR, Kinoshita L, Kirk S, et al. A novel telemonitoring device for improving diabetes control: protocol and results from a randomized clinical trial. *Telemed J E Health* 2014; 20:109–114.

Tang PC, Overhage JM, Chan AS, et al. Online disease management of diabetes: engaging and motivating patients online with enhanced resources-diabetes (EMPOWER-D), a randomized controlled trial. *J Am Med Inform Assoc* 2013; 20:526–534.

Bogner HR, Morales KH, de Vries HF, Cappola AR. Integrated management of type 2 diabetes mellitus and depression treatment to improve medication adherence: a randomized controlled trial. *Ann Fam Med* 2012; 10:15–22.

Del Prato S, Nicolucci A, Lovagnini-Scher AC, et al. Telecare Provides comparable efficacy to conventional self-monitored blood glucose in patients with type 2 diabetes titrating one injection of insulin glulisine – the ELEONOR study. *Diabetes Technol Ther* 2012; 14:175–182.

Glasgow RE, Kurz D, King D, et al. Twelve-month outcomes of an Internet-based diabetes self-management support program. *Patient Educ Couns* 2012; 87:81–92.

Glasgow RE, Kurz D, King D, et al. Outcomes of minimal and moderate support versions of an internet-based diabetes self-management support program. *J Gen Intern Med* 2010; 25:1315–1322.

Goodarzi M, Ebrahimzadeh I, Rabi A, et al. Impact of distance education via mobile phone text messaging on knowledge, attitude, practice and self efficacy of patients with type 2 diabetes mellitus in Iran. *J Diabetes Metab Disord* 2012; 11:10.

Jarab AS, Alqudah SG, Mukattash TL, et al. Randomized controlled trial of clinical pharmacy management of patients with type 2 diabetes in an outpatient diabetes clinic in Jordan. *J Manag Care Pharm* 2012; 18:516–526.

Schechter CB, Cohen HW, Shmukler C, Walker EA. Intervention costs and cost-effectiveness of a successful telephonic intervention to promote diabetes control. *Diabetes Care* 2012; 35:2156–2160.

Bujnowska-Fedak MM, Puchała E, Steciwko A. The impact of telehome care on health status and quality of life among patients with diabetes in a primary care setting in Poland. *Telemed J E Health* 2011; 17:153–163.

Lim S, Kang SM, Shin H, et al. Improved glycemic control without hypoglycemia in elderly diabetic patients using the ubiquitous healthcare service, a new medical information system. *Diabetes Care* 2011; 34:308–313.

Luley C, Blaik A, Reschke K, et al. Weight loss in obese patients with type 2 diabetes: effects of telemonitoring plus a diet combination – the Active Body Control (ABC) Program. *Diabetes Res Clin Pract* 2011; 91:286–292.

Quinn CC, Shardell MD, Terrin ML, et al. Cluster-randomized trial of a mobile phone personalized behavioral intervention for blood glucose control. *Diabetes Care* 2011; 34:1934–1942.

Anderson DR, Christison-Lagay J, Villagra V, et al. Managing the space between visits: a randomized trial of disease management for diabetes in a community health center. *J Gen Intern Med* 2010; 25:1116–1122.

Lorig K, Ritter PL, Laurent DD, et al. Online diabetes self-management program: a randomized study. *Diabetes Care* 2010; 33:1275–1281.

Noh JH, Cho YJ, Nam HW, et al. Web-based comprehensive information system for self-management of diabetes mellitus. *Diabetes Technol Ther* 2010; 12:333–337.

Tildesley HD, Mazanderani AB, Ross SA. Effect of Internet therapeutic intervention on A1c levels in patients with type 2 diabetes treated with insulin. *Diabetes Care* 2010; 33:1738–1740.

Dale J, Caramlau I, Sturt J, et al. Telephone peer-delivered intervention for diabetes motivation and support: the telecare exploratory RCT. *Patient Educ Couns* 2009; 75:91–98.

Holbrook A, Thabane L, Keshavjee K, et al. Individualized electronic decision support and reminders to improve diabetes care in the community: COMPETE II randomized trial. *CMAJ* 2009; 181:37–44.

Istepanian RS, Zitouni K, Harry D, et al. Evaluation of a mobile phone telemonitoring system for glycaemic control in patients with diabetes. *J Telemed Telecare* 2009; 15:125–128.

Rodríguez-Idígoras MI, Sepúlveda-Muñoz J, Sánchez-Garrido-Escudero R, et al. Telemedicine influence on the follow-up of type 2 diabetes patients. *Diabetes Technol Ther* 2009; 11:431–437.

Yoo HJ, Park MS, Kim TN, et al. A Ubiquitous Chronic Disease Care system using cellular phones and the internet. *Diabet Med* 2009; 26:628–635.

Faridi Z, Liberti L, Shuval K, et al. Evaluating the impact of mobile telephone technology on type 2 diabetic patients' self-management: the NICHE pilot study. *J Eval Clin Prac* 2008; 14:465–469.

Kim SI, Kim HS. Effectiveness of mobile and internet intervention in patients with obese type 2 diabetes. *Int J Med Inform* 2008; 77:399–404.

Kim HS, Song MS. Technological intervention for obese patients with type 2 diabetes. *Appl Nurs Res* 2008; 21:84–89.

Yoon KH, Kim HS. A short message service by cellular phone in type 2 diabetic patients for 12 months. *Diabetes Res Clin Pract* 2008; 79:256–261.

Kim HS, Jeong HS. A nurse short message service by cellular phone in type-2 diabetic patients for six months. *J Clin Nurs* 2007; 16:1082–1087.

Kim HS. A randomized controlled trial of a nurse short-message service by cellular phone for people with diabetes. *Int J Nurs Stud* 2007; 44:687–692.

Bond GE, Burr R, Wolf FM, et al. The effects of a web-based intervention on the physical outcomes associated with diabetes among adults age 60 and older: a randomized trial. *Diabetes Technol Ther* 2007; 9:52–59.

Bond GE, Burr RL, Wolf FM, Feldt K. The effects of a web-based intervention on psychosocial well-being among adults aged 60 and older with diabetes: a randomized trial. *Diabetes Educ* 2010; 36:446–456.

Cho JH, Chang SA, Kwon HS, et al. Long-term effect of the Internet-based glucose monitoring system on HbA1c reduction and glucose stability: a 30-month follow-up study for diabetes management with a ubiquitous medical care system. *Diabetes Care* 2006; 29:2625–2631.

Kim CJ, Kang DH. Utility of a Web-based intervention for individuals with type 2 diabetes: the impact on physical activity levels and glycemic control. *Comput Inform Nurs* 2006; 24:337–345.

Maljanian R, Grey N, Staff I, Conroy L. Intensive telephone follow-up to a hospital-based disease management model for patients with diabetes mellitus. *Dis Manag* 2005; 8:15–25.

McMahon GT, Gomes HE, Hohne SH, et al. Web-based care management in patients with poorly controlled diabetes. *Diabetes Care* 2005; 28:1624–1629.

Wong FKY, Mok MPH, Chan T, Tsang MW. Nurse follow-up of patients with diabetes: randomized controlled trial. *J Adv Nurs* 2005; 50:391–402.

Kwon HS, Cho JH, Kim HS, et al. Establishment of blood glucose monitoring system using the internet. *Diabetes Care* 2004; 27:478–483.

Kim HS, Oh JA. Adherence to diabetes control recommendations: impact of nurse telephone calls. *J Adv Nurs* 2003; 44:256–261.

Piette JD, Weinberger M, Kraemer FB, McPhee SJ. Impact of automated calls with nurse follow-up on diabetes treatment outcomes in a Department of Veterans Affairs Health Care System: a randomized controlled trial. *Diabetes Care* 2001; 24:202–208.

Whitlock WL, Brown A, Moore K, et al. Telemedicine improved diabetic management. *Mil Med* 2000; 165:579–584.

Shea S, Weinstock RS, Teresi JA, et al. A randomized trial comparing telemedicine case management with usual care in older, ethnically diverse, medically underserved patients with diabetes mellitus: 5 year results of the IDEATel study. *J Am Med Inform Assoc* 2009; 16:446–456.
