# Supplementary material for: Telehealth Interventions to Support Self-Management of Long-Term Conditions: A Systematic Metareview of Diabetes, Heart Failure, Asthma, Chronic Obstructive Pulmonary Disease, and Cancer
Source: J Med Internet Res. 2017 May 17;19(5):e172. doi: 10.2196/jmir.6688 (PMC5451641; doi:10.2196/jmir.6688)
Supplement: Multimedia Appendix 3 [file jmir_v19i5e172_app3.pdf]

Characteristics of Included Reviews					
Review	Intervention of interest	Participant demographics	Setting, delivery mode,	Content, focus, and mode of instruction	Duration, intensity, and follow-up
Diabetes Reviews					
Baron 2012	Mobile telemonitoring of blood glucose	Type 1 diabetes – 2 RCTs – mean age 23.8 and 35.7, % male 43% and 59.1%, respectively<br>Type 2 diabetes – 10 RCTs – mean age ranged 46.9 to 63.9, % male ranged 36.6% to 78.3%	All interventions involved transmission of data from a mobile device to a central server, from where the participants would then obtain specific feedback.	Focus was on aiding patients in interpretation of self-monitored data with the aim of improving self-management.	Duration 3-12 months<br>Daily to fortnightly data transmission.<br>Follow-up ranged from 3-12 months
Beatty 2013	Internet based self-help programmes	Type 2 diabetes<br>Mean age ranged 40.6 to 61.5	3/5 "diabetes-Net" online programme with professional online support<br>1/5 "Internet Diabetes Self-Management Programme" – includes moderated forum<br>1/5 CD-ROM tailored self-management with professional telephone support	4/5 conducted by the same group, investigating different levels of professional input to an online diabetes self-management programme.	3/5 reported duration, range 6 to 8 weeks
Berat-arrechea 2014	Mobile health interventions	One type 2 diabetes. Two not specified.	Limited to low- and middle-income countries.<br>All interventions delivered by SMS.	One interventions involved SMS transmission of self-monitored glucose data with feedback from study doctor. Two educational interventions with SMS reminders	Glucose monitoring: daily.<br>One study followed up for 1 year.<br>Otherwise n.s.
Cassimatis 2012	Behavioural telehealth interventions	n.s.	Telephone counseling, videoconferencing, educational telephone based interventions. Interventions could not be primarily telemonitoring.	All educational in focus.<br>10/13 telephone support<br>1/13 video messaging<br>2/13 phone messaging supplementing nurse follow-up	5 weeks to 12 months duration<br>Follow up 3-12 months
Currell 2010	Telemedicine – use of telecommunications to provide care	Type 1 diabetes – 2 RCTs<br>Children over 5 years and patients aged 16-65, respectively	Transfer of self-monitored blood glucose readings via modem. Both also included clinic follow-up.	One RCT also included telephone counseling	Duration: 12 weeks to 12 months<br>Frequency: weekly to 2 weekly
De Jongh 2012	Mobile phone messaging for facilitating self-management	Type 1 diabetes – Youth and young adults	SMS sent between healthcare provider and participants' mobile phone.	1/2 – medication reminders<br>1/2 – reminders for blood glucose monitoring, automated feedback plus diabetes related educational messages	1 study, average 2.7 messages per day<br>1 study – 2 weekly
Farmer 2005	Telemedicine interventions supporting blood glucose self-monitoring	6/12 – type 1 diabetes<br>1/12 – both type 1 and 2 diabetes<br>2/12 did not specify<br>2/12 – pregnant participants with	Transmission of blood glucose results via a remote server.<br>Feedback was either automated of clinical delivered.<br>11/12 transmitted using telephone	11/12 evaluated telemedicine in addition to usual care.<br>4/12 transmitted self-management data (not	Duration 12 weeks to 1 year in the 9 pooled RCTs.<br>"Majority of trials" transmitted data weekly

		type 1 diabetes<br>1/12 – gestational diabetes	line or internet	specified) in addition to blood glucose	
Farmer 2016	Monitoring or brief messaging interventions to support medication adherence.	Type 2 diabetes	Most concerned mobile messaging. Some used specifically designed technologies such as electronic monitoring of blister packs.	Mobile messaging delivered by clinician. Algorithmic responded designed to support medication adherence.	Duration: 6 weeks to 1 year<br>Intensity: Daily to weekly
Flodgren 2015	Telemedicine interventions grouped into *remote monitoring, store and forward applications,* and *interactive (real-time) telemedicine*.	8 RCTs – type 1 diabetes<br>Mean age 9 to 41<br>One RCT of pregnant women with type 1 diabetes<br>6 RCTs – type 2 diabetes<br>Mean age 46 to 72<br>7 RCTs – mixed type 1 and 2 diabetes	Type 1 diabetes:<br>4 self-monitored glucometer data with transfer<br>1 smartphone application with data transfer<br>2 internet based interventions<br>1 videoconferencing intervention<br>Type 2 diabetes<br>1 mobile intervention<br>2 internet based interventions<br>1 glucometer with transfer<br>1 HTM unit<br>1 videoconferencing<br>Mixed diabetes<br>3 internet interventions<br>2 glucometer readings with transfer<br>2 videoconferencing interventions<br>1 HTM	11 RCTs had a specific educational focus.<br>All focused on review of clinical data – some self-monitored glucose data.	Data transfer daily to monthly<br>Follow-up 3 to 18 months
Garcia-Lizana 2007	Information communication technology (excluding telephone-only) for chronic disease management	1/7 children<br>1/7 aged 15/20<br>5/7 not specified	n.s.	4/7 involved transmission of blood sugar. 3 of these provided telephone feedback.<br>3/7 predominantly education delivery by internet (2/7) and 'telemedicine' (not defined) (1/7)	Follow-up 6 weeks to 12 months
Graziano 2009	Isolated telephone interventions for type 2 diabetes	5/8 type 2 diabetes only<br>1/8 95% type 2 diabetes<br>2/8 not specified<br>Mean age 60 years<br>7/8 reported insulin use – mean 34% in these studies	Outgoing calls to patients with type 2 diabetes	6/8 evaluated telephone-only interventions for glycaemic control<br>2/8 evaluated telephone components of broader interventions	Duration 3 to 12 months<br>5/8 twice weekly calls<br>2/8 reported number of contacts (3-4 and 13 respectively)<br>1/8 not reported<br>Follow-up 3-12 months
Greenwood 2014	Remote patient monitoring incorporating structured self-monitoring of blood glucose	Type 2 diabetes	Any method of remote interaction and healthcare delivery (including telephone, video, text message, vital signs monitoring)	Interventions involved at least one of seven elements of structured self-monitoring of blood glucose:<br>1. patient education<br>2. healthcare	n.s.

				provider education<br>3. specific self-monitoring profile (frequency and intensity)<br>4. Specific pre- and post-meal blood glucose goals<br>5. Blood glucose data used to modify behaviour<br>6. Evidence of feedback to patient<br>7. Evidence of 2-way interaction	
Hamine 2015	Mobile phone interventions to promote medication adherence	Results for diabetes used. No further details given	n.s. for diabetes RCTs separately from other trial types	n.s. for diabetes RCTs separately from other trial types	n.s. for diabetes RCTs separately from other trial types
Holtz 2012	Mobile phone interventions for diabetes management	57% of studies included type 1 diabetes only<br>19% type 2 diabetes<br>10% mixed<br>14% did not specify	71% used a study specific application, others simple messaging.<br>52% involved messaging	81% used a diary function<br>39% used educational messaging<br>71% used automatic transmission of blood glucose data from glucometer to phone	Duration 2 weeks to 1 year
Huang 2015	Self-monitored transmission of glucometer data with healthcare professional feedback.	Type 2 diabetes<br>% female ranged 38.75 to 71.67	5 telephone based<br>11 Internet based<br>2 automated	n.s.	Duration 3 to 60 months
Jaana 2007	Home telemonitoring (automated transmission of clinical data) for diabetes	Type 1 or 2 diabetes<br>Average age ranged from 13 to 69	11/13 – glucometer linked to phone, video or internet for transmission of data.<br>2/13 – electronic diary with data transmission/reminders.<br>Older studies tended to use phone only with internet and videoconferencing being used for feedback in more recent studies.	All involved transmission of blood glucose measurements.<br>3/13 also included lifestyle/behavioural data	Duration 3 to 15 months
Kok 2011	e-health interventions (interaction	9/12 RCTs concerned diabetes<br>5/9 type 2 diabetes<br>2/9 not specified	All web based, combination of videoconferencing, discussion, education website and email.	5/9 evaluated e-health in addition to usual care, 4/9 instead of usual care.	Follow-up: 3-30 months, and until birth in study of gestational diabetes

	between patient and healthcare provider via internet) to support self-management	1/9 type 1 or 2<br>1/9 indigent women with diabetes		9/9 offered web-based education<br>4/9 gave feedback on glucose monitoring data<br>2/9 incorporated videoconferencing	
Krishna 2008	Diabetes self-management via cell-phone	Type 1 or 2 diabetes<br>6/18 studies involving children<br>12/18 involving adults	All participants received face-to-face diabetes care with the intervention groups also receiving cell-phone based input in addition.<br>Delivery was by SMS, voice-mail, internet or email.	Interventions included educational messages, reminders, management plans and input of self-monitoring blood glucose data.	3-12 months duration<br>Daily to weekly input
Krishna 2009	Delivery of health information or education by mobile phone or messaging	9/25 studies focused on diabetes<br>4/9 type 1 diabetes<br>5/9 type 2 diabetes	Delivery by SMS (9/9) plus voice interaction (3/9) or internet (5/9).	All had an educational focus. 7/9 included support for blood glucose monitoring, medication dose and lifestyle. 1/9 focused on hypoglycaemic events.	Duration 3 to 12 months<br>Frequency – 2/9 daily, 7/9 weekly.
Kujipers 2012	Web-based interventions for patient empowerment and physical activity	Mean age 60 years (all conditions)	Web based – included online interaction with professional – usually study nurse	Online support promoting self-management and physical activity programmes with peer support and professional feedback.	Duration 6 weeks to 18 months.<br>Frequency and intensity "poorly reported in studies"
Liang 2011	Mobile phone interventions for diabetes self-management	9/22 type 1 diabetes<br>10/22 type 2 diabetes<br>2/22 mixed<br>1/22 unspecified<br>Mean age 44 years<br>Mean duration of diabetes 6.4 years	12/22 SMS and internet<br>8/22 SMS alone<br>2/22 compared SMS and internet	All contained reinforcement of lifestyle management<br>Most encouraged self-monitoring of blood glucose<br>5/22 tailored SMS interventions	14/22 daily<br>3/22 weekly<br>4/22 unspecified<br>Median follow-up 6 months (range 3-12 months)
Lieber 2011	Telemontoring of blood glucose data	4/5 type 2 diabetes<br>1/5 type 1 diabetes	n.s.	n.s.	Follow-up 6-12 months
Marcolino 2013	Telemedicine interventions for type 1 or type 2 diabetes	n.s.	8/13 primary care, 4/13 secondary care, 1/13 primary and secondary care<br>Exchange of clinical information between patient and healthcare provider by computer, videoconferencing, telephone or mobile device, in addition to usual care.	Interventions either monitored clinical values or combined monitoring with education.<br>Educational components included phone calls, videoconferencing, SMS, websites, educational reminders.	Duration: 6-18 months (6/13 6 months, 6/13 12 months, 1/13 18 months)<br>8/13 reported weekly data transmission
Montori 2004	Telecare (transmission of glucometer data with healthcare professional feedback) for	Type 1 diabetes.<br>7/8 adult patients<br>1/8 paediatric patients<br>All studies recruited patients with poor glycaemic control	Transmission of glucometer data with professional feedback	n.s.	Daily to 2 weekly transmission<br>Daily to 4 weekly feedback

	type 1 diabetes.				
Medical Advisory Secretariat 2009	Home telemonitoring	Type 2 diabetes<br>Mean age ranged 45 to 71 years	Self-monitored blood-glucose data transmitted to healthcare professional that provided feedback.<br>4/8 used automated, linked glucometers<br>4/8 manually sent data	Specific training given to intervention group, although content of this not reported.<br>Interventions also included videoconferencing (2/8), web-based education (5/8), monitoring of lifestyle factors (7/8).	Frequency poorly reported. Weekly to monthly when available.<br>Duration and follow-up 6-30 months.
Mushcab 2015	Web-based remote monitoring	Type 2 diabetes<br>Age data incomplete	Web-based transmission of self-monitored blood glucose data.	n.s.	Duration 3 to 18 months
Polisena 2009	“home telehealth” – transmission of clinical data from home to healthcare providor	Type 1 and 2 diabetes – 4 studies did not specify.<br>Patients with impaired cognitive function, language barrier or major chronic illnesses were excluded from most studies.	Home telemonitoring included synchronous (audio- and video-conferencing) and asynchronous (tramission via internet, video clips or electronic clinical data).<br>Telephone support involved phone contact only without the transmission of clinical or outcome data.	Home telemonitoring:<br>11/12 focused on tranmsision of blood glucose data<br>1/12 had an educational focus<br>2/12 included transmission of other biological data<br>1/12 reinforced lifestyle advice<br>Telephone support:<br>3/5 focused on lifestyle intervention<br>2/5 monitored blood glucose<br>4/5 had an education focus	Follow-up 3 months to 3 years<br>Home telemonitoring frequency – daily to 2 weekly<br>Telephone support frequency – weekly to 3 weekly.
Saffari 2014	Health education interventions delivered via mobile phone.	Type 2 diabetes<br>Mean age ranged 47 to 63<br>% males ranges 22.2 to 51	Mobile phone SMS delivery	Education focus including reminders and dietary/lifestyle information and prompts.	Duration 3 months to 1 year.
Small 2013	Telephone-based self-management interventions	Review focus on vascular diseases<br>7 RCTs of interventiions for type 2 diabetes. 1807 participants	Telephone interventions by trained ‘lay health workers’.	Educational content to telephone interventions. 3 RCTs specifically addressed adherence. 4 RCTs contained specific lifestyle interventions.	Intensity ranged from weekly to 3 monthly phone calls.
Suksomboon 2014	Telephone interventions for diabetes	n.s.	Telephone only interventions – excluded those with other technologies	2/5 educational focus<br>3/5 encouraged medication compliance<br>3/5 focused on problem solving and goal setting	Number of phone calls ranged from 2 to 16. Mean phone call length 9 to 15 minutes.
Sutcliffe 2011	ICT interventions to promote access and engagement of young people	776 young people (Males = 499, Females = 277) with type 1 diabetes. Study sizes ranged from 28 to 123 (Mean = 78.3, SD = 30.78). The mean age of	Video- or tele-conferencing<br>Telephone support<br>Novel electronic devices transferring clinical data<br>Web based discussion boards	n.s.	Duration 6-18 months

	with diabetes into healthcare	patients ranged from 11.9 to 23.9 years (Mean = 16, SD = 3.74)			
Verhoeven 2007	Teleconsultation and videoconferencing for diabetes	Type 1 or type 2 diabetes mellitus. Videoconferecing included only type 2 diabetes	Combination of primary and secondary care	8 RCTs – teleconsultation<br>3 RCTs - videoconferencing	n.s.
Verhoeven 2010	Comparison between synchronous and asynchronous teleconsultation	Type 1 or type 2 diabetes mellitus	Both primary and secondary care.	Unable to separate RCT data – n.s.	n.s. for RCT data
Viana 2016	Interventions aimed at improving patient compliance with therapies for glycaemic control in type 1 diabetes. Data for telecare used.	Type 1 diabetes.<br>Mean age ranged 13 to 43	2 RCTs – telephone based contact.<br>4 RCTs – self- monitored glucometer data transmitted via internet/mobile device.	Feedback aimed a promotion of adherence to management plans to improve HbA1c.<br>2 telephone based RCTs educational in focus.<br>4 involved direct monitoring of self-monitored blood glucose data.	Most 6 month duration, one 9 month.<br>Data tramsmission twice weekly to monthly.
Wens 2008	Educational interventions aimed at improving adherence to treatment recommend-ations in type 2 diabetes	Type 2 diabetes	2 RCTs delivered educational intervention via telemedicine. One telephone intervention. One telemedicine mediated 'visit' supplementing an educational class	Both educational in focus. Aimed at promoting adherence to lifestyle interventions as well as medication.	Weekly intervention
Wu 2010	Telephone follow-up	Type 2 diabetes, mean duration 8 years.<br>Mean age 63 years<br>50% female<br>13% controlled with diet only, 31% diet and oral agents, 56% on insulin.	All involved phone call to patient from a trained diabetes professional. Some also incorporated calls from patients to an automated service.	Lifestyle 2/7<br>Self-care 6/7<br>Glucose monitoring 5/7<br>Therapy adjustment 5/7<br>Psychological input 2/7<br>Guideline driven 2/7<br>Physician input 3/7	Follow-up 6 to 18 months<br>Call frequency biweekly to monthly
Zhai 2014	Telemedicine interventions (including web-based, teleconferencing, mobile and landline-based telephone interventions)	Type 2 diabetes.<br>Mean age ranged 46 to 70.<br>% male ranged 37 to 75	12 RCTs – telephone based interventions<br>19 RCTs – internet based interventions<br>6 RCTs – Internet mediated data transmission<br>2 RCTs – messaging based (SMS)	Telephone based interventions were all educational in focus. Internet mediated transfer involved clinical data monitoring. Internet-based interventions also involved uploading of self-monitored blood glucose data.	Duration 3-30 months
Heart Failure Reviews					
Berat-	Mobile health	Heart failure.	Limited to low- and middle-income	Disease counseling and	Weekly telephone

arrechea 2014	interventions		countries.<br>Delivery by mobile telephone	access to telephone helpline.	intervention
Chaudhry 2007	Telemonitoring systems (any technology, including telephone) for heart failure. Telephone support included educational component. Also considered separately automated physiological monitoring (not considered self-management.)	2627 participants (range 134-1518).<br>Mean age 65 to 72 years.<br>Percentage male ranged from 46 to 71.<br>Variation in severity of heart failure (NYHA IV ranged from 9-59%)	All involved monitoring from centralised nurses. Delivered by telephone call.	Symptom monitoring via live, nurse delivered, one-to-one phone call.<br>All were multi-faceted interventions which included an educational component addressing diet, medication adherence.<br>One intervention also included educational support to the patient's family.<br>One intervention included adjustment of diuretic doses, however this was under direction of the trial nurses.	Duration 6-12 months.<br>1 study weekly calls for 2 weeks then decreasing frequency. 2 studies 2 weekly calls for 8 weeks then decreasing frequency.<br>2 studies had 17 and 14 calls, respectively, over 6 months.
Ciere 2012	Telehealth interventions (home-based self-monitoring of signs and symptoms with data transferred to remote healthcare professionals) delivered to community-dwelling patients with heart failure in which measures of knowledge, perceived self-efficacy of self-care behaviours were reported	Mean age of participants ranged 61–78 years.<br>proportion of males ranged from 37–99%, although gender was unreported in two studies.<br>Seven studies reported baseline severity of HF based on NYHA. Most were mild or moderately impaired. Some studies included those with no impairment or severe impairment.	Community setting<br>7/11 – home telehealth system<br>4/11 – telephone<br>1/11 – website<br>2/11 - videophone	11/11 involved monitoring of signs and symptoms<br>9/11 included specific educational component	Duration 6 weeks to 12 months (median 3 months)<br>Most studies requested the patients use equipment daily.<br>Follow up ranged from 2-12 months
Clarke 2011	Telemonitoring for heart failure (excluded telephone-only interventions). Physiological monitoring but also addressed adherence.	3480 patients<br>Mean age 55-85<br>Reporting of comorbidities highly variable.<br>All had LVEF <40%, NYHA I-IV	All used specialised monitoring equipment, in the patient's home, connected via a phone line.	10/13 – daily physiological monitoring (weight, HR, BP, ECG)<br>2/13 – daily body weight plus symptom monitoring<br>All transmitted to central healthcare professional (often nurse)	Daily monitoring<br>Follow-up: 3-15 months

Garcia-Lizana 2007	Information communication technology (excluding telephone-only) for chronic disease management	n.s.	2/6 web based<br>2/6 videoconferencing<br>2/6 telemonitoring	1/6 specified feedback<br>1/6 specific education focus	Follow-up: 6-15 months
Inglis 2015	Telephone support - monitoring and/or self-care management delivered using telephone technology<br>Telemonitoring - digital/broadband/satellite/wireless or bluetooth transmission of physiologic data	13192 participants<br>Mean age ranged 44.5 to 78 years<br>Mean male participants 64%	Community based home intervention by telephone or telemonitoring	25.41 – structured telephone support including<br>18/41 – telemonitoring of vital signs, ECGs, weight.<br>2/41 – telephone support and telemonitoring	Follow-up 3-18 months<br>Intensity ranged from daily to weekly, with reducing frequency over time.
Kujipers 2012	Web-based interventions for patient empowerment and physical activity	Mean age 60 years (all conditions)	Web based – included online interaction with professional – usually study nurse	Online support promoting self-management and physical activity programmes with peer support and professional feedback.	Duration 3 to 12 months.<br>Frequency and intensity "poorly reported in included studies"
Radhakrishnan 2012	Telecommunication (excluding telephone only) with self-care outcomes	n.s.	In most studies data review and patient interaction carried out by trial nurse	Vital signs monitoring (5/8) videoconferencing (4/8) and tailored education content through questions and answers (4/8).	Duration 6 weeks-12 months.<br>Follow-up similar duration.<br>6 week intervention had additional 4 month follow-up
Schmidt 2010	Home telemonitoring<br>13/19 telephone monitoring<br>6/19 vital signs monitoring	n.s.	n.s.	n.s.	n.s.
Asthma Reviews					
Berat-arrechea 2014	Mobile health interventions		Limited to low- and middle-income countries.<br>All interventions delivered by SMS.	1 RCT – mobile based self-management plan with interactive feedback.<br>1 RCT – Mobile monitoring of spirometry data with feedback.	
De Jongh 2012	Mobile phone messaging for	Moderate persistent asthma, mean age 24.6 (1 RCT)	SMS	Participants submitted peak flow readings	Daily self-monitoring results sent to central server.

	facilitating self-management			Content of feedback not specified	Weekly review by healthcare professional with personalized feedback and advice.
Flodgren 2015	Telemedicine interventions grouped into *remote monitoring, store and forward applications,* and *interactive (real-time) telemedicine*.	5 RCTs of asthma interventions<br>2 RCTs children only – mean age 10 in both<br>2 RCTs adults only, mean ages 28 and 32<br>One adults and children (mean ages 45 and 10, respectively)	3 internet based interventions<br>1 computer based intervention involving camera and real time review<br>1 portable hand held data transmiting device with self-monitoring and input	All had educational focus and concerned review of clinical data with feedback	Follow-up 6-12 months<br>Data transfer weekly to fortnightly.
Garcia-Lizana 2007	Information communication technology (excluding telephone-only) for chronic disease management	All children or adolescents with asthma	3/5 – interactive computer game<br>2/5 web based educational intervention	All educational and interactive in focus	Follow-up: 3-15 months
Jaana 2009	Home telemonitoring (transfer of clinical data from home to healthcare centre) for respiratory conditions	Mean age 7.6 to 28 years	5/7 used specialised equiptment<br>3/7 utilised telephone connection<br>1/7 SMS<br>3/7 internet connection	7/7 evaluated asthma symptoms<br>6/7 assessed peak expiratory flow<br>5/7 symptom diary<br>3/7 evaluated medication use<br>3/7 assessed function status<br>1/7 spirometry values	Duration 3 to 12 months<br>Frequency twice daily in one RCT, daily in 5 RCTs, twice weekly in one RCT.
Krishna 2009	Delivery of health information or education by mobile phone or messaging	Asthma	SMS only	Peak flow monitoring and weekly medication adjustment	Duration 4 months<br>Frequency: weekly
Marcano Belisario 2013	Smartphone or tablet self-management apps for asthma	Asthma (although diagnostic criteria differed)<br>One RCT – aged 12 and over.<br>One RCT did not report age ranges.	Asthma self-management smartphone apps compared to traditional, paper-based, asthma self-management<br>Outpatient setting	Data on symptoms, medication usage, peak expiratory flow etc. was recorded on app and transmitted intermittently to central server. Data then transmitted back along with control analysis and self-management recommendations.	Twice daily app usage in one study, other not specified.<br>3-6 month follow-up
McLean 2010	Home telecare. Broad definition,	Asthma	9 RCTs – telephone interventions<br>2 RCTs – videoconferencing	Majority incorporated and initial face-to-face	Daily to 2 weekly contact.<br>3 to 18 month follow-up

	included video, telephone or internet links with health professionals, wired or wireless transfer of patient data.		2 RCTs – Internet interventions<br>6 RCTs - other networked interventions<br>1 RCT – Text messaging<br>1 RCT – combination of text and internet	introductory session followed by telemedicine follow-up which included symptoms monitoring, educational aspects and self-management plans.	
COPD Reviews					
Bolton 2011	Self- or carer-recorded physiological data<br>Transmission of data to healthcare provider using telecommunication (manual or automated)<br>Review of data with personalised feedback	6 included studies<br>Only 1 RCT including educational component/self-management relevant to meta-review.<br>COPD – GOLD stage 3 or 4<br>55% female, mean age 67 years	n.s.	4/6 studies (1 RCT) included educational component	Daily completion of educational modules and transmission of data in relevant RCT
Cruz 2014	Home telemonitoring – transmission of clinical data (physiological or symptoms) using information and communication technologies to be assessed by healthcare provider.	Mean age >65<br>All studies included COPD severity in inclusion criteria.<br>4/7 moderate to severe COPD<br>3/7 severe to very severe COPD and/or long term oxygen therapy	Home setting.<br>Self-recorded physiological data as well as monitoring of symptoms and exercise tolerance. Transmitted to healthcare professional using information communication technology.	n.s.	Duration 2-12 months<br>Daily transmission
Flodgren 2015	Telemedicine interventions grouped into *remote monitoring, store and forward applications,* and *interactive (real-time) telemedicine*.	Mean age 60 to 68	2 specifically designed monitoring devices (one hand-held, one home based).<br>One internet-based intervention.	All had educational focus and concerned review of clinical data with feedback	Follow-up 6-12 months<br>Data transfer weekly to fortnightly.
Franek 2012	Home telehealth technologies – self-recorded physiological	HTM:<br>3/5 specified severe COPD (and/or LTOT in one study)<br>Telephone support:	HTM:<br>Connected via landline<br>All included pulse oximetry plus other physiological recording equipment	HTM:<br>All included personalised feedback on symptom management	HTM:<br>1-12 month intervention<br>No additional follow-up

	data transmitted to healthcare professional using ICT<br>Telephone only support – regular contact between patient and professional by telephone or videoconference	COPD patients discharged from a single hospital	including FEV1 monitor, pedometer, thermometer<br>Telephone support:<br>Delivered post discharge	1/5 explicitly included self-management education<br>Telephone support:<br>Individualised post-discharge patient education	Telephone support:<br>18 day intervention, 15 day follow-up
Kujipers 2012	Web-based interventions for patient empowerment and physical activity	Mean age 60 years (all conditions)	Web based – included online interaction with professional – usually study nurse	Online support promoting self-management and physical activity programmes with peer support and professional feedback.	12 months
Lundell 2015	Home-based telehealthcare incorporating feedback, counseling or motivational elements.	Mean age ranged 64 to 73.	5 telephone interventions<br>4 web based<br>3 mobile interventions	Motivation or counseling elements of patient feedback common to all interventions included.	Feedback or motivation element at least 3 times in first months of intervention.<br>Up to 12 months follow-up
McLean 2011	Home telecare. Broad definition, included video, telephone or internet links with health professionals, wired or wireless transfer of patient data.	COPD<br>Classified as severe in 2 RCTs	3 telephone systems<br>3 internet delivery<br>3 videoconferencing<br>1 specialised independent network with video	Combination of educational self-management interventions combined with telemonitoring (2 RCTs) and home visits (2 RCTs)	Follow-up 6 to 12 months
Polisena 2010	Home telemonitoring (remote care delivery or monitoring) or telephone support (without electronic transmission of data)	COPD<br>Mean age ranged 67 to 72<br>Male:female ratio ranged 50:46 to 87:3	4 telemonitoring<br>6 telephone support	Follow-up 3 to 19 months	Daily to weekly contact<br>3 to 15 month follow-up
Cancer Reviews					
Beatty 2013	Internet based self-help programmes	1 RCT of 62 patients with breast cancer.<br>Mean age 52.5 and 50.3 (intervention and control respectively)	Web-based	Internet delivered cognitive behavioural therapy with email prompts	12 weeks duration

Kujipers 2012	Web-based interventions for patient empowerment and physical activity	1 RCT – 325 patients with breast and prostate cancer	Web based – included online interaction with professional	Self-monitoring plus tailored self-management support<br>Communication with other patients and with expert nurses via internet	12 months duration
McAlpine 2015	Online interventions for cancer patients. Various types but separately considered those facilitating communication between clinician and patient (only these data considered for meta-review)	2 RCT – various cancer types – 353 patients total<br>1RCT – Lung cancer – 79 patients – mean age 60<br>1 RCT – Breast cancer. 252 patients	All web based delivery.	One moderated internet0based CBT programme.<br>One post-thoracotomy symptom monitoring programme incorporating telephone elements as well as online.<br>Two online information portal.<br>/educational interventions with online interaction with clinician.	Follow-up 9 months in one RCT, otherwise n.s.
