# Supplementary material for: Telehealth Interventions to Support Self-Management of Long-Term Conditions: A Systematic Metareview of Diabetes, Heart Failure, Asthma, Chronic Obstructive Pulmonary Disease, and Cancer
Source: J Med Internet Res. 2017 May 17;19(5):e172. doi: 10.2196/jmir.6688 (PMC5451641; doi:10.2196/jmir.6688)
Supplement: Multimedia Appendix 4 [file jmir_v19i5e172_app4.pdf]

Additional File 3: R-AMSTAR quality assessment												
Review	Was an “a prioro” design provided?	action?Was there duplicate study selection and data	Was a comprehensive literature search performed?	n?Was the status of publication (i.e. grey literature)	ovided?Was a list of studies (included and excluded)	led?Were the characteristics of the included studies	ed?Was the scientific quality of the included studies	ns?Was the scientific quality of the included studies	?Were the methods used to combine the findings of	Was the likelihood of publication bias assessed?	Was the conflict of interest included?	Total
Baron 2012	3	1	3	2	3	4	4	4	1	1	2	28
Beatty 2013	3	4	4	2	2	4	4	4	1	1	2	31
Beratarrechea 2014	3	4	4	4	2	4	3	4	1	1	3	31
Bolton 2011	3	4	4	4	2	4	3	3	1	1	3	32
Cassimatis 2012	3	1	4	2	1	4	4	4	1	1	1	26
Chaudhry 2007	3	4	4	2	2	4	4	4	2	1	4	34
Ciere 2012	3	4	4	2	2	4	4	4	2	1	1	31
Clarke 2011	3	4	4	1	1	3	1	2	4	1	3	27
Cruz 2014	3	3	4	2	2	4	4	4	4	4	3	36
Currell 2000	4	4	4	3	4	3	4	4	2	2	4	38
De Jongh 2012	3	4	4	4	4	4	2	4	2	2	3	36
Farmer 2005	3	4	4	4	2	4	4	4	4	2	2	36
Farmer 2016	4	4	4	1	2	3	4	4	4	4	3	37
Flodgren 2015	4	4	4	4	4	4	4	4	4	4	4	44
Franek 2012	3	3	4	2	2	4	4	4	2	2	3	33
Garcia Lizana 2007	3	2	3	1	1	4	1	2	1	1	3	22
Graziano 2009	4	1	3	2	1	4	2	2	1	1	2	23
Greenwood 2014	3	1	4	2	2	2	1	1	1	1	3	21
Hamine 2015	4	4	2	2	1	2	1	2	1	1	3	23
Holtz 2012	3	4	3	2	1	2	1	1	1	1	3	22
Huang 2015	4	4	3	1	1	1	4	4	4	4	3	33
Inglis 2015	4	4	4	4	4	4	4	4	4	3	4	43
Jaana 2007	3	1	3	2	1	3	1	2	1	1	2	20
Jaana 2009	4	1	3	1	1	4	1	2	1	1	3	22
Kok 2011	3	2	4	1	1	4	4	4	1	1	3	28
Krishna 2008	3	1	4	2	2	3	1	2	1	1	3	21
Krishna 2009	3	1	2	1	2	4	1	2	1	1	1	19
Kujipers 2012	3	3	4	2	2	4	4	3	2	1	3	31
Liang 2010	3	4	4	2	2	4	3	2	4	3	3	34
Lieber 2014	3	1	2	1	2	4	1	1	4	1	2	22
Lundell 2015	4	4	4	2	2	4	4	4	4	3	4	39
Marcano Bellisario 2013	3	4	4	4	4	4	4	3	4	2	3	39
Marcolino 2013	4	4	4	3	2	4	4	4	4	3	3	39
McAlpine 2015	3	4	3	2	3	4	1	2	2	2	3	29
McLean 2010	3	4	4	4	4	4	4	4	4	3	4	42
McLean 2011	4	4	4	3	4	4	4	4	4	4	4	43

Medical Advisory Secretariat 2009	3	4	4	2	3	4	4	4	4	1	3	36
Montori 2004	4	2	3	1	1	3	1	1	4	1	3	24
Mushcab 2015	4	4	4	1	1	4	1	1	1	1	3	25
Polisena 2009	4	4	4	4	2	4	4	4	4	1	3	38
Polisena 2010	3	4	3	3	2	4	4	4	4	1	3	35
Radhakrishnan 2012	3	1	2	2	2	4	2	4	1	1	3	25
Saffari 2014	4	4	4	1	2	4	4	2	4	4	3	36
Schmidt 2010	3	1	4	2	1	4	2	2	1	1	3	24
Small 2013	4	4	3	2	1	4	4	4	4	1	3	34
Suksomboon 2014	4	4	4	1	2	4	4	2	4	4	3	36
Sutcliffe 2011	4	4	4	4	2	4	4	4	3	1	2	36
Verhoeven 2007	3	4	4	2	3	4	2	3	3	1	2	31
Verhoeven 2010	3	4	4	2	3	4	4	4	4	2	1	35
Viana 2016	4	4	4	2	1	4	4	4	4	1	3	35
Wens 2008	4	4	4	1	2	4	3	3	4	2	3	34
Wu 2010	4	4	4	1	2	4	4	3	4	4	4	38
Zhai 2014	3	4	4	2	3	4	4	3	4	4	3	38
