# Supplementary material for: Telehealth Interventions to Support Self-Management of Long-Term Conditions: A Systematic Metareview of Diabetes, Heart Failure, Asthma, Chronic Obstructive Pulmonary Disease, and Cancer
Source: J Med Internet Res. 2017 May 17;19(5):e172. doi: 10.2196/jmir.6688 (PMC5451641; doi:10.2196/jmir.6688)
Supplement: Multimedia Appendix 5 [file jmir_v19i5e172_app5.pdf]

Details of Included Studies

**Author and year**<br>RCTs/participants<br>R-AMSTAR<br>Disease group	**R-AMSTAR**<br>**Explicit/implied SM**	**Intervention**<br>TH intervention<br>Focus and components	**Results (Disease control)**<br>Meta-reviews report summary statistics Narrative syntheses: denominator is number of RCTs reporting outcome
**Diabetes Reviews**			
**Type 1 Diabetes Reviews**			
* **Baron 2012 [19]**<br>2 RCTs, n=1303	R-AMSTAR = 28<br>Implied SM	**TH:** Mobile TM of blood glucose<br>Focus: monitoring and provision of action plan	0/2 RCTS showed a significant improvement in HbA1c
*** Currell 2000[23]**<br>2 RCTs,n=148	R-AMSTAR = 38<br>Implied SM	**TH:** Internet and telephone interventions<br>Focus: monitoring and provision of action plan	1/2 RCTs showed significant improvement in HbA1c
**** De Jongh 2012 [24]**<br>2 RCTs, n = 130	R-AMSTAR = 36<br>Explicit SM	**TH:** Mobile phone messaging for SM support<br>Information and education (n=1), Adherence support (n=2)	Meta-analysis of HbA1c: no significant improvement vs control (MD -0.15%, 95%CI -0.77 to 0.47)
**Montori 2004 [41]**<br>8 RCTs, n=391	R-AMSTAR = 24<br>Implied SM	**TH:** TH facilitated communication with professional<br>Focus: monitoring with action plan	Meta-analysis of HbA1c: no significant difference vs usual care (MD 0.2%, 95%CI -0.2 to 0.6%)
*** Sutcliffe 2011 [48]**<br>9 RCTs	R-AMSTAR = 36<br>Implied SM	**TH:** TH aimed at improving access and management of young people with T1DM<br>Focus of SR was clinical review and advice; psychological support (n=2)	2/10 RCTs showed significant improvement in HbA1c
**** Viana 2016 [49]**<br>6 RCTs, n=494	R-AMSTAR = 35<br>Explicit SM	**TH:** Telemonitoring of blood glucose and telephone support<br>Focus: adherence support; information and education (n=2); monitoring and action plan (n=4)	Meta-analysis of HbA1c: no significant difference vs usual care (MD -0.124, 95%CI -0.268 to 0.020)
**Type 2 Diabetes Reviews**			
* **Baron 2012 [19]**<br>10 RCTs, n=1303	R-AMSTAR = 28<br>Implied SM	**TH:** Mobile TM of blood glucose<br>Focus: monitoring and provision of action plan	6/10 RCTs showed a significant improvement in HbA1c
***** Beatty 2013 [21]**<br>5 RCTs, n=1627	R-AMSTAR = 31<br>Explicit SM	**TH:** Internet based SM<br>Focus: lifestyle advice/support	0/4 RCTs showed a significant improvement in HbA1c
**** Cassimatis 2012 [22]**<br>13 RCTs	R-AMSTAR = 26<br>Explicit SM	**TH:** Behavioural support via video/telephone support<br>Focus: lifestyle advice/support; Adherence support (n=8)	4/13 RCTs showed a significant improvement in HbA1c
***** Farmer 2016 [26]**<br>11 RCTs, n=4820	R-AMSTAR = 37<br>Explicit SM	**TH:** Messaging and/or monitoring<br>Focus: adherence support	Meta-analysis of impact on adherence (5RCTs): ‘moderate’ effect, not statistically significant
**** Graziano 2009 [29]**<br>8 RCTs, n=2105	R-AMSTAR = 23<br>Explicit SM	**TH:** Isolated telephone interventions<br>Focus: information and education and clinical review with advice	3/8 RCTs showed significant reduction in HbA1c
**** Greenwood**	R-AMSTAR = 21	**TH:** Remote TM to support self-	HbA1c was improved in those

**2014 [30]**<br>15 RCTs	Explicit SM	monitoring of glucose<br>Focus: information and education; monitoring and action plan; clinical review and advice; and lifestyle advice/support	RCTs incorporating at least 5 of 7 pre-specified SM components. Greater reductions were seen in those with 6 of 7 components.
**** Medical Advisory Secretariat 2009 [42]**<br>8 RCTs, n=2269	R-AMSTAR = 36<br>Implied SM	**TH:** Home telemonitoring<br>Focus: monitoring and action plan; lifestyle advice/support (n=7)	Meta-analysis of HbA1c: significant reduction vs usual care (MD -0.48%, 95%CI -0.70 to -0.26)
*** Mushcab 2015 [43]**<br>9 RCTs	R-AMSTAR = 25<br>Explicit SM	**TH:** Web-based transmission of self-monitored blood glucose<br>Focus: monitoring and action plan	4/9 RCTs showed significant reduction in HbA1c
**** Saffari 2014 [46]**<br>10 RCTs, n=960	R-AMSTAR = 36<br>Explicit SM	**TH:** Mobile text-messaging<br>Focus: information and education	Meta-analysis of HbA1c: significant reduction vs control (MD -0.595%, 95% CI -0.833 to -0.356)
**** Wens 2008 [52]**<br>2 RCTs	R-AMSTAR = 34<br>Explicit SM	**TH:** TH mediated education interventions<br>Focus: information and education and adherence support	1/2 showed a significant reduction in HbA1c
***** Wu 2010 [53]**<br>7 RCTs, n=1764	R-AMSTAR = 38<br>Explicit SM	**TH:** Telephone follow-up<br>Monitoring and action plan (n=5); clinical review and advice (n=5); psychological support (n=2); lifestyle advice/support (n=2)	Meta-analysis of HbA1c: no significant difference vs usual care (MD -0.44%, 95%CI -0.93 to 0.06). Planned subgroup analysis of more intensive interventions showed significant improvement (MD -0.84% 95%CI -1.67 to 0.0)
**** Zhai 2014 [54]**<br>35 RCTs	R-AMSTAR = 38<br>Implied SM	**TH:** Home telemonitoring<br>Focus of SR was monitoring and action plan	Meta-analysis of HbA1c: significant reduction vs control (MD -0.37%, 95%CI -0.49% to -0.25%)
**Mixed Diabetes Reviews**			
*** Beratarrechea 2014 [20]**<br>3 RCTs	R-AMSTAR = 31<br>Implied SM	**TH:** Mobile interventions in developing countries<br>Information and education (n=2), monitoring and action plan (n=1)	2/3 RCTs showed a significant improvement in 'glycaemic control', but unclear how this was measured
***** Farmer 2005 [25]**<br>12 RCTs, n=1038	R-AMSTAR = 36<br>Explicit SM	**TH:** TM supporting blood glucose self-monitoring<br>Focus: monitoring and provision of action plan	Meta-analysis of HbA1c (9RCTs): No significant reduction in HbA1c vs control (MD -0.1%, 95%CI -0.4% to 0.04)
**** Flodgren 2015 [27]**<br>21 RCTs, n=3412	R-AMSTAR = 44<br>Implied SM	**TH:** Interactive TH excluding telephone-only interventions<br>Focus: clinical review and advice; information and education (n=11)	Meta-analysis of HbA1c (16RCTs): Significant reduction vs usual care (MD -0.31, 95%CI -0.37 to -0.24)
**** Garzia-Lizana 2007 [28]**	R-AMSTAR = 22<br>Explicit SM	**TH:** TH intervention excluding telephone-only	1/7 RCTs showed significant reduction in HbA1c

7 RCTs, n=1044		Information and education (n=3); Monitoring and action plan (n=4)	
**** Hamine 2015 [31]**<br>26 RCTs	R-AMSTAR = 23<br>Explicit SM	**TH:** Mobile interventions<br>Focus of SR was medication adherence support	11/26 RCTs showed improved glycaemic control
*** Holtz 2012 [32]**<br>7 RCTs, n=417	R-AMSTAR = 22<br>Explicit SM	**TH:** Mobile interventions<br>Information and education (n=3); monitoring and action plan (n=6)	2/7 RCTs showed a significant improvement in HbA1c
**** Huang 2015 [33]**<br>18 RCTs, n=3798	R-AMSTAR = 33<br>Implied SM	**TH:** Transmission of self-monitored blood glucose<br>Focus: monitoring with action plan	Meta-analysis of HbA1c: significant reduction vs control (MD -0.54%, 95%CI -0.75 to -0.34
*** Jaana 2007 [34]**<br>13 RCTs, n-889	R-AMSTAR = 20<br>Implied SM	**TH:** Home telemonitoring<br>Focus of SR was monitoring and action plan; lifestyle advice/support (n=3)	7/13 showed significant improvements in HbA1c
**** Kok 2011 [37]**<br>9 RCTs, n-2223	R-AMSTAR = 28<br>Explicit SM	**TH:** TH intervention for SM education<br>Focus: information and education; monitoring and action plan (n=4)	8/9 showed significant improvement in HbA1c (5 were intervention plus usual care, 4 were intervention in place of usual care)
*** Krishna 2008 [35]**<br>8 RCTs, n=271	R-AMSTAR = 21<br>Explicit SM	**TH:** Mobile phone SM support<br>Focus of SR was information and education support and monitoring with action plan	5/6 showed significant improvement in HbA1c<br>Intermediate outcomes reported as improved self-efficacy in 1/1 RCT
*** Krishna 2009 [36]**<br>9 RCTs, n=331	R-AMSTAR = 19<br>Explicit SM	**TH:** Mobile phone SM support and education<br>Focus of SR was information and education; monitoring and action plan (n=7); adherence support (n=7); and lifestyle advice (n=7)	7/8 RCTs showed significant improvement in HbA1c
***** Kujipers 2012 [16]**<br>11 RCTs	R-AMSTAR = 31<br>Explicit SM	**TH:** Web based interventions<br>Focus: psychological support and lifestyle advice/support	3/6 RCTs showed significant improvement in self-efficacy
***** Liang 2011 [38]**<br>11 RCTs<br>n-1060	R-AMSTAR = 34<br>Explicit SM	**TH:** Mobile phone interventions<br>Focus of SR was lifestyle advice/support; "most studies" included monitoring and action plan	Meta-analysis of HbA1c: significant reduction vs usual care (MD -0.5%, 95% CI -0.2 to -0.8%)<br>Effect more marked for T2DM than T1DM
*** Lieber 2014 [39]**<br>5 RCTs	R-AMSTAR = 22<br>Implied SM	**TH:** TM of self-monitored blood glucose<br>Focus of SR was monitoring with action plan	1/5 RCTs showed significant improvement in HbA1c
**** Marcolino 2013[40]**<br>13 RCTs, n=4207	R-AMSTAR = 39<br>Implied SM	**TH:** TH facilitated communication with professional<br>Focus of SR was information and education and clinical review with advice	Meta-analysis of HbA1c: significant reduction versus control (MD -0.44%, 95%CI -0.61 to -0.26%). Effect more marked for T1DM
**** Polisena 2009 [44]**	Implied SM<br>R-AMSTAR = 38	**TH:** Home TH (subdivided telemonitoring and telephone	Meta-analysis of HbA1c in home telemonitoring:

16 RCTs, n-1671		support)<br>Information and education (n=5), monitoring and action plan (n=13), lifestyle advice/support (n=4)	significant reduction vs usual care (MD -0.21%, 95%CI -0.35% to -0.08%)*
***** Small 2013 [45]**<br>7 RCTs, n=1807	R-AMSTAR = 34<br>Explicit SM	**TH:** Telephone interventions using peer support or “lay health workers”<br>Focus of SR was information and education; psychological support (n=3), lifestyle advice/support (n=4)	Meta-analysis of HbA1c: significant reduction vs control (MD -0.26, 95%CI -0.41 to -0.11)
*** Suksomboon 2014 [47]**<br>5 RCTs, n=953	Implied SM<br>R-AMSTAR = 36	**TH:** Telephone-only interventions<br>Information and education (n=2), Clinical review and advice (n=3), Adherence support (n=3)	Meta-analysis of HbA1c: no significant improvement vs usual care (MD -0.38%, 95%CI -0.91 to 0.16)
*** Verhoeven 2007 [50]**<br>11 RCTs	R-AMSTAR = 31<br>Implied SM	**TH:** Teleconsultation and videoconferencing<br>Focus of SR was clinical review and advice	Meta-analysis of HbA1c: no significant reduction vs usual care (MD 0.03%, 95%CI -0.31 to 0.24%)
**** Verhoeven 2010 [51]**<br>28 RCTs	R-AMSTAR = 35<br><br>Implied SM	**TH:** Synchronous and Asynchronous teleconsultation<br>Focus: clinical review and advice	Meta-analysis of HbA1c: no significant reduction vs controls (MD -0.10%, 95%CI -0.39 to 0.18%)
**Heart Failure Reviews**			
*** Beratarrechea 2014 [20]**<br>1 RCT	R-AMSTAR = 31<br>Implied SM	**TH:** Mobile phone interventions in developing countries<br>Information and education (n=1), lifestyle advice/support (n=1)	Improved 6 minute walk test in 1 RCT
**** Chaudhry 2007 [55]**<br>5 RCTs, n=2623	R-AMSTAR = 34<br>Implied SM	**TH:** any telemonitoring or telephone intervention<br>Focus: information and education and adherence support	0/5 showed reduced mortality vs control<br>3/5 showed reduced heart failure hospitalisation<br>2/5 showed reduced all-cause hospitalisation
***** Ciere 2012 [56]**<br>11 RCTs	R-AMSTAR = 31<br>Explicit SM	**TH:** telehealth interventions excluding telephone-only<br>Focus of SR was information and education and monitoring with action plans	Authors analysed evidence linking interventions to knowledge, self-care behaviours, and self-efficacy. Evidence was either lacking or too ambiguous to draw conclusions.
*** Clarke 2011 [57]**<br>13 RCTs, n=3480	R-AMSTAR = 27<br>Implied SM	**TH:** telemonitoring using specialised equipment<br>Focus: monitoring with action plan and adherence support	Meta-analyses: significant reduction vs control in: mortality (RR 0.77 (95% CI 0.61 to 0.97)) – primary outcome, heart failure specific hospital admission (RR 0.73 (95% CI 0.62-0.87))<br>No significant reduction in: all-cause hospital admission (RR 0.99 (95% CI 0.88-1.11)), emergency dept. visits (RR1.04 (95% CI 0.86-1.26))

**** Garcia-Lizana 2007 [28]**<br>6 RCTs, n=1086	R-AMSTAR = 22<br>Explicit SM	**TH:** TH intervention excluding telephone-only<br>information and education (n=1), monitoring and action plan (n=1), clinical review and advice (n=4)	2/3 showed reduced mortality<br>1/2 showed reduced hospitalisations<br>2/2 showed reduced emergency dept. visits<br>2/3 showed improved treatment adherence
**** Inglis 2015 [58]**<br>41 RCTs, n=13192	R-AMSTAR = 42<br>Implied SM	**TH:** structured telephone support and physiological telemonitoring<br>Focus: monitoring and action plan and clinical review with advice; information and education (n=4)	Meta-analyses: both telemonitoring and telephone support reduced all-cause mortality (RR 0.80, 95%CI 0.68 to 0.94 and RR 0.87, 95% CI 0.77 to 0.98, respectively) and heart-failure hospitalisations (RR 0.71, 95% CI 0.60 to 0.83 and RR 0.87, 95% CI 0.77 to 0.98, respectively) but not all-cause hospitalisations (RR 0.95, 95% CI 0.90 to 1.00 and RR 0.95, 95% CI 0.89 to 1.01, respectively)
**** Kuijpers 2012 [16]**<br>3 RCTs, n=165	R-AMSTAR = 31<br>Explicit SM	**TH:** Web-based interventions<br>Focus: lifestyle advice and support	1/1 RCT showed improved self-care in both intervention and control groups, but with no significant difference<br>0/1 RCT showed improved self-efficacy
*** Radhakrishnan 2012 [59**<br>8 RCTs, n=835	R-AMSTAR = 25<br>Explicit SM	**TH:** Interactive telemonitoring or educational interventions<br>information and education (n=4), clinical review and advice (n=4)	No sustained improvements in self-care in RCT data
*** Schmidt 2010 [60]**<br>19 RCTs	R-AMSTAR = 24<br>Implied SM	**TH:** Home telemonitoring<br>Focus: monitoring with action plans	3/3 reported improved medication compliance with telemonitoring
**Asthma Reviews**			
*** Beratarrechea 2014 [20]**<br>2 RCTs	R-AMSTAR = 31<br>Implied SM	**TH:** Mobile phone interventions in developing countries<br>Monitoring and action plan (n=2)	1/1 RCT reported improved FEV1 and symptoms scores<br>1/1 RCT reported reduced hospitalisation and emergency dept. visits
**** De Jongh 2012 [24]**<br>1 RCT, n-16	R-AMSTAR = 36<br>Explicit SM	**TH:** Mobile phone messaging interventions<br>Monitoring and action plan (n=1), adherence support (n=1)	1 RCT reported improvements in symptom score, hospital admissions and PEF variability.<br>Clinic visits higher in intervention group
**** Flodgren 2015 [27]**<br>5 RCTs, n=825	R-AMSTAR = 44<br>Explicit SM	**TH:** Interactive TH excluding telephone-only interventions<br>Focus of SR was clinical review and advice; information and education (n=5)	0/4 showed improved symptom scores<br>0/3 showed improved spirometry tests<br>1/4 showed increased clinic visits in intervention group
*** Garcia-Lizana 2007 [28]**	R-AMSTAR = 22<br>Explicit SM	**TH:** TH interventions excluding telephone-only	2/5 reported improved symptom scores

5RCTs, n=733		Information and education (n=5)	2/4 reported reduced unscheduled healthcare utilisation
*** Jaana 2009 [61]**<br>7 RCTs	R-AMSTAR = 22<br>Explicit SM	**TH:** Home telemonitoring<br>Monitoring and action plan (n=6); clinical review and advice (n=7); adherence support (n=3)	5/7 reported improved symptoms
*** Krishna 2009 [36]**<br>1 RCT, n=16	R-AMSTAR = 21<br>Explicit SM	**TH:** Mobile phone messaging with educational focus<br>Focus of SR was clinical review and advice; education and information	1/1 reported improved symptoms and reduced medication use
**** Marcano Belisario 2013 [62]**<br>2 RCTs, n-408	R-AMSTAR = 39<br>Explicit SM	**TH:** Smartphone applications<br>Focus: monitoring and action plans	0/1 reported improved symptoms<br>1/2 reported improved health-related QOL<br>1/2 reported reduced emergency dept. visits<br>0/2 showed reduced hospital admissions
**** McLean 2010 [63]**<br>21 RCTs, n=12038	R-AMSTAR = 42<br>Implied SM	**TH:** Home-based TH including telemonitoring and structured telephone support<br>Focus: monitoring and action plans and information and education	Meta-analyses: significant reduction versus control in hospitalisation after 12 months (OR 0.21 (95%CI 0.0 to 0.61)).<br>No significant reduction in emergency department visits or hospitalisation after 3 months (OR 1.16 (95%CI 0.52 to 2.58) and 0.47 (95%CI 0.01 to 36.46), respectively).<br>Improvement in health-related QOL was below clinically significant threshold.
**COPD Reviews**			
*** Bolton 2011 [64]**<br>2 RCTs, n=139	R-AMSTAR = 32<br>Implied SM	**TH:** Interactive physiological telemonitoring<br>Focus: monitoring and action plan; information and education (n=1)	1/1 reported improved QOL (St George's Respiratory Questionnaire)<br>1/1 reported fewer hospital admissions and emergency dept. visits<br>No significant reduction in exacerbation frequency
*** Cruz 2014 [65]**<br>7 RCTs, n=392	R-AMSTAR = 36<br>Implied SM	**TH:** Home telemonitoring<br>Focus: monitoring with action plan	Meta analyses: statistically significant improvement vs control in hospitalisation rate (– RR 0.72 (95%CI 0.53 to 0.98)) and QOL using SGRQ (SMD -0.53 (95%CI -0.97 to -0.09))<br>No significant difference in mean number of hospitalisations (SMD -0.06 (95%CI -0.32 to 0.19)) emergency dept. visits (RR

			0.68 (95%CI 0.38 to 1.18)) and mortality (RR=1.43, 95%CI 0.40-5.03)
**** Flodgren 2015 [27]**<br>3 RCTs, n=130	R-AMSTAR = 44<br>Explicit SM	**TH:** Interactive TH excluding telephone-only interventions<br>Focus: clinical review and advice; information and education (n=3)	1/1 reported no difference in healthcare utilisation<br>1/1 reported no difference in symptom score<br>1/1 reported improved health related QOL
*** Franek 2012 [66]**<br>6 RCTs, n=310	R-AMSTAR = 33<br>Implied SM	**TH:** Home telemonitoring and telephone-only support<br>Focus: monitoring and action plan; information and education (n=2)	2/6 reported reduced hospitalisation<br>1/3 reported reduced emergency dept. visits<br>2/2 reported improved health related QOL<br>0/1 reported improved mortality<br>0/1 reported reduced exacerbations<br>1/1 reported improved self-efficacy
**** Kuijpers 2012 [16]**<br>2 RCTs	R-AMSTAR = 31<br>Explicit SM	**TH:** Internet-based interventions<br>Focus: lifestyle advice/support; psychological support (n=1)	1/2 reported significant improvement in self-efficacy
**** Lundell 2015 [67]**<br>9 RCTs, n=982	R-AMSTAR = 39<br>Explicit SM	**TH:** Interactive telemonitoring or counselling<br>Focus: clinical review and advice	Meta-analyses: significant improvement vs control in time spent physically active (MD 64.7mins, 95%CI 54.4 to74.9)<br>No significant difference in exercise tolerance (MD 1.3 m (95% CI -8.1 to 5.5)) and dyspnoea score (MD 0.088 (95% CI 0.056 to 0.233))
**** McLean 2011 [68]**<br>10 RCTs, n=1004	R-AMSTAR = 43<br>Implied SM	**TH:** Home-based TH including telemonitoring and structured telephone support<br>Focus: monitoring and action plan; information and education (n=4)	Meta-analyses: significant reduction vs control in hospitalisations (OR 0.27 (95% CI 0.11 to 0.66) and emergency dept. visits OR 0.46 (95%CI 0.33 to 0.65)<br>No significant difference in mortality (OR 1.05 (95%CI 0.63 to 1.75)) or QOL (MD in SGRQ. -6.57 (95%CI -13.62 to 0.48))
*** Polisena 2010 [69]**<br>7 RCTs, n=697	R-AMSTAR = 35<br>Implied SM	**TH:** Home telemonitoring and telephone support<br>Focus: monitoring with action plan	Meta-analysis: no significant difference in mortality between telephone support and control (RR 1.07 (95% CI 0.70 to 1.62))*<br>No overall improvement in QOL with home telemonitoring<br>With telephone support 5/5 reported fewer

			hospitalisations and 4/4 reported fewer emergency dept. visits
**Cancer Reviews**			
**** Beatty 2013 [21]**<br>1 RCT, n=62<br>Breast cancer	R-AMSTAR=31<br>Explicit SM	**TH:** Moderated internet-based self-help<br>Focus: lifestyle advice/support; psychological support (n=1)	0/1 showed improvements in QOL or 'emotional wellbeing'
****Kuijpers 2012 [16]**<br>1 RCT, n=325<br>Breast cancer and prostate cancer	R-AMSTAR=31<br>Explicit SM	**TH: I**nternet-based interventions<br>Focus: lifestyle advice/support	No significant improvement in patient empowerment
*** McAlpine 2015 [70]**<br>4 RCTs<br>Cancer (lung n=1, breast n=1, various n=2)	R-AMSTAR= 29<br>Explicit SM	**TH:** Online education programmes linking patient with clinician<br>Focus: information and education	0/2 reported improved QOL<br>1/2 reported improved symptom scores
**Abbreviations**<br>CI – confidence interval; COPD – Chronic Obstructive Pulmonary Disease; DM- diabetes mellitus; HF – heart failure; MD – mean difference; PEF – Peak expiratory flow; RCT – randomised controlled trial; RR – Relative risk; SGRQ - St George's Respiratory Questionnaire; SR – Systematic review; T1DM – type 1 diabetes mellitus; T2DM – type 2 diabetes mellitus; TH – Telehealth			
*The risk ratio was originally published as 1.21 (95%CI 0.84 to 1.75), however this was shown to have been the result of an error which was subsequently identified and corrected[71, 72].			
