# Supplementary material for: Telehealth Interventions to Support Self-Management of Long-Term Conditions: A Systematic Metareview of Diabetes, Heart Failure, Asthma, Chronic Obstructive Pulmonary Disease, and Cancer
Source: J Med Internet Res. 2017 May 17;19(5):e172. doi: 10.2196/jmir.6688 (PMC5451641; doi:10.2196/jmir.6688)
Supplement: Multimedia Appendix 6 [file jmir_v19i5e172_app6.pdf]

**Author and year**<br>RCTs/participants<br>R-AMSTAR<br>Disease group	**Intervention**<br>TH intervention<br>SM focus and components	**Results**<br>Meta-reviews report summary statistics<br>Narrative syntheses: denominator is number of RCTs reporting outcome	**Harvest plot decision**<br>*Any important quality concerns ([SR] = Systematc review author, [MR] = Meta-review author)*
**Diabetes Reviews**			
* **Baron 2012**<br>12 RCTs, n=1303<br>R-AMSTAR = 28<br>T1DM and T2DM	**TH:** Mobile TM of blood glucose<br>**Implied SM:** Focus of SR was monitoring and provision of action plan	T1DM: 0/2 RCTS showed a significant improvement in HbA1c<br>T2DM: 6/10 RCTs showed a significant improvement in HbA1c	**T1DM:** illustrated as neutral<br>**T2DM:** Illustrated as positive (hatched)<br>*Evidence inconsistent.[SR]*<br>*Publication bias not assessed [MR]*
***** Beatty 2013**<br>5 RCTs, n=1627<br>R-AMSTAR = 31<br>T2DM	**TH:** Internet based SM<br>**Explicit SM:** Focus of SR was lifestyle advice/support	0/4 RCTs showed a significant improvement in HbA1c<br>Intermediate outcomes reported as improved: diet in 2/5; activity in 1/5; self-efficacy in 1/5	**T2DM:** illustrated as neutral<br>*Effect size of primary outcomes not assessed [MR]*
*** Beratarrechea 2014**<br>3 RCTs<br>R-AMSTAR = 31<br>DM 1and2	**TH:** Mobile interventions in developing countries<br>**Implied SM**: Information and education (n=2), monitoring and action plan (n=1)	2/3 RCTs showed a significant improvement in 'glycaemic control', but unclear how this was measured	**Excluded –** unclear if HbA1c<br><br>*Publication bias not assessed [MR]*
**** Cassimatis 2012**<br>13 RCTs<br>R-AMSTAR = 26<br>DM 1and2	**TH:** Behavioural support via video/telephone support<br>**Explicit SM:** Focus of SR was lifestyle advice/support; Adherence support (n=8)	4/13 RCTs showed a significant improvement in HbA1c<br>Intermediate outcomes reported as improved: diet in 5/8; activity in 5/8; self-care in 3/13; medication adherence in 3/8	**T1/2DM:** illustrated as neutral<br>*2/13 reported relationships between intervention intensity and outcomes; both showed greater improvement with more substantial intervention [SR]*
*** Currell 2000**<br>2 RCTs,n=148<br>R-AMSTAR = 38<br>T1DM	**TH:** Internet and telephone interventions<br>**Implied SM:** Focus of SR was monitoring and provision of action plan	1/2 RCTs showed significant improvement in HbA1c<br>Intermediate outcomes reported as decreased family problem-solving in 1 RCT	**T1DM:** illustrated as neutral<br>*Relatively old review – interventions in early stages and in development. Evidence likely to have been superseded.[MR]*
**** De Jongh 2012**<br>2 RCTs, n = 130<br>R-AMSTAR = 36<br>T1DM	**TH:** Mobile phone messaging for SM support<br>**Explicit SM:** Information and education (n=1), Adherence support (n=2)	Meta-analysis of HbA1c: no significant improvement vs control (MD -0.15%, 95%CI -0.77 to 0.47)<br>Intermediate outcomes: No change in complications in 1 RCT, no change in healthcare utilisation in 1 RCT	**T1DM:** illustrated as neutral<br>*Most studies provided insufficient information to assess the risk of bias.[SR]*
***** Farmer 2005**<br>12 RCTs, n=1038<br>R-AMSTAR = 36<br>DM 1 and 2	**TH:** TM supporting blood glucose self-monitoring<br>**Explicit SM:** Focus of SR was monitoring and provision of action plan	Meta-analysis of HbA1c (9RCTs): No signficiant reduction in HbA1c vs control (MD -0.1%, 95%CI -0.4% to 0.04)<br>Intermediate outcomes: no difference in healthcare utilisation	**T1/2DM:** illustrated as neutral<br>*Review authors highlight poor description of trial methodologies [SR]*
***** Farmer 2016**<br>11 RCTs, n=4820<br>R-AMSTAR = 37<br>T2DM	**TH:** Messaging and/or monitoring<br>**Explicit SM:** Focus of SR was adherence support	Meta-analysis of impact on adherence (5RCTs): 'moderate' effect, not statistically significant<br>Narrative synthesis: improved adherence in 6/15 interventions	**Excluded –** no control outcomes<br>*Self-reported measures of adherence may not be reliable. High risk of bias in "the majority of" included studies. [SR]*
**** Flodgren 2015**<br>21 RCTs, n=3412<br>R-AMSTAR = 44<br>DM 1 and 2	**TH:** Interactive TH excluding telephone-only interventions<br>**Implied SM:** Focus of SR was clinical review and advice; information and education (n=11)	Meta-analysis of HbA1c (16RCTs): Significant reduction vs usual care (MD -0.31, 95%CI -0.37 to -0.24)<br>Intermediate outcomes reported as improved: healthcare usage in 1/5	**T1/2DM:** illustrated as positive
**** Garzia-Lizana 2007**<br>7 RCTs, n=1044<br>R-AMSTAR = 22<br>DM 1 and 2	**TH:** TH intervention excluding telephone-only<br>**Explicit SM:** Information and education (n=3); Monitoring and action plan (n=4)	1/7 RCTs showed significant reduction in HbA1c	**T1/2DM:** illustrated as neutral<br>*High degree of heterogeneity. Reduction in intensity of interventions if used in clinical practice may change efficacy.[SR]*<br>*Publication bias not assessed [MR]*

**** Graziano 2009**<br>8 RCTs, n=2105<br>R-AMSTAR = 23<br>T2DM	**TH:** Isolated telephone interventions<br>**Explicit SM:** Focus of SR was information and education and clinical review with advice	3/8 RCTs showed significant reduction in HbA1c	**T2DM:** illustrated as neutral<br>*Authors acknowledge that the mediating role of self-management is not assessed in their review.[SR] Publication bias not assessed [MR]*
**** Greenwood 2014**<br>15 RCTs<br>R-AMSTAR = 21<br>T2DM	**TH:** Remote TM to support self-monitoring of glucose<br>**Explicit SM:** Focus of SR was information and education; monitoring and action plan; clinical review and advice; and lifestyle advice/support	HbA1c was improved in those RCTs incorporating at least 5 of 7 pre-specified SM components. Greater reductions were seen in those with 6 of 7 components.	**T2DM:** illustrated as positive (hatched)<br>*Included participants with T2DM using insulin, for whom effects of titration may not truly reflect behaviour change. [SR] No quality assessment used.[MR]*
**** Hamine 2015**<br>26 RCTs<br>R-AMSTAR = 23<br>DM 1 and 2	**TH:** Mobile interventions<br>**Explicit SM:** Focus of SR was medication adherence support	11/26 RCTs showed improved glycaemic control<br>Intermediate outcomes reported as improved adherence in 7/13 RCTs	**T1/2DM:** illustrated as neutral<br>*Limitation of self-reported measures of adherence. Little evidence of theoretical frameworks. [SR]*<br>*Publication bias not assessed [MR]*
*** Holtz 2012**<br>7 RCTs, n=417<br>R-AMSTAR = 22<br>DM 1 and 2	**TH:** Mobile interventions<br>**Explicit SM:** Information and education (n=3); monitoring and action plan (n=6)	2/7 RCTs showed a significant improvement in HbA1c<br>Intermediate outcomes reported as 'modest' improvements in knowledge and self-efficacy in a 'small proportion' of RCTs	**T1/2DM:** illustrated as neutral<br>*Small sample sizes.[SR] Diversity of outcomes considered at a general level without further analysis of some specific outcomes. Publication bias not assessed [MR]*
**** Huang 2015**<br>18 RCTs, n=3798<br>R-AMSTAR = 33<br>T2DM	**TH:** Transmission of self-monitored blood glucose<br>**Implied SM:** Focus of SR was monitoring with action plan	Meta-analysis of HbA1c: significant reduction vs control (MD -0.54%, 95%CI -0.75 to -0.34)	**T2DM:** illustrated as positive<br><br>*Limited detail provided of included studied [MR]*
*** Jaana 2007**<br>13 RCTs, n-889<br>R-AMSTAR = 20<br>DM 1 and 2	**TH:** Home telemonitoring<br>**Implied SM:** Focus of SR was monitoring and action plan; lifestyle advice/support (n=3)	7/13 showed significant improvements in HbA1c<br>Intermediate outcomes reported as improved knowledge or self-care in 6/13	**T1/2DM:** illustrated as positive (hatched)<br>*Heterogeneity of patient populations and outcomes measures limit generalisability [SR] No quality assessment, Publication bias not assessed [MR]*
**** Kok 2011**<br>9 RCTs, n-2223<br>R-AMSTAR = 28<br>DM 1 and 2	**TH:** TH intervention for SM education<br>**Explicit SM:** Focus of SR was information and education; monitoring and action plan (n=4)	8/9 showed significant improvement in HbA1c (5 were intervention plus usual care, 4 were intervention in place of usual care)	**T1/2DM:** illustrated as positive<br>*"evidence not fully convincing because of limited number of studies available and the methodological limitations"[SR]. Publication bias not assessed [MR]*
*** Krishna 2008**<br>8 RCTs, n=271<br>R-AMSTAR = 21<br>DM 1 and 2	**TH:** Mobile phone SM support<br>**Explicit SM:** Focus of SR was information and education support and monitoring with action plan	5/6 showed significant improvement in HbA1c<br>Intermediate outcomes reported as improved self-efficacy in 1/1 RCT	**T1/2DM:** illustrated as positive<br>*Study heterogeneity. Concern about reliability of patient entered data. Small short-term statistically significant, changes in HbA1c may not be clinically significance [SR]. No quality assessment. Publication bias not assessed [MR]*
*** Krishna 2009**<br>9 RCTs, n=331<br>R-AMSTAR = 19<br>DM 1 and 2	**TH:** Mobile phone SM support and education<br>**Explicit SM:** Focus of SR was information and education; monitoring and action plan (n=7); adherence support (n=7); and lifestyle	7/8 RCTs showed significant improvement in HbA1c<br>Intermediate outcomes reported as improved insulin adherence in 1/1 RCT	**T1/2DM:** illustrated as positive<br>*Small sample sizes. Lack of cost information [SR]. No quality assessment. Publication bias not assessed [MR]*

	advice (n=7)		
***** Kujipers 2012**<br>11 RCTs<br>R-AMSTAR = 31<br>DM 1 and 2	**TH:** Web based intervention	3/6 RCTs showed significant improvement in self-efficacy	**Excluded:** no control outcome<br>*Limited description of methods in primary studies including randomisation process [SR]. Publication bias not assessed [MR]*
***** Liang 2011**<br>11 RCTs<br>n-1060<br>R-AMSTAR = 34<br>DM 1 and 2	**TH:** Mobile phone interventions<br>**Explicit SM:** Focus of SR was lifestyle advice/support; "most studies" included monitoring and action plan	Meta-analysis of HbA1c: significant reduction vs usual care (MD -0.5%, 95% CI -0.2 to -0.8%)<br>Effect more marked for T2DM than T1DM	**T1/2DM:** illustrated as positive<br>*Smaller trials showed greater effect – possible publication bias.[SR]*
*** Lieber 2014**<br>5 RCTs<br>R-AMSTAR = 22<br>DM 1 and 2	**TH:** TM of self-monitored blood glucose<br>**Implied SM:** Focus of SR was monitoring with action plan	1/5 RCTs showed significant improvement in HbA1c	**T1/2DM:**illustrated as neutral<br>*Considerable heterogeneity between studies.[SR] No quality assessment. Publication bias not assessed [MR]*
**** Marcolino 2013**<br>13 RCTs, n=4207<br>R-AMSTAR = 39<br>DM 1 and 2	**TH:** TH facilitated communication with professional<br>**Implied SM:** Focus of SR was information and education and clinical review with advice	Meta-analysis of HbA1c: significant reduction versus control (MD -0.44%, 95%CI -0.61 to -0.26%). Effect more marked for T1DM<br>Secondary outcomes reported as significant reductions in blood pressure (systolic and diastolic) and LDL cholesterol	**T1/2DM:** illustrated as positive<br>*May reflect different self-management strategies, or be confounded by age of participants and design/acceptability of telehealth intervention [SR]*
**Montori 2004**<br>8 RCTs, n=391<br>R-AMSTAR = 24<br>T1DM	**TH:** TH facilitated communication with professional<br>**Implied SM:** Focus of SR was monitoring with action plan	Meta-analysis of HbA1c: no significant difference vs usual care (MD 0.2%, 95%CI -0.2 to 0.6%)	**T1DM:** illustrated as neutral<br>*Early meta-analysis: few participants in the included studies. Conclusions assume 'telecare' is a single homogenous intervention [MR].*
**** Medical Advisory Secretariat 2009**<br>8 RCTs, n=2269<br>R-AMSTAR = 36<br>T2DM	**TH:** Home telemonitoring<br>**Implied SM:** Focus of SR was monitoring and action plan; lifestyle advice/support (n=7)	Meta-analysis of HbA1c: significant reduction vs usual care (MD -0.48%, 95%CI -0.70 to -0.26)	**T2DM:** illustrated as positive<br>*Possible confounding as content of intervention group training not clear. Significant heterogeneity between studies.[SR]<br>Publication bias not assessed [MR]*
*** Mushcab 2015**<br>9 RCTs<br>R-AMSTAR = 25<br>T2DM	**TH:** Web-based transmission of self-monitored blood glucose<br>**Explicit SM:** Focus of SR was monitoring and action plan	4/9 RCTs showed significant reduction in HbA1c	**T2DM:** illustrated as neutral<br>*Publication bias not assessed [MR]*
**** Polisena 2009**<br>16 RCTs, n-1671<br>R-AMSTAR = 38<br>DM 1 and 2	**TH:** Home TH (subdivided telemonitoring and telephone support)<br>**Implied SM: I**nformation and education (n=5), monitoring and action plan (n=13), lifestyle advice/support (n=4)	Meta-analysis of HbA1c in home telemonitoring: significant reduction vs usual care (MD -0.21%, 95%CI -0.35% to -0.08%)	**T1/2DM:** illustrated as positive<br>*Poor methodological quality of included studies [SR].<br>Publication bias not assessed [MR]*
**** Saffari 2014**<br>10 RCTs, n=960<br>R-AMSTAR = 36<br>T2DM	**TH:** Mobile text-messaging<br>**Explicit SM:** Focus of SR was information and education	Meta-analysis of HbA1c: significant reduction vs control (MD -0.595%, 95% CI -0.833 to -0.356)	**T2DM:** illustrated as positive<br>*Egger's analysis suggests high chance of publication bias[SR]*
***** Small 2013**<br>7 RCTs, n=1807<br>R-AMSTAR = 34<br>DM 1 and 2	**TH:** Telephone interventions using peer support or "lay health workers"<br>**Explicit SM:** Focus of SR was information and	Meta-analysis of HbA1c: significant reduction vs control (MD -0.26, 95%CI -0.41 to -0.11)<br>Intermediate outcomes reported as improved self-care behaviours in 4/4 RCTs	**T1/2DM:** illustrated as positive<br>*Publication bias not assessed [MR]*

	education; psychological support (n=3), lifestyle advice/support (n=4)		
*** Suksomboon 2014**<br>5 RCTs, n=953<br>R-AMSTAR = 36<br>DM 1 and 2	**TH:** Telephone-only interventions<br>**Implied SM:** Information and education (n=2), Clinical review and advice (n=3), Adherence support (n=3)	Meta-analysis of HbA1c: no significant improvement vs usual care (MD -0.38%, 95%CI -0.91 to 0.16)	**T1/2DM:** illustrated as neutral<br>*Variation in the contents and description of standard care in 5 trials. [SR] Studies in which active self-monitoring or medication adjustment by patients was part of the trial were excluded.[MR]*
*** Sutcliffe 2011**<br>9 RCTs<br>R-AMSTAR = 36<br>T1DM	**TH:** TH aimed at improving access and management of young people with T1DM<br>**Implied SM:** Focus of SR was clinical review and advice; psychological support (n=2)	2/10 RCTs showed significant improvement in HbA1c<br>Intermediate outcomes reported as improved self-care in 2 RCTs	**T1DM:** illustrated as neutral<br>*Breadth of study designs make magnitude of effects and effects on health difficult to determine. Little large scale high quality evidence identified. [SR]*<br>*Publication bias not assessed [MR]*
**** Viana 2016**<br>6 RCTs, n=494<br>R-AMSTAR = 35<br>T1DM	**TH:** Telemonitoring of blood glucose and telephone support<br>**Explicit SM:** Focus of SR was adherence support; information and education (n=2); monitoring and action plan (n=4)	Meta-analysis of HbA1c: no significant difference vs usual care (MD -0.124, 95%CI -0.268 to 0.020)	**T1DM:** illustrated as neutral<br>*Publication bias not assessed [MR]*
*** Verhoeven 2007**<br>11 RCTs<br>R-AMSTAR = 31<br>DM 1 and 2	**TH:** Teleconsultation and videoconferencing<br>**Implied SM:** Focus of SR was clinical review and advice	Meta-analysis of HbA1c: no significant reduction vs usual care (MD 0.03%, 95%CI -0.31 to 0.24%)	**T1/2DM:** illustrated as neutral<br>*Publication bias not assessed [MR]*
**** Verhoeven 2010**<br>28 RCTs<br>R-AMSTAR = 35<br>T1/2DM	**TH:** Synchronous and Asynchronous teleconsultation<br>**Implied SM:** Focus of SR was clinical review and advice	Meta-analysis of HbA1c: no significant reduction vs controls (MD -0.10%, 95%CI -0.39 to 0.18%)	**T1/2DM:** illustrated as neutral<br>*Publication bias not assessed [MR]*
**** Wens 2008**<br>2 RCTs<br>R-AMSTAR = 34<br>T2DM	**TH:** TH mediated education interventions<br>**Explicit SM:** Focus of review was information and education and adherence support	1/2 showed a significant reduction in HbA1c	**T2DM:** illustrated as neutral<br>*Search completed in 2002 as was subgroup analysis of previous Cochrane review.[MR]*
***** Wu 2010**<br>7 RCTs, n=1764<br>R-AMSTAR = 38<br>T2DM	**TH:** Telephone follow-up<br>**Explicit SM:** Monitoring and action plan (n=5); clinical review and advice (n=5); psychological support (n=2); lifestyle advice/support (n=2)	Meta-analysis of HbA1c: no significant difference vs usual care (MD -0.44%, 95%CI -0.93 to 0.06). Planned subgroup analysis of more intensive interventions showed significant improvement (MD -0.84% 95%CI -1.67 to 0.0)<br>Intermediate outcomes reported improved: BMI in 0/2, BP in 0/1, healthcare utilisation in 2/4, dietician attendance in ¼, podiatrist attendance in 1/4, self-efficacy in 2/3	**T2DM:** illustrated as neutral<br>*Only analysed studies which reported mean difference in HbA1c. Secondary outcome analysis limited by differences in data availability.[SR]*
**** Zhai 2014**<br>35 RCTs<br>R-AMSTAR = 38<br>T2DM	**TH:** Home telemonitoring<br>**Implied SM:** Focus of SR was monitoring and action plan	Meta-analysis of HbA1c: significant reduction vs control (MD -0.37%, 95%CI -0.49% to -0.25%)	**T2DM:** illustrated as positive<br>*Heterogeneity in length of follow-up. Lack of blinding in included studies.[SR]*
**Heart Failure Reviews**			
*** Beratarrechea 2014**	**TH:** Mobile phone interventions in developing	Improved 6 minute walk test in 1 RCT	**Excluded:** no control outcome<br>*Publication bias not assessed [MR]*

1 RCT<br>R-AMSTAR = 31<br>Heart failure	countries<br>**Implied SM:** information and education (n=1), lifestyle advice/support (n=1)		
**** Chaudhry 2007**<br>5 RCTs, n=2623<br>R-AMSTAR = 34<br>Heart failure	**TH:** any telemonitoring or telephone intervention<br>**Implied SM:** Focus of SR was information and education and adherence support	0/5 showed reduced mortality vs control<br>3/5 showed reduced heart failure hospitalisation<br>2/5 showed reduced all-cause hospitalisation	**HF:** illustrated as neutral<br>*Poor quality of data reporting in primary studies. High heterogeneity of interventions and technologies. [SR]*<br>*Publication bias not assessed [MR]*
***** Ciere 2012**<br>11 RCTs<br>R-AMSTAR = 31<br>Heart failure	**TH:** telehealth interventions excluding telephone-only<br>**Explicit SM:** Focus of SR was information and education and monitoring with action plans	Authors analysed evidence linking interventions to knowledge, self-care behaviours, and self-efficacy. Evidence was either lacking or too ambiguous to draw conclusions.	**Excluded:** no control outcome<br>*Included study quality was generally poor with concerns relating to study power and blinding of assessors [SR]. Publication bias not assessed [MR]*
*** Clarke 2011**<br>13 RCTs, n=3480<br>R-AMSTAR = 27<br>Heart failure	**TH:** telemonitoring using specialised equipment<br>**Implied SM:** Focus of SR was monitoring with action plan and adherence support	Meta-analyses: significant reduction vs control in: mortality (RR 0.77 (95% CI 0.61 to 0.97)) – primary outcome, heart failure specific hospital admission (RR 0.73 (95% CI 0.62-0.87))<br>No significant reduction in: all-cause hospital admission (RR 0.99 (95% CI 0.88-1.11)), emergency dept. visits (RR1.04 (95% CI 0.86-1.26))	**HF:** illustrated as positive<br>*Significant heterogeneity of studies. [SR] Little analysis of the role of self-management despite this being highlighted. No quality assessment. Publication bias not assessed [MR]*
**** Garcia-Lizana 2007**<br>6 RCTs, n=1086<br>R-AMSTAR = 22<br>Heart failure	**TH:** TH intervention excluding telephone-only<br>**Explicit SM:** information and education (n=1), monitoring and action plan (n=1), clinical review and advice (n=4)	2/3 showed reduced mortality<br>1/2 showed reduced hospitalisations<br>2/2 showed reduced emergency dept. visits<br>2/3 showed improved treatment adherence	**HF:** illustrated as positive<br>*Heterogeneity between interventions and technologies. Reduction in intensity of interventions if used in clinical practice may change efficacy.[SR]*<br>*Publication bias not assessed [MR]*
**** Inglis 2015**<br>41 RCTs, n=13192<br>R-AMSTAR = 42<br>Heart failure	**TH:** structured telephone support and physiological telemonitoring<br>**Implied SM:** Focus of SR was monitoring and action plan and clinical review with advice; information and education (n=4)	Meta-analyses: both telemonitoring and telephone support reduced all-cause mortality (RR 0.80, 95%CI 0.68 to 0.94 and RR 0.87, 95% CI 0.77 to 0.98, respectively) and heart-failure hospitalisations (RR 0.71, 95% CI 0.60 to 0.83 and RR 0.87, 95% CI 0.77 to 0.98, respectively) but not all-cause hospitalisations (RR 0.95, 95% CI 0.90 to 1.00 and RR 0.95, 95% CI 0.89 to 1.01, respectively)	**HF:** illustrated as positive<br>*Difficulty identifying outcomes from multiple publications[SR]*
**** Kuijpers 2012**<br>3 RCTs, n=165<br>R-AMSTAR = 31<br>Heart failure	**TH:** Web-based interventions<br>**Explicit SM:** Focus of SR was lifestyle advice and support	1/1 RCT showed improved self-care in both intervention and control groups, but with no significant difference<br>0/1 RCT showed improved self-efficacy	**Excluded:** no control outcomes<br>*Limited description of methods in primary studies including randomisation process.[SR]*<br>*Publication bias not assessed [MR]*
*** Radhakrishnan 2012**<br>8 RCTs, n=835<br>R-AMSTAR = 25<br>Heart failure	**TH:** Interactive telemonitoring or educational interventions<br>**Explicit SM:** information and education (n=4), clinical review and advice (n=4)	No sustained improvements in self-care in RCT data	**Excluded:** no control outcomes<br>*Conclusion based on results of trials lacking control groups. No quality assessment. Publication bias not assessed [MR]*
*** Schmidt 2010**<br>19 RCTs<br>R-AMSTAR = 24<br>Heart failure	**TH:** Home telemonitoring<br>**Implied SM:** Focus of SR was monitoring with action plans	3/3 reported improved medication compliance with telemonitoring	**Excluded:** no control outcomes<br>*High heterogeneity in telemonitoring definitions and in nature of interventions.[SR]*<br>*Publication bias not assessed [MR]*
**Asthma Reviews**			
*** Beratarrechea**	**TH:** Mobile phone	1/1 RCT reported improved FEV1 and	**Asthma:** illustrated as positive

**2014**<br>2 RCTs<br>R-AMSTAR = 31<br>Asthma	interventions in developing countries<br>**Implied SM:** Monitoring and action plan (n=2)	symptoms scores<br>1/1 RCT reported reduced hospitalisation and emergency dept. visits	*Publication bias not assessed [MR]*
**** De Jongh 2012**<br>1 RCT, n-16<br>R-AMSTAR = 36<br>Asthma	**TH:** Mobile phone messaging interventions<br>**Explicit SM:** Monitoring and action plan (n=1), adherence support (n=1)	1 RCT reported improvements in symptom score, hospital admissions and PEF variability.<br>Clinic visits higher in intervention group	**Asthma:** illustrated as positive<br>*"Extremely small sample size". Most studies provided insufficient information to assess the risk of bias.[SR]*
**** Flodgren 2015**<br>5 RCTs, n=825<br>R-AMSTAR = 44<br>Asthma	**TH:** Interactive TH excluding telephone-only interventions<br>**Explicit SM:** Focus of SR was clinical review and advice; information and education (n=5)	0/4 showed improved symptom scores<br>0/3 showed improved spirometry tests<br>1/4 showed increased clinic visits in intervention group	**Asthma:** illustrated as neutral
*** Garcia-Lizana 2007**<br>5RCTs, n=733<br>R-AMSTAR = 22<br>Asthma	**TH:** TH interventions excluding telephone-only<br>**Explicit SM:** Information and education (n=5)	2/5 reported improved symptom scores<br>2/4 reported reduced unscheduled healthcare utilisation	**Asthma:** illustrated as neutral<br>*High degree of heterogeneity between interventions and technologies. Reduction in intensity of interventions if used in clinical practice may change efficacy.[SR]*<br>*Publication bias not assessed [MR]*
*** Jaana 2009**<br>7 RCTs<br>R-AMSTAR = 22<br>Asthma	**TH:** Home telemonitoring<br>**Explicit SM:** Monitoring and action plan (n=6); clinical review and advice (n=7); adherence support (n=3)	5/7 reported improved symptoms<br>No consistent evidence of reduced healthcare utilisation (all respiratory conditions)	**Asthma:** illustrated as positive<br>*Limited detail on systematic review methodology. Publication bias not assessed [MR]*
*** Krishna 2009**<br>1 RCT, n=16<br>R-AMSTAR = 21<br>Asthma	**TH:** Mobile phone messaging with educational focus<br>**Explicit SM:** Focus of SR was clinical review and advice; education and information	1/1 reported improved symptoms and reduced medication use	**Asthma:** illustrated as positive<br>*Only one RCT, very small sample size. Publication bias not assessed [MR]*
**** Marcano Belisario 2013**<br>2 RCTs, n-408<br>R-AMSTAR = 39<br>Asthma	**TH:** Smartphone applications<br>**Explicit SM:** Focus of review was monitoring and action plans	0/1 reported improved symptoms<br>1/2 reported improved health-related QOL<br>1/2 reported reduced emergency dept. visits<br>0/2 showed reduced hospital admissions	**Asthma:** illustrated as neutral<br>*Inadequate information for one study to assess risk of bias [SR]*
**** McLean 2010**<br>21 RCTs, n=12038<br>R-AMSTAR = 42<br>Asthma	**TH:** Home-based TH including telemonitoring and structured telephone support<br>**Implied SM:** Focus of SR was monitoring and action plans and information and education	Meta-analyses: significant reduction versus control in hospitalisation after 12 months (OR 0.21 (95%CI 0.0 to 0.61)).<br>No significant reduction in emergency department visits or hospitalisation after 3 months (OR 1.16 (95%CI 0.52 to 2.58) and 0.47 (95%CI 0.01 to 36.46), respectively).<br>Improvement in health-related QOL was below clinically significant threshold.	**Asthma:** illustrated as neutral<br>*Poor randomisation procedure and overall variable quality of primary studies.[SR]*
**COPD Reviews**			
*** Bolton 2011**<br>2 RCTs, n=139<br>R-AMSTAR = 32<br>COPD	**TH:** Interactive physiological telemonitoring<br>**Implied SM:** Focus of SR was monitoring and action plan; information and education (n=1)	1/1 reported improved QOL (St George's Respiratory Questionnaire)<br>1/1 reported fewer hospital admissions and emergency dept. visits<br>No significant reduction in exacerbation frequency	**COPD:** illustrated as positive (hatched)<br>*High risk of bias in quality assessment within review. [SR]*<br>*RCTs: small sample sizes; only one including SM component.*<br>*Publication bias not assessed [MR]*
*** Cruz 2014**<br>7 RCTs, n=392<br>R-AMSTAR = 36<br>COPD	**TH:** Home telemonitoring<br>**Implied SM:** Focus of SR was monitoring with action plan	Meta analyses: statistically significant improvement vs control in hospitalisation rate (– RR 0.72 (95%CI 0.53 to 0.98)) and QOL using SGRQ (SMD -0.53 (95%CI -0.97 to -0.09))	**COPD:** illustrated as neutral<br>*Small sample sizes of RCTs (although good quality). Unable to incorporate data from some studies as it was not comparable.*

		No significant difference in mean number of hospitalisations (SMD -0.06 (95%CI -0.32 to 0.19)) emergency dept. visits (RR 0.68 (95%CI 0.38 to 1.18)) and mortality (RR=1.43, 95%CI 0.40-5.03)	*Exclusion of non-English studies (Portuguese and Spanish accepted). Unable to assess for publication bias.[SR]*
**** Flodgren 2015**<br>3 RCTs, n=130<br>R-AMSTAR = 44<br>COPD	**TH:** Interactive TH excluding telephone-only interventions<br>**Explicit SM:** Focus of SR was clinical review and advice; information and education (n=3)	1/1 reported no difference in healthcare utilisation<br>1/1 reported no difference in symptom score<br>1/1 reported improved health related QOL	**COPD:** illustrated as neutral
*** Franek 2012**<br>6 RCTs, n=310<br>R-AMSTAR = 33<br>COPD	**TH:** Home telemonitoring and telephone-only support<br>**Implied SM:** Focus of SR was monitoring and action plan; information and education (n=2)	2/6 reported reduced hospitalisation<br>1/3 reported reduced emergency dept. visits<br>2/2 reported improved health related QOL<br>0/1 reported improved mortality<br>0/1 reported reduced exacerbations<br>1/1 reported improved self-efficacy	**COPD:** illustrated as neutral<br>*Definitions of hospitalisation differed between studies. Low quality of evidence according to quality assessment. High level of heterogeneity limiting conclusions. [SR]*
**** Kuijpers 2012**<br>2 RCTs<br>R-AMSTAR = 31<br>COPD	**TH:** Internet-based interventions<br>**Explicit SM:** Focus of SR was lifestyle advice/support; psychological support (n=1)	1/2 reported significant improvement in self-efficacy	**Excluded:** no control outcomes<br>*Limited description of methods in primary studies including randomisation process.[SR]. Publication bias not assessed [MR]*
**** Lundell 2015**<br>9 RCTs, n=982<br>R-AMSTAR = 39<br>COPD	**TH:** Interactive telemonitoring or councelling<br>**Explicit SM:** Focus of SR was clinical review and advice	Meta-analyses: significant improvement vs control in time spent physically active (MD 64.7mins, 95%CI 54.4 to74.9)<br>No significant difference in exercise tolerance (MD 1.3 m (95% CI -8.1 to 5.5)) and dyspnoea score (MD 0.088 (95% CI 0.056 to 0.233))	**COPD:** illustrated as neutral
**** McLean 2011**<br>10 RCTs, n=1004<br>R-AMSTAR = 43<br>COPD	**TH:** Home-based TH including telemonitoring and structured telephone support<br>**Implied SM:** Focus of SR was monitoring and action plan; information and education (n=4)	Meta-analyses: significant reduction vs control in hospitalisations (OR 0.27 (95% CI 0.11 to 0.66) and emergency dept. visits OR 0.46 (95%CI 0.33 to 0.65)<br>No significant difference in mortality (OR 1.05 (95%CI 0.63 to 1.75)) or QOL (MD in SGRQ. -6.57 (95%CI -13.62 to 0.48))	**COPD:** illustrated as positive (hatched)<br>*Heterogeneity in definitions of COPD and interventions evaluated. Theoretical work and modelling of complex interventions unclear[SR]*
*** Polisena 2010**<br>7 RCTs, n=697<br>R-AMSTAR = 35<br>COPD	**TH:** Home telemonitoring and telephone support<br>**Implied SM:** Focus of SR was monitoring with action plan	Meta-analysis: no significant difference in mortality between telephone support and control (RR 1.07 (95% CI 0.70 to 1.62))*<br>No overall improvement in QOL with home telemonitoring<br>With telephone support 5/5 reported fewer hospitalisations and 4/4 reported fewer emergency dept. visits	**COPD:** illustrated as neutral<br>*Clinical heterogeneity due to diverse study populations and study design. Insufficient number of studies to assess publication bias. [SR]*
**Cancer Reviews**			
**** Beatty 2013**<br>1 RCT, n=62<br>R-AMSTAR=31<br>Breast cancer	**TH:** Moderated internet-based self-help<br>**Explicit SM:** Focus of SR was lifestyle advice/support; psychological support (n=1)	0/1 showed improvements in QOL or 'emotional wellbeing'	**Cancer:** illustrated as neutral<br>*Level of professional input is unclear – may not truly qualify as telehealth. [MR]*
****Kuijpers 2012**<br>1 RCT, n=325<br>R-AMSTAR=31<br>Breast cancer and prostate cancer	**TH:** Internet-based interventions<br>**Explicit SM:** Focus of SR was lifestyle advice/support	No significant improvement in patient empowerment	**Excluded:** no control outcomes<br>*Little evidence directed at cancer specifically. Limited description of methods in primary studies including randomisation process. [SR]*
*** McAlpine 2015**	**TH:** Online education	0/2 reported improved QOL	**Cancer:** illustrated as neutral

4 RCTs<br>R-AMSTAR= 29<br>Cancer (lung n=1, breast n=1, various n=2)	programmes linking patient with clinician<br>**Explicit SM:** Focus of SR was information and education	1/2 reported improved symptom scores	*No quality assessment [MR].*
**Abbreviations**<br>CI – confidence interval; COPD – Chronic Obstructive Pulmonary Disease; DM- diabetes mellitus; HF – heart failure; MD – mean difference; PEF – Peak expiratory flow; RCT – randomised controlled trial; RR – Relative risk; SGRQ - St George's Respiratory Questionnaire; SR – Systematic review; T1DM – type 1 diabetes mellitus; T2DM – type 2 diabetes mellitus; TH – Telehealth			
*The risk ratio was originally published as 1.21 (95%CI 0.84 to 1.75), however this was shown to have been the result of an error which was subsequently identified (ref) and corrected (ref).			
